# Anthranilic amide and imidazobenzothiadiazole compounds disrupt *Mycobacterium tuberculosis* membrane potential†
†Electronic supplementary information (ESI) available. See DOI: 10.1039/c9md00088g


**DOI:** 10.1039/c9md00088g

**Published:** 2019-05-03

**Authors:** Jake Smith, Heather Wescott, Julie Early, Steven Mullen, Junitta Guzman, Joshua Odingo, Jason Lamar, Tanya Parish

**Affiliations:** a TB Discovery Research , Infectious Disease Research Institute , 1616 Eastlake Ave E, Suite 400 , Seattle , Washington 98102 , USA . Email: tanya.parish@idri.org; b Lilly Research Laboratories , Eli Lilly and Company , 307 E Merrill St , Indianapolis , Indiana 46285 , USA

## Abstract

A family of compounds typified by an anthranilic amide **1** was identified from a whole-cell screening effort targeted at identifying compounds that disrupt pH homeostasis in *Mycobacterium tuberculosis*. **1** demonstrated bactericidal activity against non-replicating *M. tuberculosis* in pH 4.5 buffer (MBC_4.5_ = 6.3 μM). Exploration of the structure–activity relations failed to simplify the scaffold. The antitubercular activity proved dependent on the lipophilicity and planarity of the molecule and directly correlated with mammalian cytotoxicity. Further studies revealed a pH-dependent correlation between the family's disruption of *M. tuberculosis* membrane potential and antitubercular activity, with active compounds causing a drop in membrane potential at concentrations below their MBC_4.5_. A second compound family, identified in the same screening effort and typified by imidazo(4,5-*e*)(2,1,3)benzothiadiazole **2**, provided a contrasting profile. As with **1**, structure–activity profiling of **2** (MBC_4.5_ = 25 μM) failed to minimize the initial scaffold, mammalian cytotoxicity was observed for a majority of the active compounds, and many of the active compounds disrupted *M. tuberculosis* membrane potential. However, unlike the anthranilic amide compounds, the benzothiadiazole compounds disrupted *M. tuberculosis* membrane potential primarily at concentrations above the MBC_4.5_ in a pH-independent fashion. These differences suggest an alternative mechanism of action for the benzothiadiazole compounds. As a result, while the cytotoxicity of the anthranilic amides limits their utility to tool compounds, benzothiadiazole **2** presents an attractive target for more focused SAR exploration.

## Abstract 2

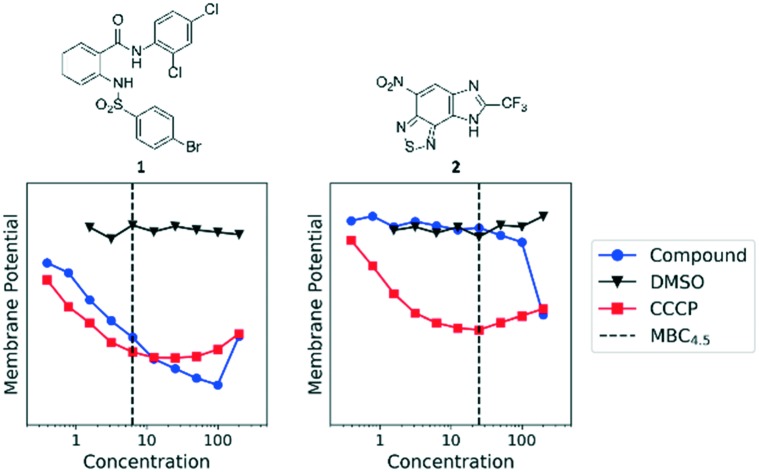
Compounds **1** and **2** disrupt *M. tuberculosis* membrane potential and demonstrate bactericidal activity against non-replicating *M. tuberculosis* in pH 4.5 buffer.

## Introduction

Tuberculosis is responsible for the most deaths annually of any single infectious agent with the majority of cases concentrated in lower to middle income countries.^1^ While global incidence and mortality rates declined slightly over the period 2005–2015,^2^ these gains are offset by increased incidence of multi-drug resistant (MDR) and extensively drug-resistant (XDR) cases.^3^ Chemotherapeutics with novel mechanisms of action are required to both shorten treatment of non-resistant tuberculosis and effectively combat the spread of MDR and XDR strains.^1^ An ideal characteristic of a new anti-mycobacterial therapeutic would be bactericidal activity against both replicating and non-replicating populations of bacteria.^4^–^6^ The ability to sterilize both populations is critical for clinical eradication of tuberculosis.

Survival of *Mycobacterium tuberculosis* within macrophages is dependent on the organism's ability to adapt and respond to numerous host environmental stresses encountered during infection including, but not limited to, reactive oxygen species, reactive nitrogen species, low pH, and nutrient restriction. Upon residing in resting macrophages, *M. tuberculosis* inhibits maturation of the phagosome and prevents phagolysosome fusion. The pH of the *M. tuberculosis*-containing vacuole is mildly acidic (pH 6.1–6.4) due to exclusion of the vacuolar proton-ATPase.^7^,^8^ Immunological activation of the macrophage removes the *M. tuberculosis*-mediated blockade of phagolysosomal fusion allowing the phagosome to become fully acidified (pH 4.5–5.4).^7^,^9^,^10^ The ability of *M. tuberculosis* to survive the acidic environments encountered during infection requires maintenance of cytoplasmic pH homeostasis. *M. tuberculosis* is capable of resisting phagolysosomal acid concentrations when grown in liquid medium buffered at pH 4.5–5.0 and is able to maintain pH homeostasis within IFN-γ-activated macrophages, indicating the presence of effective protective mechanisms for acid resistance.^11^,^12^ Acidic pH causes widespread alterations to the physiology of *M. tuberculosis*, including induction of numerous stress genes and the PhoPR regulon. *M. tuberculosis* encounters acidic pH at multiple stages during infection and these acidic environments likely act as a crucial cue for initiation of multiple adaptive mechanisms required for bacterial survival within the host.

We previously reported a whole-cell screening effort targeted at identifying compounds that disrupt pH homeostasis in *Mycobacterium tuberculosis*.^13^ We hypothesized that such compounds would prove bactericidal under acidic conditions. Five compound hits were selected and demonstrated to have pH-dependent bactericidal activity.^13^ Of the clustered hits resulting from this screen, we selected several for further development.

The first of these series is built upon on anthranilic amide core. The anthranilic amide scaffold has found use in small molecules targeted at a range of therapeutic applications: management of chronic pain^14^–^16^ and inflammation,^17^ reduction of cholesterol levels,^18^,^19^ inhibition of hepatitis C,^20^ and anticancer therapeutics.^21^,^22^ Peukert *et al.* identified a series of anthranilic amide-based Kv1.5 channel blockers with moderate oral bioavailability and no significant hERG inhibition.^23^,^24^ Rabinowitz *et al.* detailed the development of a series of cholecystokinin receptor agonists built around the anthranilic amide core.^25^–^29^ Finally, Kauppi *et al.* reported a series of related compounds with antibacterial activity against *Yersinia pseudotuberculosis*.^30^

The second of these series was a singleton hit with a tricyclic imidazo(4,5-*e*)(2,1,3)benzothiadiazole core. Reddy *et al.* reported a preliminary screening of related compounds for antimicrobial activity;^31^ however, the scaffold is largely unexplored in the context of medicinal chemistry.

Several potential mechanisms may be proposed for the pH-dependence of the screening hits, including changes in bacterial metabolic and proteomic state, increased intrabacterial accumulation of compounds, and compound instability under low pH conditions. From these potential mechanisms, ionophore activity is the least desirable, and similar screening projects have actively counter-screened against such compounds.^32^

Given a number of literature reports linking the disruption of pH homeostasis to the disruption of membrane potential in *M. tuberculosis*,^32^–^35^ our initial pursuits of compound series originating from our pH homeostasis screen included an evaluation of each series' effect on *M. tuberculosis* membrane potential as a surrogate for ionophore activity. We report development of the anthranilic amide and benzothiadiazole series toward a novel therapeutic, evaluation of their antitubercular activity, and insights into their mechanisms of action.

## Results and discussion

**Fig. 1:**
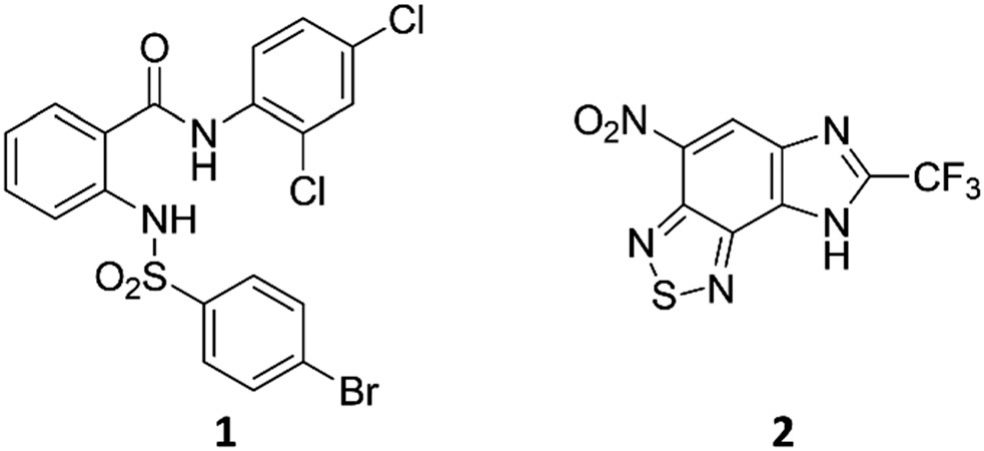
Structure of screening hits.

We previously identified compounds **1** and **2** as screening hits (Fig. 1). We wanted to evaluate the potential for developing these scaffolds into chemotherapeutics. We used a rapid method to determine antitubercular activity as our primary assay and confirmed activity using kill kinetics over 21 days with select compounds. We synthesized, in the case of the anthranilic acid family, or selected, in the case of the benzothiadiazole family, sets of approximately 30 compounds related to the respective screening hits. Compounds were tested for antitubercular activity and mammalian cytotoxicity in order to establish an understanding of the structure–activity relations (SAR). We tested the ability of select compounds from each series to disrupt the membrane potential of either *M. tuberculosis* or mammalian HepG2 cells.

**Fig. 2:**
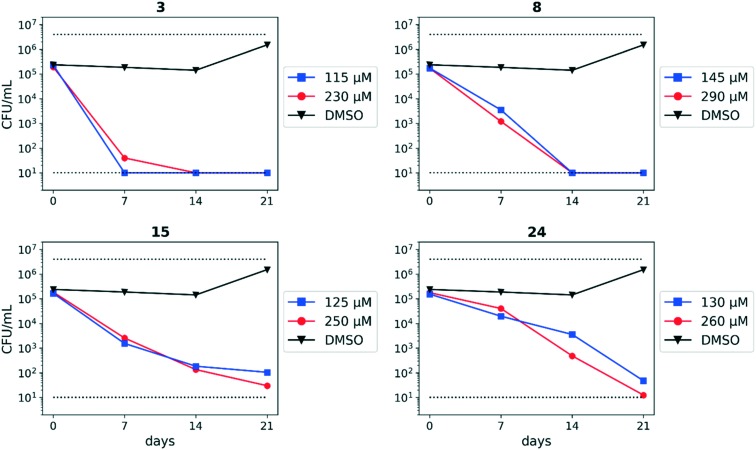
Bactericidal activity of anthranilic amide compounds against *M. tuberculosis*. Compounds were tested for their ability to kill non-replicating *M. tuberculosis* at pH 5.5. Dashed lines represent the upper and lower limits of detection. Reported values are a single replicate.

We assessed the antitubercular activity of a selection of anthranilic amide compounds under non-replicating conditions at pH 5.5 over 21 days (Fig. 2). All compounds tested were bactericidal. Compound **3** resulted in >3 log kill within 7 days and >4 log kill in 14 days. Compound **8** killed >4 logs at 14 days. Compounds **15** and **24** killed >3 logs within 21 days. All compounds showed the same pattern, with the same rate of kill independent of concentration, suggesting the activity is time-dependent.

From the benzothiadiazole series, the profile of compound **2** under non-replicating conditions at both pH 4.5 and pH 6.8 has been previously reported.^13^ Concentrations ranging from 10–100 μM decreased CFU in the culture >4 logs within 7 days, with all concentrations sterilizing the culture within 14 days.^13^

Development of screening hits **1** and **2** toward the goal of novel chemotherapeutics began with an investigation of the SAR. In each case, we aimed to establish both that variations on these core scaffolds produced corresponding changes in the antitubercular activity and that the antitubercular activity did not directly correlate with mammalian cytotoxicity.

In order to generate sufficient data for the SAR studies, we used a rapid method to determine bactericidal activity using luminescence as a reporter of bacterial viability. We used this method to determine the minimum concentration which resulted in a 2 log kill over 7 days. This method was less labor-intensive and allowed us to test more compounds rapidly. Since the readout for this assay is a threshold concentration rather than a value derived from a curve, the data are discrete rather than continuous. Therefore we report the median MBC_4.5_.

The SAR around screening hit **1** was interrogated by synthesis of a small set of analogs. For each compound, the MBC_4.5_ was determined as the primary measure of antitubercular activity, reported as the median of two or more independent runs. The IC_50_ against HepG2 cells was determined as the primary measure of mammalian cytotoxicity, reported as the mean ± standard deviation of two or more independent runs.

We first sought to identify the minimum pharmacophore present in screening hit **1**. To this end, the series of deconstructive analogs **3–9** were synthesized (Table 1). Both the amide and sulfonamide branches of the scaffold showed sharp declines in potency with minimization, with parent compound **1** remaining the most active example.

**Table 1:** Minimum pharmacophore determination. Compounds were tested for activity against *M. tuberculosis* (MBC_4.5_) and HepG2 cells (IC_50_). MBC_4.5_ are reported as the median. HepG2 IC_50_ are reported as the mean ± standard deviation. The number of replicates is in parentheses

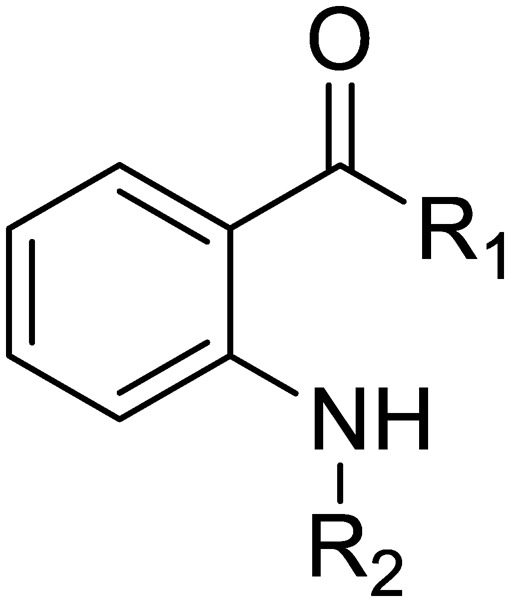					
Cpd	ID	R_1_	R_2_	MBC_4.5_ (μM)	HepG2 IC_50_ (μM)
**1**	IDR-0019306	2,4-Cl–Ph–NH-	4-Br–Ph–SO_2_-	6.3 (5)	6.2 ± 1.1 (3)
**3**	IDR-0597329	4-Cl–Ph–NH-	4-Br–Ph–SO_2_-	50 (2)	11 ± 4.7 (2)
**4**	IDR-0484542	PhNH-	4-Br–Ph–SO_2_-	100 (2)	30 ± 3.5 (2)
**5**	IDR-0597554	EtNH-	4-Br–Ph–SO_2_-	>200 (3)	>100 (3)
**6**	IDR-0597555	H_2_N-	4-Br–Ph–SO_2_-	>200 (3)	>100 (3)
**7**	IDR-0596462	2,4-Cl–Ph–NH-	Ph–SO_2_-	>200 (4)	19 ± 9.2 (2)
**8**	IDR-0596461	2,4-Cl–Ph–NH-	Me–SO_2_-	75 (2)	78 ± 29 (2)
**9**	IDR-0597928	2,4-Cl–Ph–NH-	H-	200 (4)	40 ± 2.3 (3)

Upon initial inspection, the sulfonamide functionality appeared potentially labile under the acidic assay conditions. The changes in activity observed across compounds **1**, **7**, and **8** confirmed that the antitubercular activity of **1** was attributable to neither sulfonic acid fragments nor aniline **9** alone. We further confirmed that the intact sulfonamide was required for activity by synthesizing alkyl amine analogs **10–12** and amide analogs **13–14** (Table 2). In all cases, no antitubercular activity was observed.

**Table 2:** 4-Bromophenylsulfonamide substitutions. Compounds were tested for activity against *M. tuberculosis* (MBC_4.5_) and HepG2 cells (IC_50_). MBC_4.5_ are reported as the median. HepG2 IC_50_ are reported as the mean ± standard deviation. The number of replicates is in parentheses

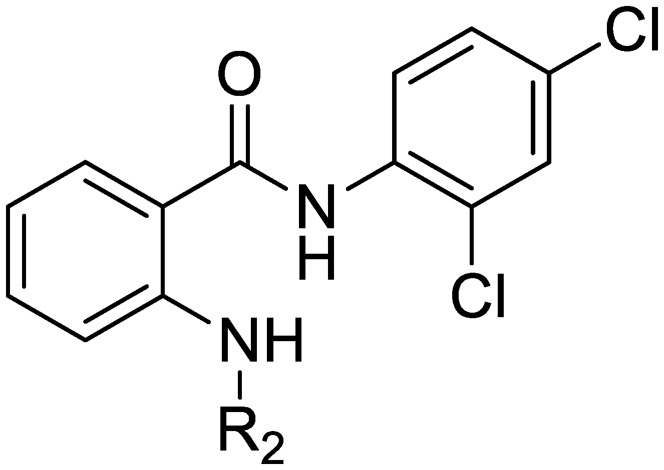				
Cpd	ID	R_2_	MBC_4.5_ (μM)	HepG2 IC_50_ (μM)
**10**	IDR-0596465	Et-	>200 (2)	>100 (2)
**11**	IDR-0597268	Et-, Me-	>200 (2)	69 ± 44 (2)
**12**	IDR-0597556	4-Br–Ph–CH_2_-	>200 (3)	>100 (3)
**13**	IDR-0596464	Ac-	>200 (2)	>100 (2)
**14**	IDR-0596463	4-Br–Ph–CO-	>200 (2)	>100 (2)
**15**	IDR-0597330	4-Me–Ph–SO_2_-	>200 (4)	37 ± 8.5 (2)

During identification of parent compound **1** as the minimum pharmacophore, we observed that *N*-alkyl amides **5** and **6** both demonstrated reduced cytotoxicity despite the loss of antitubercular activity. We hypothesized that replacement of the aniline moiety in **1** with an appropriate alkyl amine might recapture antitubercular activity while maintaining decreased mammalian cytotoxicity. Additional *N*-alkyl amides **16–23** were synthesized to test this hypothesis (Table 3). From these analogs, increased cytotoxicity was observed as the size of the alkyl substituent increased. Only butyl analog **16** showed measurable anti-tubercular activity, albeit with a significant reduction in potency from compound **1**.

**Table 3:** 2,4-Dichloroaniline substitutions. Compounds were tested for activity against *M. tuberculosis* (MBC_4.5_) and HepG2 cells (IC_50_). MBC_4.5_ are reported as the median. HepG2 IC_50_ are reported as the mean ± standard deviation. The number of replicates is in parentheses

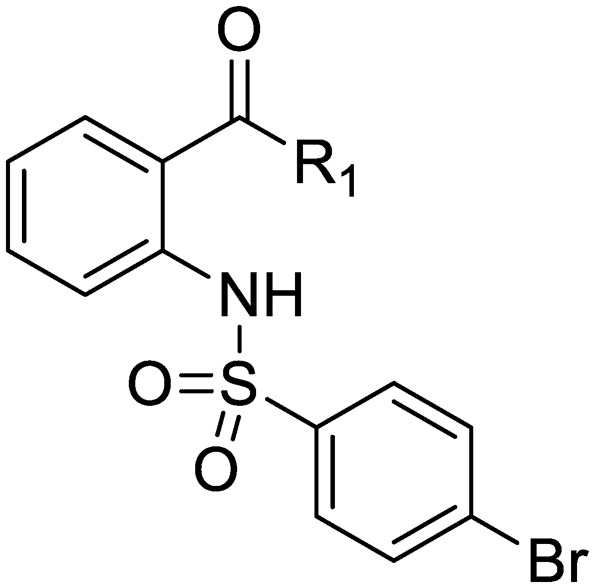				
Cpd	ID	R_1_	MBC_4.5_ (μM)	HepG2 IC_50_ (μM)
**16**	IDR-0600849	BuNH-	100 (4)	59 ± 0.71 (2)
**17**	IDR-0597328	Cyclopropyl-NH-	>200 (2)	>100 (2)
**18**	IDR-0600848	Propargyl-NH-	>200 (5)	83 ± 12 (2)
**19**	IDR-0600851	Cycloheptyl-NH-	>200 (5)	24 ± 6.4 (2)
**20**	IDR-0600850	2-Adamantyl-NH-	>200 (4)	20 ± 4.2 (2)
**21**	IDR-0600833	Piperidyl-	>200 (2)	>100 (5)
**22**	IDR-0600834	Morpholinyl-	>200 (2)	>100 (5)
**23**	IDR-0600866	4-Me-Piperazinyl-	>200 (4)	>100 (2)
**24**	IDR-0597331	2-Pyridyl-NH-	113 (6)	52 ± 13 (2)
**25**	IDR-0597270	2,4-Cl–Ph–NMe-	>200 (2)	60 ± 9.9 (2)

An intramolecular hydrogen bond is likely to exist between the amide oxygen and sulfonamide nitrogen in parent compound **1**, giving rise to a mostly planar core. Several analogs that disrupt this interaction were synthesized, including *N*-methylated amide **25**, 1,3-substituted analog **26**, sulfone **27**, and benzyl analogs **28–29**. In each case, a loss of antitubercular activity pointed to the necessity of the proposed hydrogen-bonded conformer.

Finally, additional alterations were made to the anthranilic amide core (Table 4). The SAR around the core proved restrictive, with cyclohexyl analogs **30–31** and pyridyl analogs **32–33** each resulting in loss of anti-tubercular activity.

**Table 4:** Core substitutions. Compounds were tested for activity against *M. tuberculosis* (MBC_4.5_) and HepG2 cells (IC_50_). MBC_4.5_ are reported as the median. HepG2 IC_50_ are reported as the mean ± standard deviation. The number of replicates is in parentheses (* racemic)

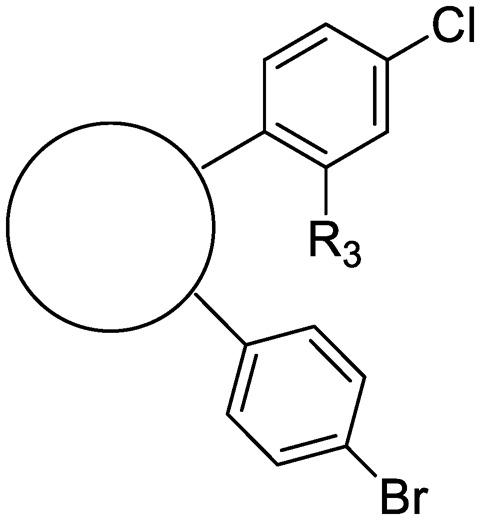					
Cpd	ID	Core	R_3_	MBC_4.5_ (μM)	HepG2 IC_50_ (μM)
**26**	IDR-0600899	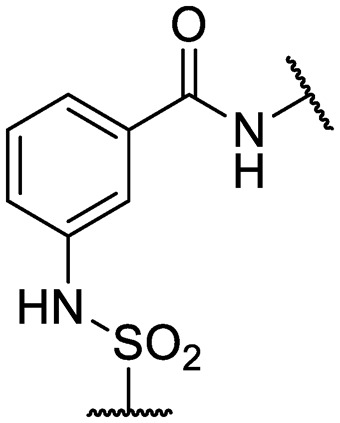	Cl-	>200 (2)	34 ± 3.5 (2)
**27**	IDR-0597462	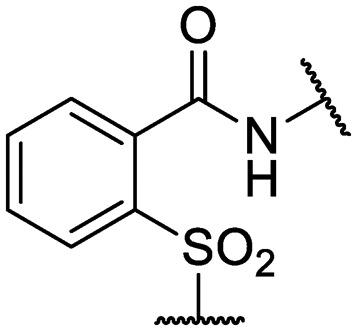	Cl-	>200 (2)	89 ± 18 (3)
**28**	IDR-0597332	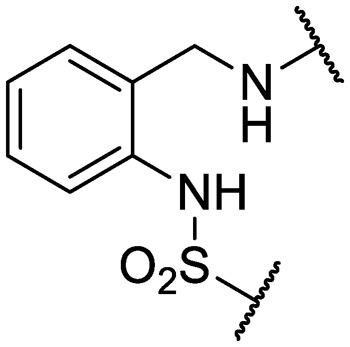	Cl-	>200 (3)	33 ± 0 (2)
**29**	IDR-0597269	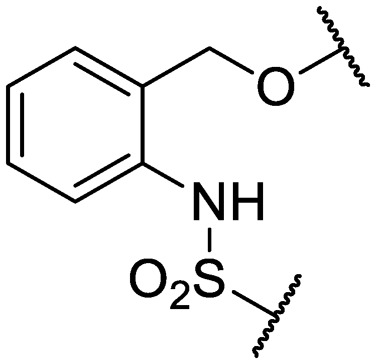	Cl-	>200 (2)	24 ± 6.4 (2)
30*	IDR-0597937	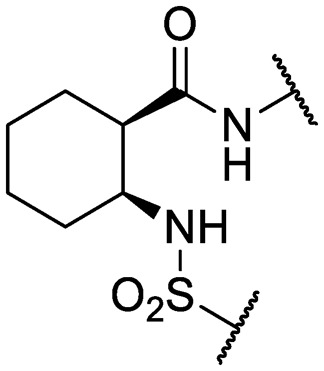	H-	>200 (2)	76 ± 12 (3)
31*	IDR-0597557	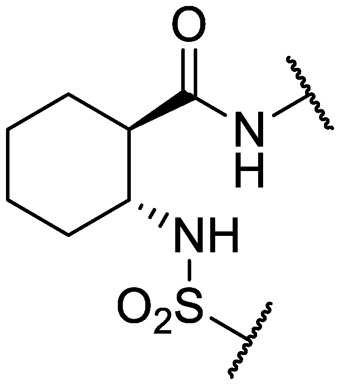	H-	>200 (3)	67 ± 12 (3)
**32**	IDR-0597558	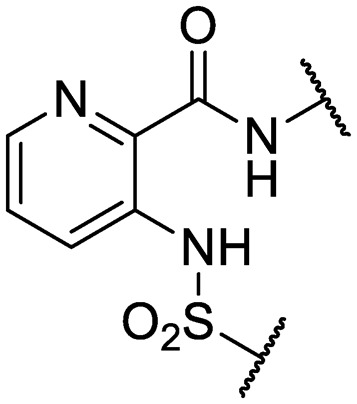	H-	>200 (3)	5.0 ± 1.4 (3)
**33**	IDR-0597589	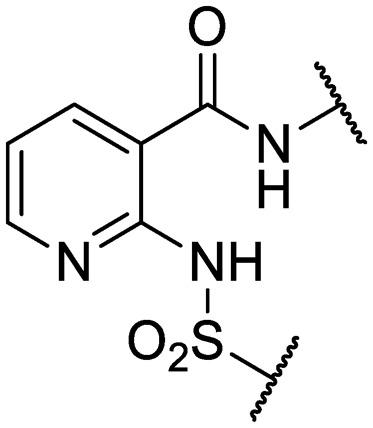	H-	>200 (3)	20 ± 5.7 (2)

The large minimum pharmacophore, preference for halogenated aromatic substituents, and resistance to change from a non-polar, planar core all present challenges for further development of compound **1** as an effective chemotherapeutic. Considered alongside the generally observed penchant for mammalian cytotoxicity, exploration of the anthranilic amide SAR led us away from consideration of the scaffold.

Exploration of the structure–activity relationship around screening hit **2** was more limited. We began by synthesizing desnitro analog **34** and, upon observing retention of antitubercular activity, assayed a loosely related set of compounds, the majority of which were inactive. For brevity's sake, Table 5 reports only the active analogs, on which all follow up studies were performed.

**Table 5:** Benzothiadiazole series. Compounds were tested for activity against *M. tuberculosis* (MBC_4.5_) and HepG2 cells (IC_50_). MBC_4.5_ are reported as the median. HepG2 IC_50_ are reported as the mean ± standard deviation. The number of replicates is in parentheses

Cpd	ID	Structure	MBC_4.5_ (μM)	HepG2 IC_50_ (μM)
**2**	IDR-0099118	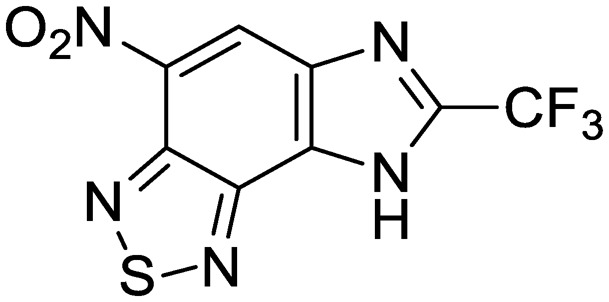	25 (2)	83 ± 24 (8)
**34**	IDR-0107334	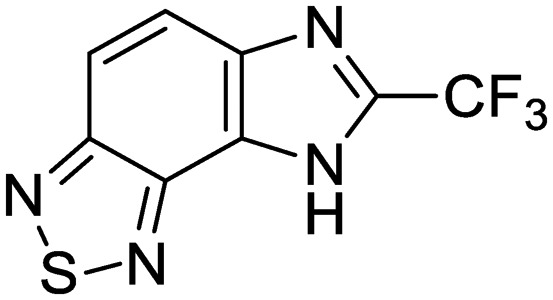	75 (2)	70 ± 11 (2)
**35**	IDR-0697786	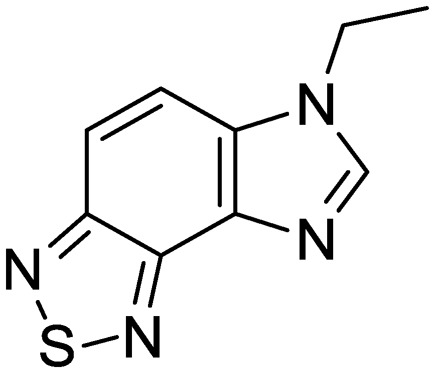	75 (2)	>100 (2)
**36**	IDR-0697784	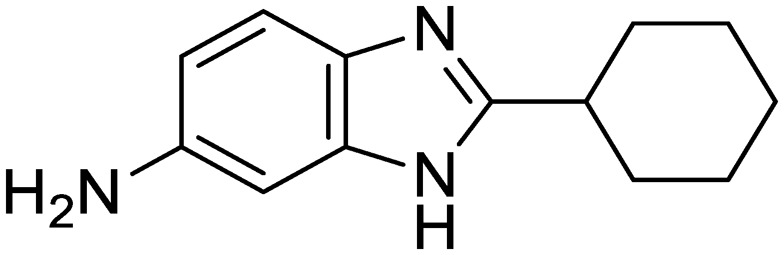	100 (2)	>100 (2)
**37**	IDR-0050636	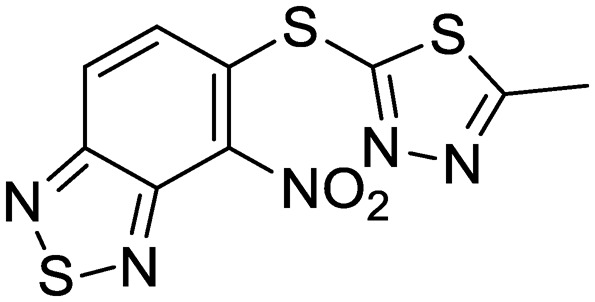	19 (2)	5.6 ± 3.2 (5)
**38**	IDR-0033566	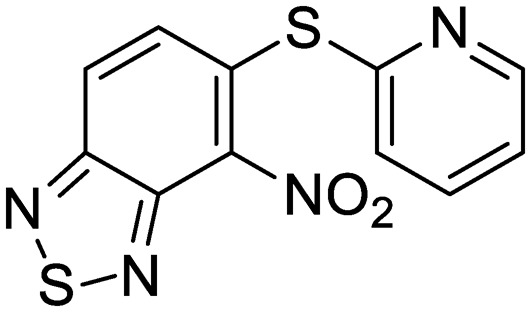	25 (2)	4.8 ± 0.86 (3)

In addition to determination of the MBC_4.5_ for each compound, we evaluated antitubercular activity of the compounds as measured by MIC at pH 5.6 (Table S1†) and IC_50_ against intracellular *M. tuberculosis* in RAW 264.7 cells (ATCC TIB-71) (Table S2†). While many compounds proved active extracellularly, no correlation was observed between MIC_5.6_ and MBC_4.5_. Intracellularly, only compound **11** showed antitubercular activity. General cytotoxicity against the infected RAW 264.7 cells was observed and was correlated with cytotoxicity against HepG2 cells (Table S2†).

**Fig. 3:**
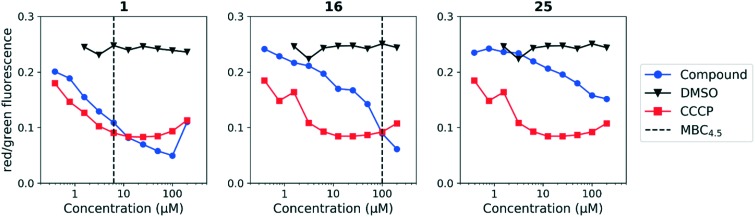
Disruption of *M. tuberculosis* membrane potential by anthranilic amide compounds at pH 6.8. Compounds were tested for their ability to disrupt membrane potential at pH 6.8 in *M. tuberculosis*. DMSO (negative control) and CCCP (positive control) were included. Reported values are a representative from two independent runs. MBC_4.5_ is reported as the median of two or more replicates.

We sought to rule out non-specific activity as the primary driver of biological activity. We tested the effect of a selection of compounds from both series on the membrane potential in *M. tuberculosis*. For the anthranilic amides, we tested compounds with a range of activity (Fig. 3). The level of disruption of membrane potential was directly correlated with the MBC_4.5_. The most active compound (**1**) depolarized the membrane nearly as strongly as known uncoupler carbonyl cyanide *m*-chlorophenyl hydrazone (CCCP).^36^ The inactive compound (**25**) also showed some capacity to depolarize the membrane, but the effect was at much higher concentrations. For all three compounds, the effect on membrane potential was observed at concentrations below the MBC_4.5_. This behavior suggests that bacterial death is the result of a loss of polarization, rather than the disruption being a result of cell death.

**Fig. 4:**
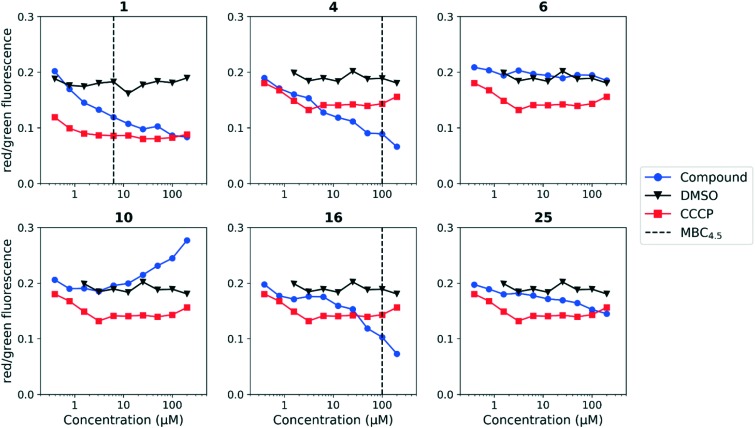
Disruption of *M. tuberculosis* membrane potential by anthranilic amide compounds at pH 5.6. Compounds were tested for their ability to disrupt membrane potential at pH 5.6 in *M. tuberculosis*. DMSO (negative control) and CCCP (positive control) were included. Reported values are a representative from two independent runs. MBC_4.5_ is reported as the median of two or more replicates.

Since the anthranilic amide compound series originated from a pH-dependent screen, we hypothesized that the compounds would have greater activity on membrane potential at lower pH. We tested compounds at pH 5.6; however, the effect on membrane potential was generally unchanged from pH 6.8. Of those tested, only compound **16** resulted in disruption at significantly lower concentrations relative to pH 6.8 (Fig. 4). Compounds **1**, **4**, and **16** maintained comparable disruption to CCCP at concentrations one or more orders of magnitude below their MBC_4.5_.

Compound **25**, inactive by MBC_4.5_, also maintained its disruption of *M. tuberculosis* membrane potential at pH 5.6. Given the lack of observed antitubercular activity, it is possible that the depolarization caused by compound **25** is the result of an alternative mechanism than that of the other tested compounds. As expected, compound **6**, which lacked antitubercular activity, did not disrupt *M. tuberculosis* membrane potential. Interestingly, compound **10**, which also lacked antitubercular activity, caused membrane hyperpolarization in *M. tuberculosis* at pH 5.6. While we were unable to find evidence that the membrane potential disruptive effect was pH-dependent, considered as a whole, these results lead us to conclude that membrane potential disruption is directly associated with antitubercular activity for the anthranilic amide family of compounds.

An attempt was made to adapt the assay to run at pH 4.5, matching the conditions under which the MBC_4.5_ was determined; however, the window between the positive and negative controls collapsed under these conditions (Fig. S1 and S2†). We believe this collapse to be the result of *M. tuberculosis* membrane potential naturally decreasing in acidic media as previously reported by Zhang *et al.*^35^

**Fig. 5:**
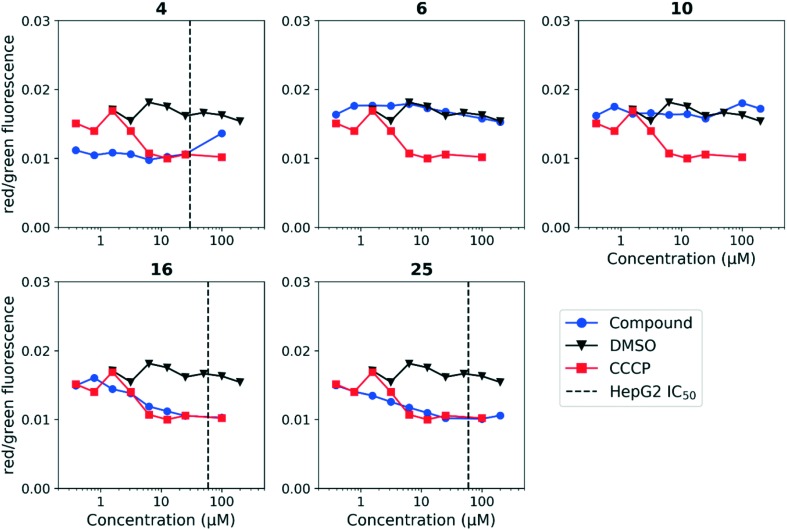
Disruption of HepG2 cell membrane potential by anthranilic amide compounds. Compounds were tested for their ability to disrupt membrane potential in HepG2 cells. DMSO (negative control) and CCCP (positive control) were included. Values are a representative from two independent runs. HepG2 IC_50_ is reported as the mean of two or more replicates.

Having established a correlation between antitubercular activity and cytotoxicity during our SAR studies, we hypothesized that the anthranilic amide series may be similarly disrupting mitochondrial membrane potential in mammalian cells. We tested this using HepG2 cells and found the same trends as observed in *M. tuberculosis* (Fig. 5). The cytotoxic compounds (**4**, **16**, **25**) generated a decrease in membrane potential in HepG2 cells comparable in magnitude to CCCP at concentrations significantly below the IC_50_. Non-cytotoxic compounds (**6**, **10**) did not disrupt membrane potential at concentrations up to 200 μM. As with the evaluation of antitubercular activity, the relationship between the concentrations of compound required to produce cytotoxicity and to depolarize HepG2 cells led us to conclude that the two effects were directly associated.

**Fig. 6:**
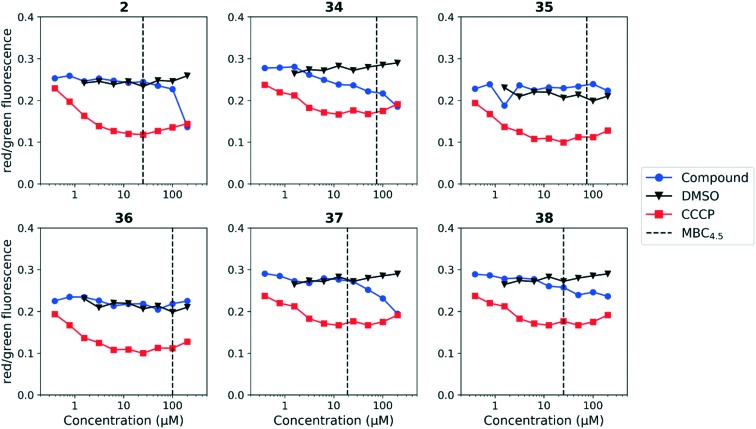
Disruption of *M. tuberculosis* membrane potential by benzothiadiazole compounds at pH 6.8. Compounds were tested for their ability to disrupt membrane potential at pH 6.8 in *M. tuberculosis*. DMSO (negative control) and CCCP (positive control) were included. Values are a representative from two independent runs. MBC_4.5_ is reported as the median of two or more replicates.

**Fig. 7:**
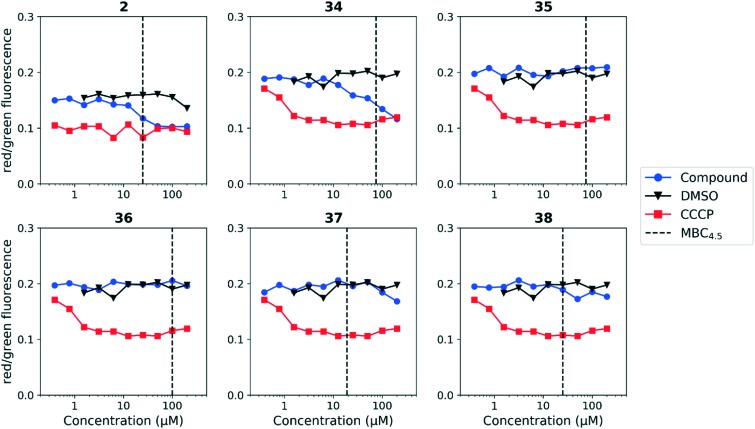
Disruption of *M. tuberculosis* membrane potential by benzothiadiazole compounds at pH 5.6. Compounds were tested for their ability to disrupt membrane potential at pH 5.6 in *M. tuberculosis*. DMSO (negative control) and CCCP (positive control) were included. Reported values are a representative from two independent runs. MBC_4.5_ is reported as the median of two or more replicates.

**Fig. 8:**
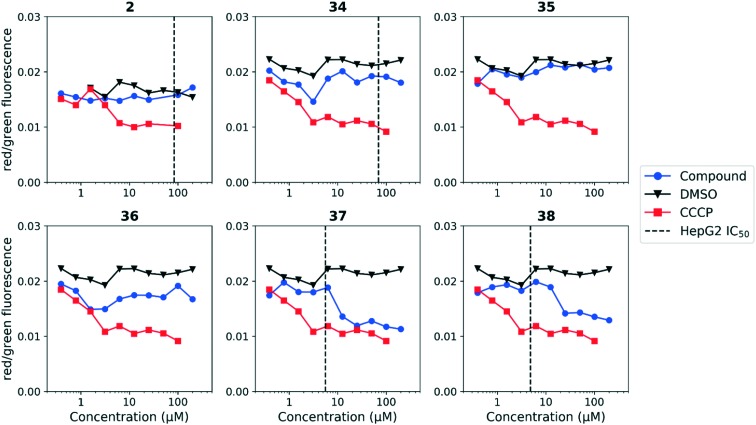
Disruption of HepG2 cell membrane potential by benzothiadiazole compounds. Compounds were tested for their ability to disrupt membrane potential in HepG2 cells. DMSO (negative control) and CCCP (positive control) were included. Values are representative from two independent runs. HepG2 IC_50_ is reported as the mean of two or more replicates.

We tested whether benzothiadiazole compounds disrupted *M. tuberculosis* membrane potential at neutral pH (Fig. 6). Although the compounds disrupted membrane potential, the majority did so at concentrations above the MBC_4.5_, and there was little enhancement of this activity at pH 5.6 (Fig. 7). In addition, the benzothiadiazole compounds showed no correlation between disruption of membrane potential and HepG2 IC_50_, suggesting that the drop in membrane potential that was observed for compounds **37** and **38** was the result (not the cause) of cell death (Fig. 8). Taken altogether, we concluded that disruption of membrane potential was neither the driver of antitubercular activity nor cytotoxicity for the benzothiadiazole family of compounds.

## Conclusions

We identified and characterized SAR for the anthranilic amide and benzothiadiazole series. The contrasting activity profiles for these two compound series highlights the need for interpretation of membrane potential disruption within the context of antitubercular activity. While the anthranilic acid series demonstrated a clear relationship between the bactericidal effect and the onset of membrane depolarization, the benzothiadiazole series showed no such correlation. Thus the latter may affect membrane potential as a secondary effect. This difference suggests differing underlying mechanisms of action for the two series, the specifics of which might be revealed through more targeted studies.

The disruption of membrane potential was correlated with both antitubercular activity and cytotoxicity for the anthranilic acid family. As a result, disentanglement of the two activities with additional medicinal chemistry efforts is unlikely. Despite this, compounds such as **16** may serve as useful tool compounds in the evaluation of *M. tuberculosis* membrane potential disruption, particularly at pH 5.6, where it provides a larger, more consistent degree of separation from DMSO than CCCP.

The outlook for the benzothiadiazole family as a therapeutic is more promising, with disruption of membrane potential ruled out as the direct agent of both antitubercular activity and cytotoxicity. Presenting a potentially novel mechanism of action, compound **2** projects an attractive target for more focused SAR exploration.

## Experimental

**Scheme 1:**
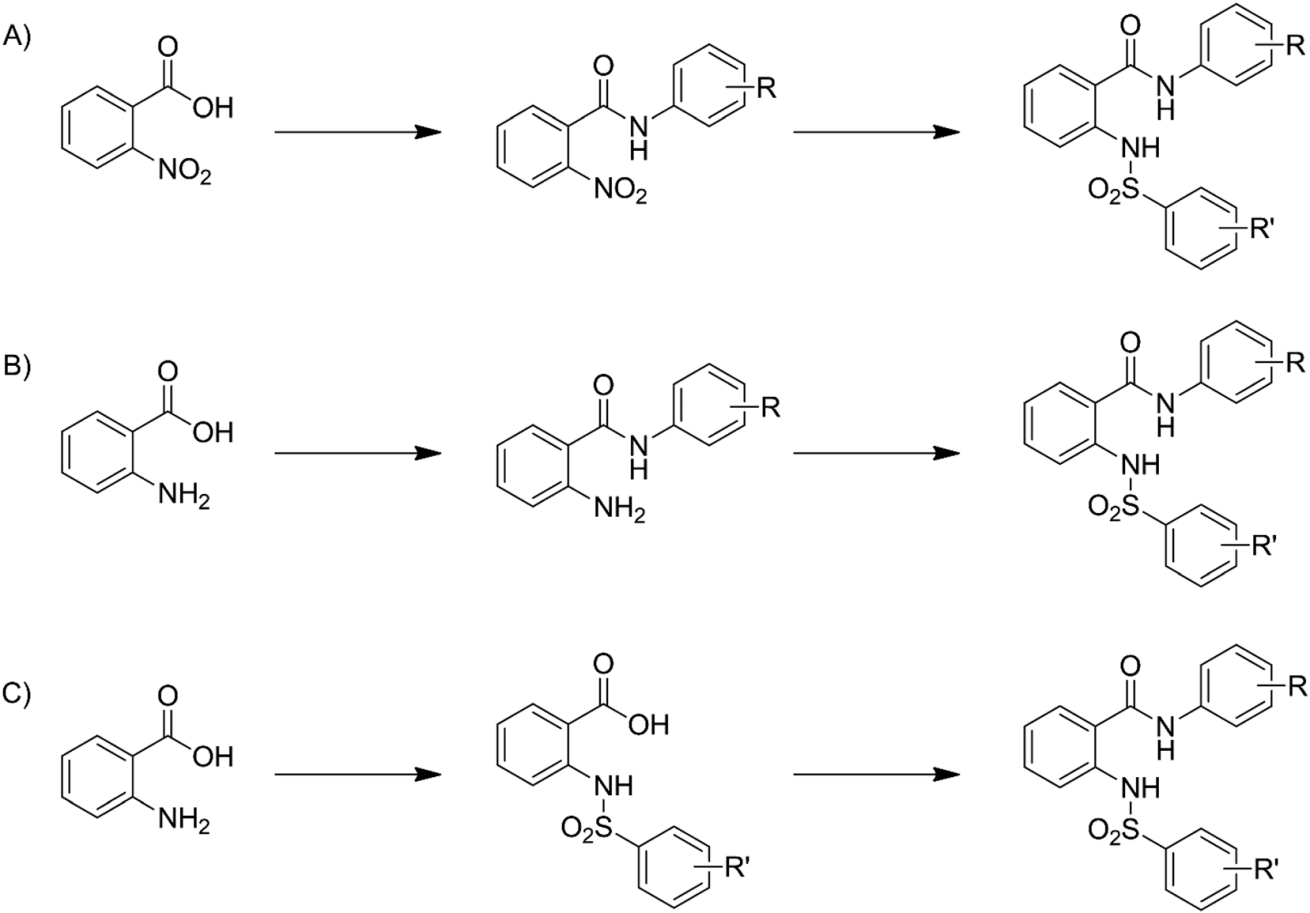
Generalized synthetic routes A, B, and C to anthranilic amide compounds.

Compounds **3–33** were synthesized *via* general routes A, B, or C (Scheme 1) then characterized by ^1^H NMR and LC/MS. Complete synthetic methods may be found in the ESI.† Compounds **1–2** and **34–38** were the generous gift of Eli Lilly and Company.

Cultures: *M. tuberculosis* H37Rv was cultured in Middlebrook 7H9 medium supplemented with 0.05% w/v Tween 80 and 10% v/v oleic acid, albumin, dextrose and catalase supplement (OADC; Becton Dickinson) (7H9-Tw-OADC).

A recombinant strain of *M. tuberculosis* H37Rv carrying plasmid pMV306hsp+LuxAB+G13+CDE (which constitutively expresses luciferase)^37^ was cultured in Middlebrook 7H9 medium containing 10% v/v OADC (oleic acid, albumin, dextrose, catalase) (Becton Dickinson) and 0.05% v/v Tween 80 (7H9-OADC-Tw) plus 20 μg mL^–1^ kanamycin (Sigma-Aldrich) at 37 °C.^37^,^38^

Measurement of membrane potential was adapted from the method published by Eoh and Rhee.^39^ Briefly, *M. tuberculosis* cultures were grown in 7H9-OADC-Tw to mid-logarithmic phase and concentrated to an OD_590_ ∼ 1.0 in fresh 7H9-Tw (adjusted to the indicated pH). Cells were incubated with 15 μM DiOC_2_ at room temperature for 20 minutes. Mtb cultures were washed in and added to black-walled 96-well plates (Grenier) containing 2-fold serial dilution of test compounds and incubated for 15 min at RT. The protonophore carbonyl-cyanide 3-chlorophenylhydrazone (CCCP) (Sigma) was used as a positive control for membrane depolarization. DMSO served as a vehicle control. A Synergy H4 microplate reader (BioTek Instruments) was used to measure green fluorescence (488 nm/530 nm) and shifts to red fluorescence (488 nm/650 nm). Membrane potential was measured as a ratio of red to green fluorescence.

Measurement of membrane potential in HepG2 human liver cells (ATCC) was adapted from the method published by Huang.^40^ Cells were plated at a density of 50 000 cells per well (100 μL) in black-walled 96-well plates and incubated overnight. Culture medium was replaced with the same volume of assay buffer (80 mM NaCl, 75 mM KCl, 25 mM d-glucose, 25 mM HEPES pH 7.4) containing test compounds at indicated concentrations; CCCP and DMSO served as controls. Plates were incubated at 37 °C for 30 min, followed by incubation with 4 μM DiOC_2_ for 20 min at RT. Cells were washed 4 times with 150 μL assay buffer, and suspended in 100 μL of assay buffer. A Synergy H1 microplate reader (BioTek Instruments) was used to measure fluorescence (488 nm/530 nm and 488 nm/620 nm). Each test compound was evaluated in at least two replicate experiments.

Determination of Minimum Bactericidal Concentration at pH 4.5 (MBC_4.5_): *M. tuberculosis* H37Rv constitutively expressing the entire luciferase cassette was incubated in the presence of compound in phosphate citrate buffer at pH 4.5 for 7 days at 37 °C starting at >10^8^ CFU mL^–1^. Compounds were tested as 2-fold serial dilutions, typically starting at 200 μM, and the minimum concentration required to drop the RLU below the threshold value, which correlated with a 2-log reduction, was reported as the MBC_4.5_.

Determination of minimum inhibitory concentrations (MIC): minimum inhibitory concentrations (MICs) were determined in liquid medium as described previously.^41^ Briefly, compounds were solubilized in DMSO and assayed as 10-point 2-fold serial dilution series. Bacterial growth was measured in Middlebrook 7H9-OADC medium with 0.05% tyloxapol adjusted to either pH 5.6 or 6.8. For pH 5.6 the starting OD_590_ was 0.04 and plates were incubated for 6 days at 37 °C, while for pH 6.8 the starting OD_590_ was 0.02 and plates were incubated for 5 days. MICs were determined by measuring growth by OD_590_ and three parameter nonlinear fit. Each experiment had two independent replicates.

Non-replicating kill kinetics at pH 5.5: Late logarithmic phase H37Rv (OD590 0.6–1.0) was harvested and resuspended in phosphate citrate buffer plus 0.05% tyloxapol. Cultures were inoculated to ∼10^6^ CFU mL^–1^, compounds added (final concentration 2% DMSO), and incubated standing at 37 °C. Aliquots were plated for CFU every 7 days. Plates were incubated at 37 °C for 4 weeks before counting.

Cytotoxicity was evaluated against the HepG2 cell line (ATCC) as described previously.^42^ Briefly, HepG2 cells were propagated in medium containing either glucose or galactose: DMEM (Invitrogen), 10% FBS, 1 mM sodium pyruvate, 2 mM glutagro (Corning), 100 I.U mL^–1^ penicillin and 100 μg mL^–1^ streptomycin (Corning), 25 mM glucose or 10 mM d-galactose (Sigma). Cells were seeded in 384-well plates at 1800 cells per well and incubated in a humidified incubator at 37 °C, 5% CO_2_. Compounds were solubilized in 100% DMSO and assayed using a 10-point three-fold serial dilution. Compounds were added 24 hours post cell seeding to a final assay concentration of 1% DMSO and highest compound concentration of 100 μM. CellTiter-Glo® reagent (Promega) was added to 384-well plates after a 72 hour incubation period. Relative luminescent units (RLU) were measured using a Synergy H1 plate reader (Biotek). Raw data were normalized using the average RLU value from negative control (1% DMSO) and expressed as percentage growth. Growth inhibition curves were fitted using the Levenberg–Marquardt algorithm. The IC_50_ was defined as the compound concentration that produced 50% of the growth inhibitory response.

## Conflicts of interest

The authors declare no conflicts of interest.

## Supplementary Material

Supplementary information
