# Supplementary material for: Anthranilic amide and imidazobenzothiadiazole compounds disrupt Mycobacterium tuberculosis membrane potential
Source: Medchemcomm. 2019 May 3;10(6):934–45. doi: 10.1039/c9md00088g (PMC6596218; doi:10.1039/c9md00088g)

Electronic Supplementary Material (ESI) for MedChemComm.
This journal is © The Royal Society of Chemistry 2019

Supporting Information

Anthranilic Amide and Imidazobenzothiadiazole Compounds Disrupt Mycobacterium tuberculosis Membrane Potential: Relationship to Bactericidal Activity and Mammalian Cytotoxicity

Jake Smith[a], Heather Wescott[a], Julie Early[a], Steven Mullen[a], Junitta Guzman[a], Joshua Odingo[a], Jason Lamar[b], Tanya Parish[a]*

[a] TB Discovery Research, Infectious Disease Research Institute, 1616 Eastlake Ave E, Suite 400, Seattle, Washington 98102, United States

[b] Lilly Research Laboratories, Eli Lilly and Company, 307 E Merrill St, Indianapolis, Indiana 46285, United States

* Corresponding Author

Contents

1. *M. tuberculosis* Membrane Potential - pH 4.5 4

2. *M. tuberculosis* Minimum Inhibitory Concentrations 6

3. Intracellular *M. tuberculosis* Minimum Inhibitory Concentrations and RAW 264.7 Cytotoxicity 7

4. Chemical Methods 8

General Methods 8

Abbreviations 8

N-(2,4-dichlorophenyl)-2-nitrobenzamide (S1) 8

2-amino-N-(2,4-dichlorophenyl)benzamide (9) 9

2-amino-N-(4-chlorophenyl)benzamide (S2) 9

2-(4-bromophenylsulfonamido)-N-(4-chlorophenyl)benzamide (3) 9

2-nitro-N-phenylbenzamide (S3) 10

2-amino-N-phenylbenzamide (S4) 10

2-(4-bromophenylsulfonamido)-N-phenylbenzamide (4) 11

2-(4-bromophenylsulfonamido)benzoic acid (S5) 11

2-(4-bromophenylsulfonamido)-N-ethylbenzamide (5) 11

2-(4-bromophenylsulfonamido)benzamide (6) 12

N-(2,4-dichlorophenyl)-2-(phenylsulfonamido)benzamide (7) 12

N-(2,4-dichlorophenyl)-2-(methylsulfonamido)benzamide (8) 13

N-(2,4-dichlorophenyl)-2-(ethylamino)benzamide (10) 13

N-(2,4-dichlorophenyl)-2-(methylamino)benzamide (S6) 14

N-(2,4-dichlorophenyl)-2-(ethyl(methyl)amino)benzamide (11) 14

2-((4-bromobenzyl)amino)-N-(2,4-dichlorophenyl)benzamide (12) 15

2-acetamido-N-(2,4-dichlorophenyl)benzamide (13) 15

2-(4-bromobenzamido)-N-(2,4-dichlorophenyl)benzamide (14) 16

N-(2,4-dichlorophenyl)-2-(4-methylphenylsulfonamido)benzamide (15) 16

2-(4-bromophenylsulfonamido)-N-butylbenzamide (16) 17

2-(4-bromophenylsulfonamido)-N-cyclopropylbenzamide (17) 17

2-(4-bromophenylsulfonamido)-N-(pro-2-yn-1-yl)benzamide (18) 18

2-(4-bromophenylsulfonamido)-N-cycloheptylbenzamide (19) 18

N-(adamantan-2-yl)-2-(4-bromophenylsulfonamido)benzamide (20) 19

4-bromo-N-(2-(piperidine-1-carbonyl)phenyl)benzenesulfonamide (21) 19

4-bromo-N-(2-(morpholine-4-carbonyl)phenyl)benzenesulfonamide (22) 20
4-bromo-N-(2-(4-methylpiperizine-1-carbonyl)phenyl)benzenesulfonamide (23) 20
2-nitro-N-(pyridin-2-yl)benzamide (S7) 21
2-amino-N-(2-pyridyl)benzamide (S8) 21
2-(4-bromophenylsulfonamido)-N-(pyridin-2-yl)benzamide (24) 21
N-(2,4-dichlorophenyl)-N-methyl-2-nitrobenzamide (S9) 22
2-amino-N-(2,4-dichlorophenyl)-N-methylbenzamide (S10) 22
2-(4-bromophenylsulfonamido)-N-(2,4-dichlorophenyl)-N-methylbenzamide (25) 23
3-(4-bromophenylsulfonamido)benzoic acid (S11) 23
3-(4-bromophenylsulfonamido)-N-(2,4-dichlorophenyl)benzamide (26) 24
N-(2,4-dichlorophenyl)-2-iodobenzamide (S12) 24
2-((4-bromophenyl)sulfonyl)-N-(2,4-dichlorophenyl)benzamide (27) 25
N-(2-aminobenzyl)-2,4-dichloroaniline (S13) 25
4-bromo-N-(2-(((2,4-dichlorophenyl)amino)methyl)phenyl)benzenesulfonamide (28) 26
2-((3,4-dichlorophenoxy)methyl)aniline (S14) 26
4-bromo-N-(2-((2,4-dichlorophenoxy)methyl)phenyl)benzenesulfonamide (29) 27
cis-2-(4-bromophenylsulfonamido)-cyclohexanecarboxylic acid (S15) 27
cis-2-(4-bromophenylsulfonamido)-N-(4-chlorophenyl)cyclohexanecarboxamide (30) 28
trans-2-(4-bromophenylsulfonamido)-cyclohexanecarboxylic acid (S16) 28
trans-2-(4-bromophenylsulfonamido)-N-(4-chlorophenyl)cyclohexanecarboxamide (31) 29
3-amino-N-(4-chlorophenyl)picolinamide (S17) 29
3-(4-bromophenylsulfonamido)-N-(4-chlorophenyl)picolinamide (32) 30
2-amino-N-(4-chlorophenyl)nicotinamide (S18) 30
2-(4-bromophenylsulfonamido)-N-(4-chlorophenyl)nicotinamide (33) 31
5. NMR Spectra of Final Compounds 32

1. *M. tuberculosis* Membrane Potential - pH 4.5

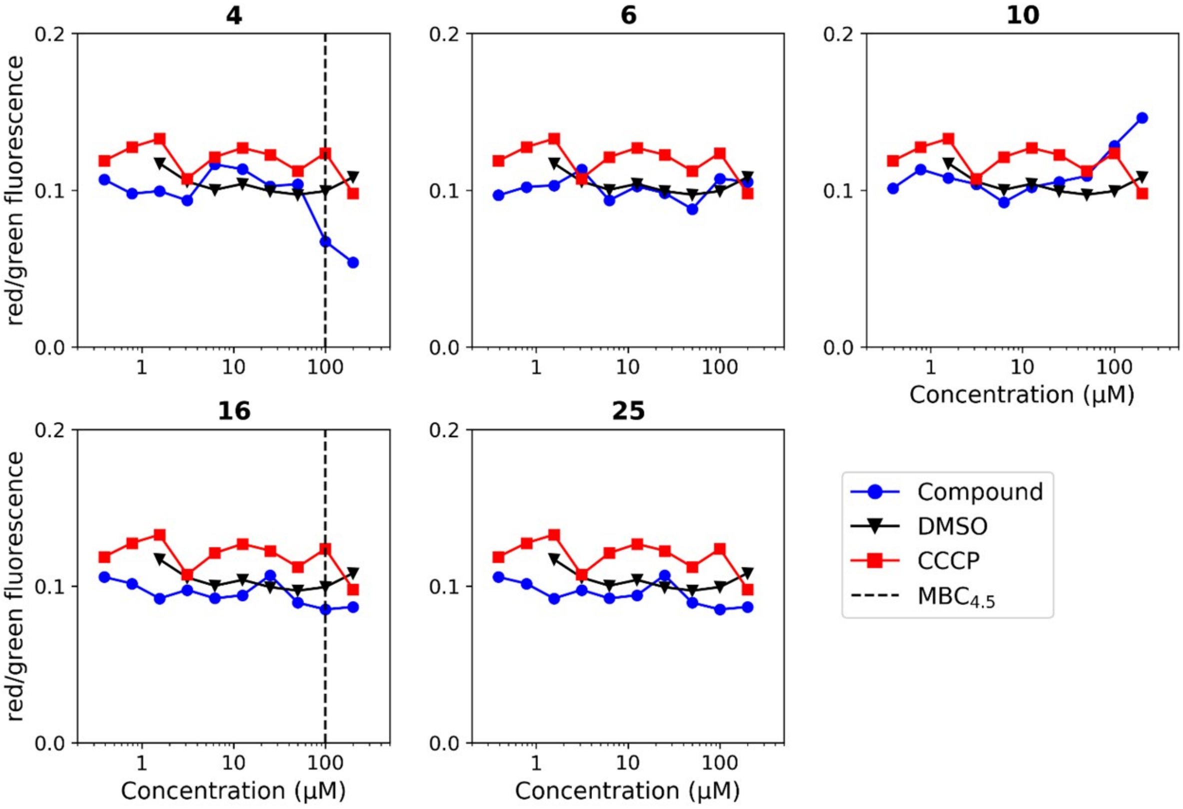

Figure S1. **Disruption of *M. tuberculosis* membrane potential by anthranilic amide compounds at pH 4.5.** Compounds were tested for their ability to disrupt membrane potential at pH 4.5 in *M. tuberculosis*. DMSO (negative control) and CCCP (positive control) were included. Reported values are a representative from two independent runs. $MBC_{4.5}$ is reported as the median of two or more replicates.

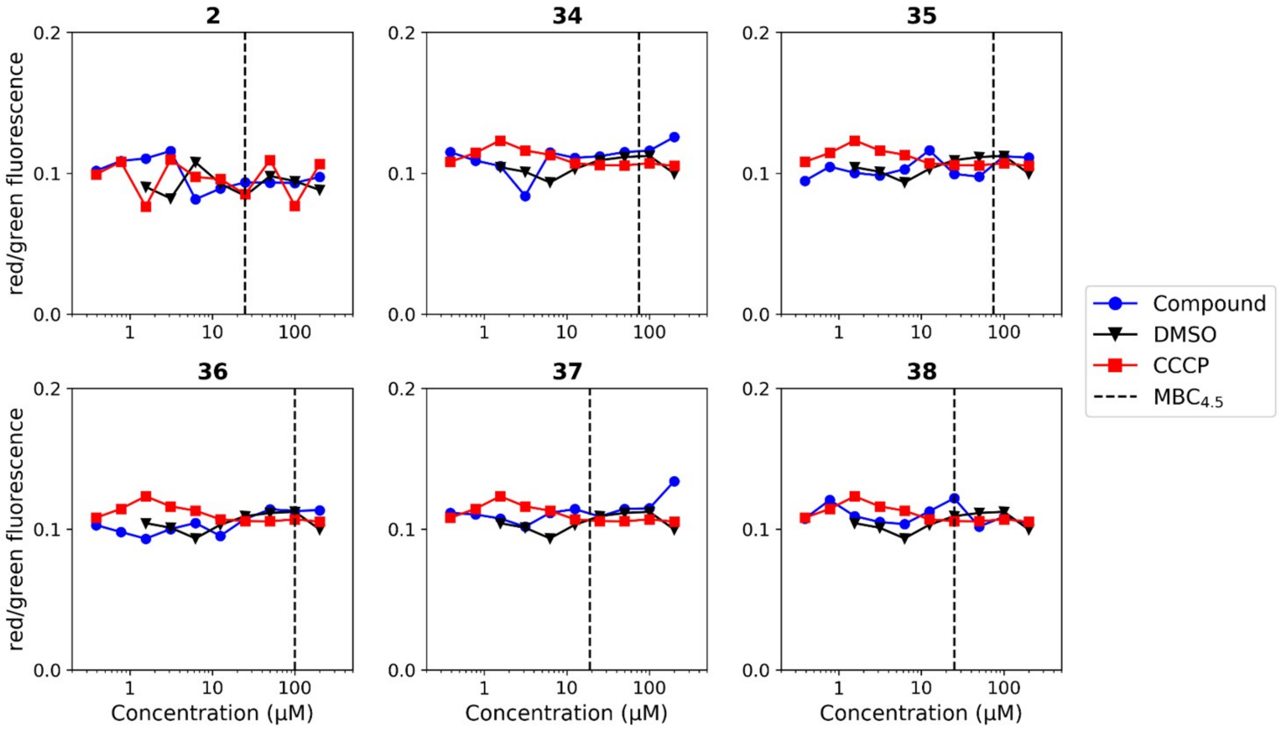

Figure S2. **Disruption of *M. tuberculosis* Membrane potential by benzothiadiazole compounds at pH 4.5.** Compounds were tested for their ability to disrupt membrane potential at pH 4.5 in *M. tuberculosis*. DMSO (negative control) and CCCP (positive control) were included. Reported values are a representative from two independent runs. $MBC_{4.5}$ is reported as the median of two or more replicates.

2. *M. tuberculosis* Minimum Inhibitory Concentrations

Cpd	ID	MIC pH 5.6 (µM)	MIC pH 6.8 (µM)
**1**	IDR-0019306	20 ± 0 (2)	182 ± 35 (4)
**2**	IDR-0099118	115 ± 15 (2)	>200 (2)
**3**	IDR-0597329	26 ± 5.7 (2)	110 ± 0 (2)
**4**	IDR-0484542	63 ± 3.5 (2)	185 ± 21 (2)
**5**	IDR-0597554	78 ± 4.2 (2)	>200 (5)
**6**	IDR-0597555	71 ± 12 (2)	>200 (4)
**7**	IDR-0596462	19 ± 0.71 (2)	>200 (2)
**8**	IDR-0596461	46 ± 7.1 (2)	>200 (1)
**9**	IDR-0597928	>200 (2)	>200 (3)
**10**	IDR-0596465	85 ± 35 (2)	175 ± 35 (2)
**11**	IDR-0597268	115 ± 21 (2)	115 ± 7.1 (2)
**12**	IDR-0597556	>200 (2)	>200 (5)
**13**	IDR-0596464	>200 (2)	>200 (2)
**14**	IDR-0596463	>200 (2)	>200 (2)
**15**	IDR-0597330	19 ± 12 (2)	>200 (2)
**16**	IDR-0600849	136 ± 91 (2)	>200 (3)
**17**	IDR-0597328	96 ± 20 (2)	>200 (2)
**18**	IDR-0600848	77 ± 9.9 (2)	>200 (3)
**19**	IDR-0600851	106 ± 91 (2)	>200 (3)

Cpd	ID	MIC pH 5.6 (µM)	MIC pH 6.8 (µM)
**20**	IDR-0600850	>200 (2)	>200 (2)
**21**	IDR-0600833	170 ± 42 (2)	>200 (2)
**22**	IDR-0600834	>200 (2)	>200 (2)
**23**	IDR-0600866	>200 (2)	>200 (3)
**24**	IDR-0597331	44 ± 32 (3)	>200 (2)
**25**	IDR-0597270	154 ± 72 (3)	>200 (2)
**26**	IDR-0600899	95 ± 7.8 (2)	>200 (2)
**27**	IDR-0597462	>200 (2)	>200 (2)
**28**	IDR-0597332	>200 (4)	>200 (3)
**29**	IDR-0597269	112 ± 78 (3)	170 ± 42 (2)
**30**	IDR-0597937	>200 (2)	>200 (3)
**31**	IDR-0597557	>200 (5)	>200 (2)
**32**	IDR-0597558	120 ± 113 (2)	53 ± 11 (5)
**33**	IDR-0597589	>200 (2)	>200 (5)
**34**	IDR-0107334	145 ± 7.1 (2)	>200 (2)
**35**	IDR-0697786	>200 (2)	>200 (2)
**36**	IDR-0697784	>200 (2)	>200 (2)
**37**	IDR-0050636	4.2 ± 0.21 (2)	30.5 ± 0.71 (2)
**38**	IDR-0033566	8.1 ± 0.071 (2)	24 ± 2.6 (2)

Table S1. **Minimum Inhibitory Concentrations.** Compounds were tested for inhibitory activity against *M. tuberculosis* at pH 5.6 and pH 6.8. MIC are reported as the mean ± standard deviation. The number of replicates is in parentheses.

3. Intracellular *M. tuberculosis* Minimum Inhibitory Concentrations and RAW 264.7 Cytotoxicity

Cpd	ID	Intracellular IC_{50} (μM)	RAW IC_{50} (μM)	Cpd	ID	Intracellular IC_{50} (μM)	RAW IC_{50} (μM)
**1**	IDR-0019306	>11 (6)	2.8 ± 0.68 (6)	**20**	IDR-0600850	>11 (2)	12 ± 2.8 (2)
**2**	IDR-0099118	>33 (4)	35 ± 14 (4)	**21**	IDR-0600833	>100 (4)	73 ± 30 (4)
**3**	IDR-0597329	>3.7 (1)	7.0 ± 3.3 (2)	**22**	IDR-0600834	>100 (4)	>100 (4)
**4**	IDR-0484542	>11 (1)	13 (1)	**23**	IDR-0600866	>100 (2)	>100 (2)
**5**	IDR-0597554	>100 (3)	85 ± 17 (3)	**24**	IDR-0597331	>11 (2)	29 ± 0 (2)
**6**	IDR-0597555	>100 (3)	>100 (3)	**25**	IDR-0597270	>100 (3)	30 ± 0.58 (3)
**7**	IDR-0596462	>3.7 (2)	9.8 ± 0.28 (2)	**26**	IDR-0600899	>11 (2)	14 ± 4.9 (2)
**8**	IDR-0596461	>33 (2)	47 ± 6.4 (2)	**27**	IDR-0597462	>33 (4)	53 ± 8.5 (4)
**9**	IDR-0597928	>33 (6)	49 ± 36 (6)	**28**	IDR-0597332	>3.7 (2)	9.9 ± 0.14 (2)
**10**	IDR-0596465	>100 (2)	61 ± 40 (2)	**29**	IDR-0597269	8.7 (1)	11 ± 0.69 (3)
**11**	IDR-0597268	3.9 ± 0.53 (3)	22 ± 9.2 (3)	**30**	IDR-0597937	>100 (5)	82 ± 17 (5)
**12**	IDR-0597556	>100 (3)	>100 (3)	**31**	IDR-0597557	>100 (3)	30 ± 2.5 (3)
**13**	IDR-0596464	>100 (2)	>100 (2)	**32**	IDR-0597558	>1.2 (3)	1.0 ± 0.092 (3)
**14**	IDR-0596463	>100 (2)	98 ± 2.8 (2)	**33**	IDR-0597589	>11 (2)	10 ± 0 (2)
**15**	IDR-0597330	>11 (1)	11 ± 0 (2)	**34**	IDR-0107334	>100 (3)	92 ± 2.3 (3)
**16**	IDR-0600849	>33 (2)	30 ± 1.4 (2)	**35**	IDR-0697786	>100 (4)	>100 (4)
**17**	IDR-0597328	>100 (2)	>100 (2)	**36**	IDR-0697784	>100 (4)	>100 (4)
**18**	IDR-0600848	>100 (2)	94 ± 9.2 (2)	**37**	IDR-0050636	>3.7 (3)	2.9 ± 0.68 (3)
**19**	IDR-0600851	>11 (2)	11 ± 0.71 (2)	**38**	IDR-0033566	>3.7 (3)	0.81 ± 0.77 (3)

Table S2. **Minimum Inhibitory Concentrations.** Compounds were tested for inhibitory activity against *M. tuberculosis* in infected RAW 264.7 cells and cytotoxicity against RAW 264.7 cells. IC_{50} are reported as the mean ± standard deviation. The number of replicates is in parentheses.

4. Chemical Methods

General Methods

Reagents and solvents were purchased from Sigma-Aldrich, Acros Organics, Alfa Aesar, Matrix Scientific, Enamine, or Fisher Scientific and used without further purification. NMR spectra were collected on a 300 MHz Bruker AVANCE 300 system with 5 mm BBI probe. LC-MS was performed on an Agilent 1100 system with Phenomenex Gemini 5 µM C18 column and 0.5% formic acid buffered acetonitrile/water gradient elution.

Abbreviations

DCM – dichloromethane
DMF – *N,N*-dimethylformamide
DMSO – dimethylsulfoxide
EDC – *N*-(3-dimethylaminopropyl)-*N*'-ethylcarbodiimide hydrochloride
EtOAc – ethyl acetate
HATU – 1-(bis(dimetylamino)methylene)-1*H*-1,2,3-triazolo[4,5-b]pyridinium 3-oxid hexafluorophosphate
MeCN – acetonitrile
MeOH – methanol
THF – tetrahydrofuran

N-(2,4-dichlorophenyl)-2-nitrobenzamide (S1)

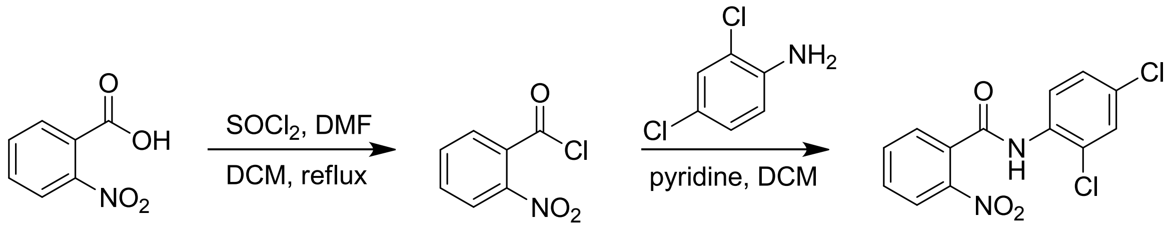

Thionyl chloride (1.89 mL, 26 mmol) was added slowly to a solution of 2-nitrobenzoic acid (3.34 g, 20 mmol) and DMF (2.0 mL, 26 mmol) in DCM (100 mL). The resulting solution was refluxed for 4 h. The reaction mixture was cooled to room temperature and concentrated *in vacuo* to provide the crude acid chloride which was used without further purification.

A solution of the acid chloride prepared above in DCM (100 mL) was added to a solution of 2,4-dichloroaniline (4.21 g, 26 mmol) in DCM (100 mL) and pyridine (20 mL). The resulting solution was stirred overnight.The reaction mixture was diluted with 1 M HCl (aq) and the layers separated. The aqueous layer was back-extracted with one portion of EtOAc. The combined organic layers were washed with brine, dried over Na_2SO_4, and concentrated *in vacuo*. The crude material was purified by flash column chromatography on silica gel (24 g) eluting with a gradient from 0-20% EtOAc in hexanes then 100% EtOAc to give 5.45 g (88%) off-white solid.

2-amino-N-(2,4-dichlorophenyl)benzamide (9)

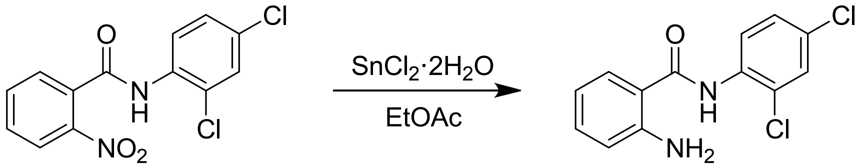

Stannous chloride dihydrate (14.1 g, 62.5 mmol) was added to a solution of *N*-(2,4-dichlorophenyl)-2-nitrobenzamide (3.90 g, 12.5 mmol) in EtOAc (62.5 mL). The resulting solution was stirred overnight. The reaction mixture was diluted with 1 M NaOH (aq) (100 mL) and the layers separated. The organic layer was dried over Na_2SO_4 and concentrated *in vacuo*.The crude material was purified by flash column chromatography on silica gel (40 g) eluting first with DCM then a gradient from 0-20% EtOAc in DCM to give 2.42 g (69%) off-white solid. **^{1}H NMR** (300 MHz, DMSO-d_6) δ = 9.82 (bs, 1H), 7.71 (dd, 8.0, 1.5 Hz, 1H), 7.70 (d, 2.5 Hz, 1H), 7.61 (d, 8.5 Hz, 1H), 7.45 (dd, 8.5, 2.5 Hz, 1H), 7.22 (ddd, 8.5, 7.0, 1.5 Hz, 1H), 6.45 (b, 2H). **LC-MS** (ESI) calculated for $C_{13}H_{10}Cl_2N_2O$ ($[M+H]^+$): 281.02; found: 281.0.

2-amino-N-(4-chlorophenyl)benzamide (S2)

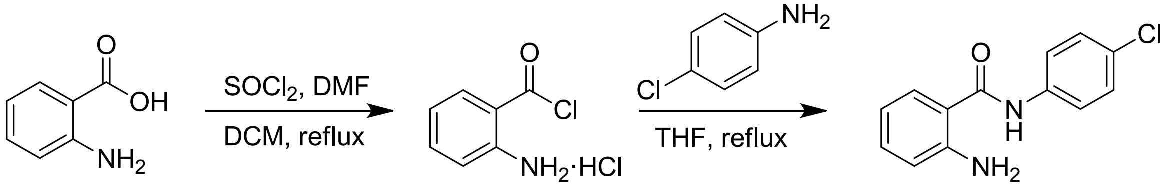

Thionyl chloride (0.55 mL, 7.6 mmol) was added to a suspension of anthranilic acid (0.25 g, 1.8 mmol) in toluene (2.75 mL). The resulting mixture was heated to reflux for 1 h. The reaction mixture was cooled to room temperature and concentrated *in vacuo* to provide the crude acid chloride which was used without further purification.

4-chloroaniline (0.70 g, 5.5 mmol) was added to a solution of the acid chloride prepared above in THF (2.75 mL). The resulting mixture was heated to reflux for 2 h. The reaction mixture was cooled to room temperature, diluted with 10% K_2CO_3 (aq) (10 mL), and stirred for 15 min. The mixture was diluted further with H_2O and extracted with three portions of EtOAc. The combined organic layers were dried over Na_2SO_4 and concentrated *in vacuo*. The crude material was purified by flash column chromatography on silica gel (12 g silica) eluting first with hexanes then a gradient from 0-20% EtOAc in hexanes to give 0.208 g (46%) yellow solid.

2-(4-bromophenylsulfonamido)-N-(4-chlorophenyl)benzamide (3)

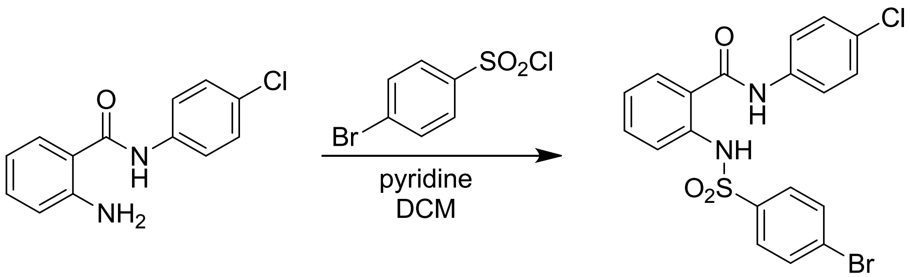

4-bromophenylsulfonyl chloride (236 mg, 0.92 mmol) was added to a solution of 2-amino-*N*-(4-chlorophenyl)benzamide (208 mg, 0.84 mmol) and pyridine (0.203 mL, 2.5 mmol) in DCM (4.2 mL). The resulting solution was stirred overnight. The reaction mixture was diluted with DCM, washed with three portions of 1 M HCl (aq) and one portion of sat. $NaHCO_3$ (aq), dried over Na_2SO_4, and concentrated *in vacuo*. The crude material was purified by flash column chromatography on silica gel (12 g) eluting first with hexanes then a gradient from 0-20% EtOAc in hexanesto give 223 mg (57%) white solid. **^{1}H NMR** (300 MHz, $CDCl_3$) δ = 10.19 (bs, 1H), 7.73 (d, 8.0 Hz, 1H), 7.59 (d, 8.5 Hz, 2H), 7.47 (m, 6H), 7.37 (d, 9.0 Hz, 2H), 7.19 (ddd, 8.5, 8.0, 1.0 Hz, 1H). **LC-MS** (ESI) calculated for $C_{19}H_{14}BrClN_2O_3S$ ($[M+H]^+$): 466.97; found: 466.9.

2-nitro-N-phenylbenzamide (S3)

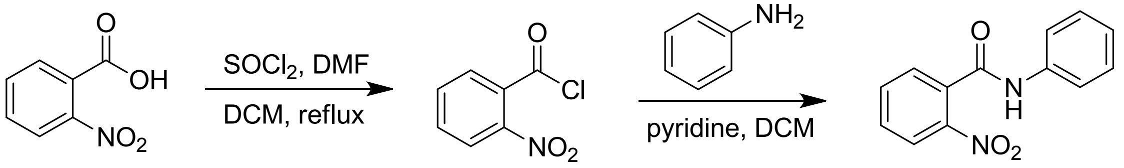

Thionyl chloride (0.47 mL, 6.5 mmol) was added slowly to a solution of 2-nitrobenzoic acid (0.84 g, 5 mmol) and DMF (0.5 mL, 6.5 mmol) in DCM (25 mL). The resulting solution was refluxed overnight. The reaction mixture was cooled to room temperature and concentrated *in vacuo* to provide the crude acid chloride which was used without further purification.

A solution of the acid chloride prepared above in DCM (25 mL) was added to a solution of aniline (0.59 mL, 6.5 mmol) in DCM (25 mL) and pyridine (5 mL). The resulting solution was stirred overnight.The reaction mixture was diluted with 1 M HCl (aq) and the layers separated. The aqueous layer was back-extracted with one portion of EtOAc. The combined organic layers were dried over Na_2SO_4 and concentrated *in vacuo*. The crude material was purified by flash column chromatography on silica gel (12 g) eluting with a gradient from 0-20% EtOAc in hexanes to give an orange-white solid that was used without further purification.

2-amino-N-phenylbenzamide (S4)

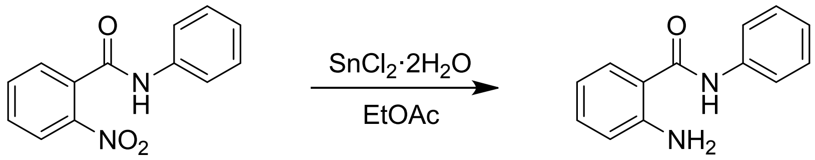

Stannous chloride dihydrate (5.64 g, 25 mmol) was added to a solution of 2-nitro-*N*-phenylbenzamide (1.21 g, 5 mmol) in EtOAc (25 mL). The resulting solution was stirred overnight. The reaction mixture was diluted with 1 M NaOH (aq) (50 mL), stirred to give a suspension, and extracted with EtOAc (100 mL). The organic layer was dried over Na_2SO_4 and concentrated *in vacuo*. The crude material was purified by flash column chromatography on silica gel (24 g) eluting first with hexanes then a gradient from 0-25% EtOAc in hexanes to give 0.888 g (84%) white powder.

2-(4-bromophenylsulfonamido)-N-phenylbenzamide (4)

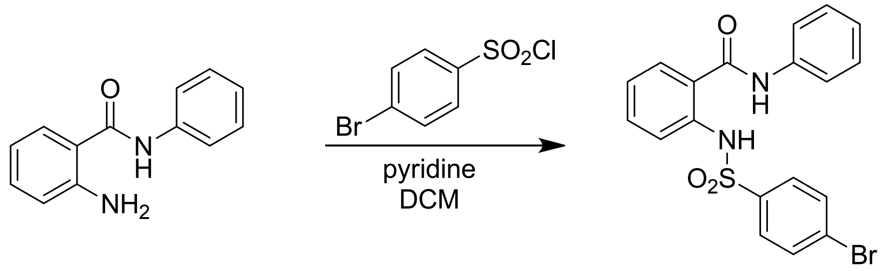

4-bromophenylsulfonyl chloride (132 mg, 0.52 mmol) was added to a solution of 2-amino-*N*-phenylbenzamide (100 mg, 0.47 mmol) and pyridine (114 µL, 1.4 mmol) in DCM (2.35 mL). The resulting solution was stirred for 3 h. The reaction mixture was diluted with DCM, washed with three portions of 1 M HCl (aq) and one portion of sat. $NaHCO_3$ (aq), dried over Na_2SO_4, and concentrated *in vacuo*. The crude material was purified by flash column chromatography on silica gel (4 g) eluting first with hexanes then a gradient from 0-20% EtOAc in hexanesto give 198 mg (97%) white powder. **^{1}H NMR** (300 MHz, $CDCl_3$) δ = 10.29 (bs, 1H), 7.74 (dd, 8.5, 1.0 Hz, 1H), 7.60 (d, 8.5 Hz, 2H), 7.45 (m, 8H), 7.21 (m, 2H). **LC-MS** (ESI) calculated for $C_{19}H_{15}BrN_2O_3S$ ($[M+H]^+$): 433.00; found: 432.9.

2-(4-bromophenylsulfonamido)benzoic acid (S5)

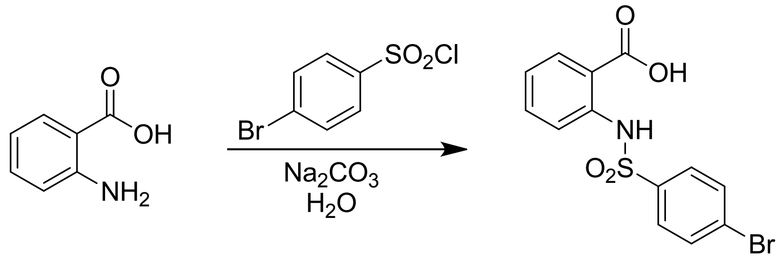

A solution of anthranilic acid (2.74 g, 20 mmol) and Na_2CO_3 (5.09 g, 48 mmol) in H_2O (30 mL) was heated to 60°C. 4-bromophenylsulfonyl chloride (6.13 g, 24 mmol) was added, and the resulting suspension was stirred for 7 h. The reaction mixture was cooled to room temperature, 6 M HCl (aq) (10 mL) added slowly, and the resulting suspension stirred overnight. The precipitate was collected by vaccum filtration, washed with water, and dried *in vacuo* at 40°C to give 7.07 g (99%) tan solid.

2-(4-bromophenylsulfonamido)-N-ethylbenzamide (5)

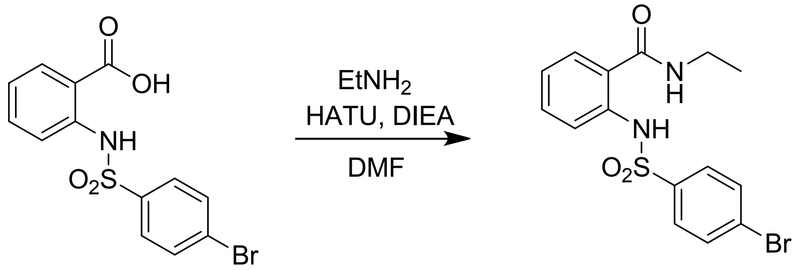

N,*N*-diisopropylethylamine (146 µL, 0.84 mmol) was added to a suspension of 2-(4-bromophenylsulfonamido)benzoic acid (100 mg, 0.28 mmol) and HATU (128 mg, 0.34 mmol) in DMF (2.8 mL). The resulting solution was stirred 10 min. Ethylamine (2 M in THF) (0.7 mL, 1.4 mmol) was added,

and the mixture was stirred overnight. The reaction mixture was diluted with DCM, washed with 1 M HCl (aq), water, sat. $NaHCO_3$ (aq), and brine, dried over Na_2SO_4, and concentrated *in vacuo*. The crude material was purified by flash column chromatography on silica gel (12 g) eluting first with hexanes then a gradient from 0-50% EtOAc in hexanes to give 56 mg yellow oil. The material was further purified by semi-preparative HPLC (C18) eluting with a gradient from 25-65% MeCN in H_2O buffered with 0.5% formic acid to give 28.3 mg (26%) white powder. **^{1}H NMR** (300 MHz, $CDCl_3$) δ = 10.83 (bs, 1H), 7.72 (d, 8.5 Hz, 1H), 7.66 (d, 8.5 Hz, 2H), 7.55 (d, 8.5 Hz, 2H), 7.44 (t, 7 Hz, 1H), 7.35 (d, 9.0 Hz, 1H), 5.94 (b, 1H), 3.38 (qd, 7.5, 5.5 Hz, 2H), 1.22 (t, 7 Hz, 3H). **LC-MS** (ESI) calculated for $C_{15}H_{15}BrN_2O_3S$ ($[M+H]^+$): 384.27; found: 384.9.

2-(4-bromophenylsulfonamido)benzamide (6)

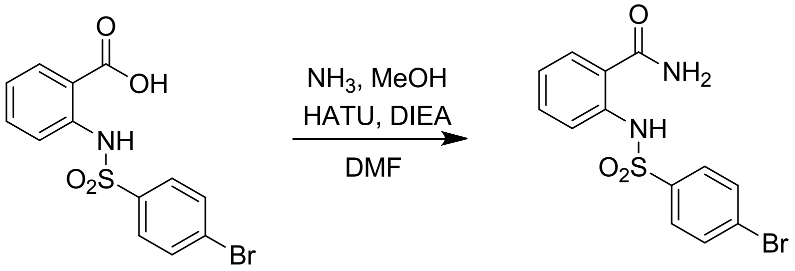

N,*N*-diisopropylethylamine (146 µL, 0.84 mmol) was added to a suspension of 2-(4-bromophenylsulfonamido)benzoic acid (100 mg, 0.28 mmol) and HATU (128 mg, 0.34 mmol) in DMF (2.8 mL). The resulting solution was stirred 10 min. Ammonia (7 M in MeOH) (0.2 mL, 1.4 mmol) was added, and the mixture was stirred overnight. The reaction mixture was diluted with DCM, washed with 1 M HCl (aq), water, sat. $NaHCO_3$ (aq), and brine, dried over Na_2SO_4, and concentrated *in vacuo*. The crude material was purified by flash column chromatography on silica gel (12 g) eluting first with hexanes then a gradient from 0-50% EtOAc in hexanes to give 46.2 mg off-white solid. The material was further purified by semi-preparative HPLC (C18) eluting with a gradient from 5-95% MeCN in H_2O buffered with 0.5% formic acid. The material was further purified by semi-preparative HPLC to (C18) eluting with a gradient from 25-65% MeCN in H_2O buffered with 0.5% formic acid to give 9.8 mg (10%) white powder. **^{1}H NMR** (300 MHz, $CDCl_3$) δ = 11.02 (bs, 1H), 7.73 (d, 7.5 Hz, 1H), 7.69 (d, 8.5 Hz, 2H), 7.55 (d, 8.5 Hz, 2H), 7.47 (d, 8.5 Hz, 1H), 7.43 (t, 7.5 Hz, 1H), 7.10 (t, 7.5 Hz, 1H). **LC-MS** (ESI) calculated for $C_{13}H_{11}BrN_2O_3S$ ($[M+H]^+$): 356.97; found: 356.9.

N-(2,4-dichlorophenyl)-2-(phenylsulfonamido)benzamide (7)

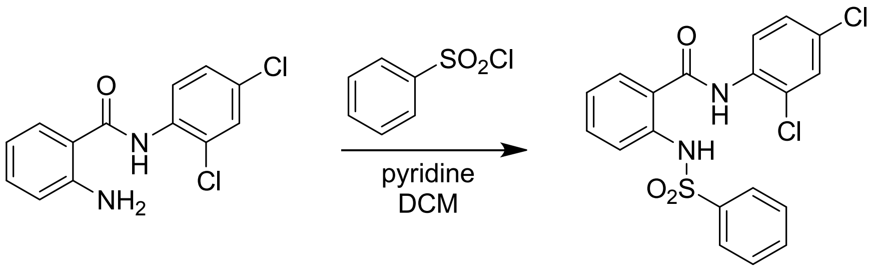

Benzenesulfonyl chloride (21.5 µL, 0.17 mmol) was added to a solution of 2-amino-*N*-(2,4-dichlorophenyl)benzamide (43 mg, 0.15 mmol) and pyridine (37 µL, 0.46 mmol) in DCM (0.77 mL). The

resulting solution was stirred for 2.5 h. The reaction mixture was diluted with DCM, washed with three portions of 1 M HCl (aq) and one portion of sat. $NaHCO_3$ (aq), dried over Na_2SO_4, and concentrated in vacuo. The crude material was purified by flash column chromatography on silica gel (4 g) eluting first with hexanes then with a gradient from 0-25% EtOAc in hexanes. The material was further purified by semi-preparative HPLC on C18 eluting with a gradient from 5-95% MeCN in H_2O buffered with 0.1% formic acid to give 20.7 mg (32%) white powder. **^{1}H NMR** (300 MHz, $CDCl_3$) δ = 10.19 (bs, 1H), 8.35 (d, 9.0 Hz, 1H), 8.00 (b, 1H), 7.76 (m, 3H), 7.51 (m, 2H), 7.43 (m, 2H), 7.32 (m, 3H), 7.19 (td, 8.0, 1.0 Hz, 1H). **LC-MS** (ESI) calculated for $C_{19}H_{14}Cl_2N_2O_3S$ ($[M+H]^+$): 421.02; found: 420.9.

N-(2,4-dichlorophenyl)-2-(methylsulfonamido)benzamide (8)

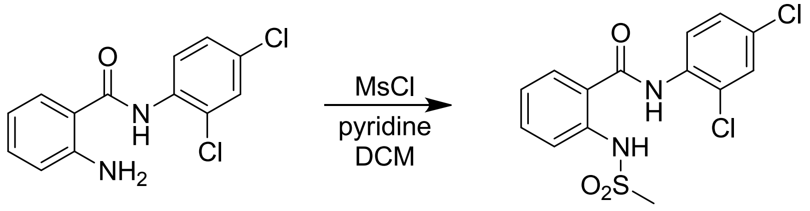

Methanesulfonyl chloride (15.2 µL, 0.2 mmol) was added to a solution of 2-amino-*N*-(2,4-dichlorophenyl)benzamide (50 mg, 0.18 mmol) and pyridine (43 µL, 0.53 mmol) in DCM (0.89 mL). The resulting solution was stirred overnight. The reaction mixture was diluted with DCM, washed with three portions of 1 M HCl (aq) and one portion of sat. $NaHCO_3$ (aq), dried over Na_2SO_4, and concentrated in vacuo. The crude material was purified by semi-preparative HPLC on C18 eluting with a gradient from 5-95% MeCN in H_2O buffered with 0.1% formic acid. The material was further purified by semi-preparative HPLC on C18 eluting with a gradient from 25-60% MeCN in H_2O buffered with 0.1% formic acid to give 15.9 mg white powder. **^{1}H NMR** (300 MHz, $CDCl_3$) δ = 10.34 (bs, 1H), 8.41 (dd, 9.0, 2.5 Hz, 1H), 7.82 (dd, 8.5, 1.0 Hz, 1H), 7.70 (d, 8.0 Hz, 1H), 7.59 (ddd, 8.0, 7.5, 1.0 Hz, 1H), 7.47 (d, 2.5 Hz, 1H), 7.34 (dd, 9.0, 2.5 Hz, 1H), 7.24 (t, 8.0 Hz, 1H), 3.09 (s, 3H). **LC-MS** (ESI) calculated for $C_{14}H_{12}Cl_2N_2O_3S$ ($[M+H]^+$): 359.00; found: 358.9.

N-(2,4-dichlorophenyl)-2-(ethylamino)benzamide (10)

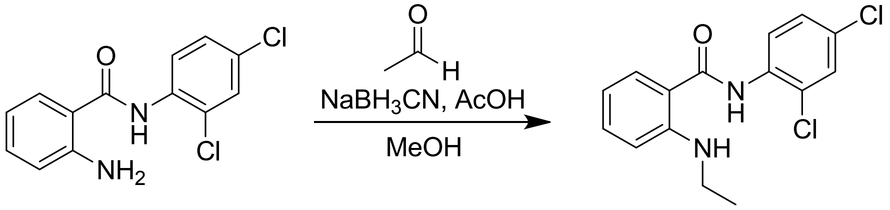

Acetaldehyde (5 M in THF) (0.43 mL, 2.15 mmol) was added to 2-amino-*N*-(2,4-dichlorophenyl)benzamide (200 mg, 0.71 mmol) and acetic acid (30.6 µL, 0.53 mmol) in MeOH (2.8 mL). Sodium cyanoborohydride (145 mg, 2.3 mmol) was added portionwise. The resulting mixture was stirred overnight. The reaction mixture was diluted with water and extracted with three portions of EtOAc. The combined organic layers were dried over Na_2SO_4 and concentrated *in vacuo*. The crude residue was purified by flash column chromatography on silica gel (12 g) eluting first with hexanes then a gradient from 0-20% EtOAc in hexanes to give 73.2 mg (22%) amorphous solid. **^{1}H NMR** (300 MHz, $CDCl_3$) δ = 8.39

(d, 9.0 Hz, 1H), 8.26 (b, 1H), 7.52 (d, 8.0 Hz, 1H), 7.42 (d, 2.5 Hz, 1H), 7.37 (t, 8.0 Hz, 1H), 7.28 (dd, 8.5, 2.0 Hz, 1H), 6.74 (d, 8.5 Hz, 1H), 6.66 (t, 7.0 Hz, 1H), 3.23 (q, 7.0 Hz, 1H), 3.21 (q, 7.0 Hz, 1H), 1.32 (t, 7.0 Hz, 3H). **LC-MS** (ESI) calculated for $C_{15}H_{14}Cl_2N_2O$ ($[M+H]^+$): 309.06; found: 309.0.

N-(2,4-dichlorophenyl)-2-(methylamino)benzamide (S6)

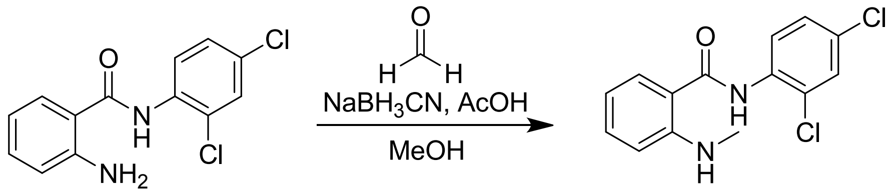

Formaldehyde (37 wt% in H_2O) (29.2 µL, 0.39 mmol) was added to 2-amino-*N*-(2,4-dichlorophenyl)benzamide (100 mg, 0.36 mmol) and acetic acid (15.3 µL, 0.27 mmol) in MeOH (1.78 mL). Sodium cyanoborohydride (44.6 mg, 0.71 mmol) was added portionwise. The resulting mixture was stirred overnight. LC-MS showed incomplete conversion. Additional formaldehyde (37 wt% in H_2O) (29.2 µL, 0.39 mmol) and sodium cyanoborohydride (44.6 mg, 0.71 mmol) were added. The resulting mixture was stirred overnight. The reaction mixture was diluted with water and extracted with three portions of EtOAc. The combined organic layers were dried over Na_2SO_4 and concentrated *in vacuo*. The crude residue was purified by flash column chromatography on silica gel (4 g) eluting first with hexanes then a gradient from 0-20% EtOAc in hexanes to give 63 mg (60%) amorphous solid.

N-(2,4-dichlorophenyl)-2-(ethyl(methyl)amino)benzamide (11)

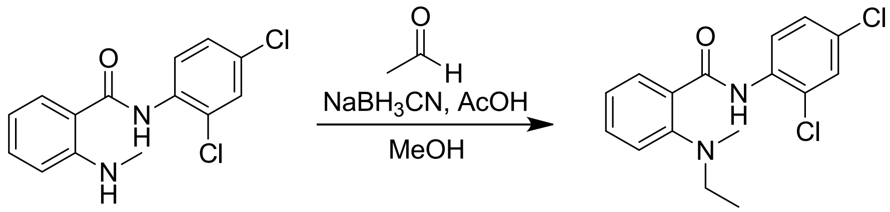

Acetaldehyde (5 M in THF) (126 µL, 0.63 mmol) was added to *N*-(2,4-dichlorophenyl)-2-(methylamino)benzamide (63 mg, 0.21 mmol) and acetic acid (9.0 µL, 0.16 mmol) in MeOH (4.2 mL). Sodium cyanoborohydride (39.6 mg, 0.63 mmol) was added, and the resulting mixture was stirred overnight. LC-MS showed incomplete conversion. Additional acetaldehyde (5 M in THF) (0.42 mL, 2.1 mmol) and sodium cyanoborohydride (132 mg, 2.1 mmol) were added. The resulting mixture was stirred overnight. The reaction mixture was diluted with water and extracted with three portions of EtOAc. The combined organic layers were dried over Na_2SO_4 and concentrated *in vacuo*. The crude residue was purified by semi-preparative HPLC (C18) eluting with a gradient from 5-95% MeCN in H_2O to give 10.8 mg (16%) yellow oil. **^{1}H NMR** (300 MHz, $CDCl_3$) δ = 13.61 (bs, 1H), 8.73 (d, 9.0 Hz, 1H), 8.34 (dd, 8.0, 1.5 Hz, 1H), 7.53 (ddd, 8.0, 6.5, 1.5 Hz, 1H), 7.40 (d, 2.5 Hz, 1H), 7.33 (m, 2H), 3.11 (q, 7.0 Hz, 2H), 2.80 (s, 3H), 1.04 (t, 7.0 Hz, 3H). **LC-MS** (ESI) calculated for $C_{16}H_{17}Cl_2N_2O$ ($[M+H]^+$): 323.07; found: 323.1.

2-((4-bromobenzyl)amino)-N-(2,4-dichlorophenyl)benzamide (12)

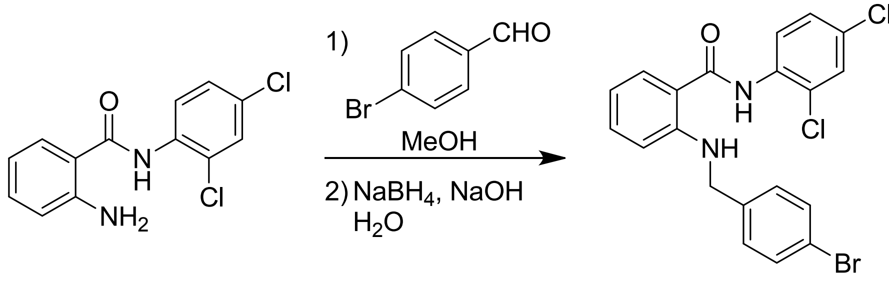

2-amino-*N*-(2,4-dichlorophenyl)benzamide (100 mg, 0.36 mmol) and 4-bromobenzaldehyde (100 mg, 0.54 mmol) were dissolved in MeOH (1.8 mL). The resulting suspension was stirred overnight, during which time a precipitate formed. Additional MeOH (1.8 mL) was added to give a slurry followed by NaOH (2.9 mg, 0.07 mmol), H_2O (180 µL), and sodium borohydride (23.8 mg, 0.63 mmol). The resulting mixture was stirred 1.5 h. The reaction mixture was diluted with water and extracted with three portions of DCM. The combined organic layers were washed with sat. $NaHCO_3$ (aq), dried over Na_2SO_4, and concentrated *in vacuo* to give 128.8 mg (80%) white powder. ^{1}**H NMR** (300 MHz, DMSO-d_6) δ = 7.75 (s, 0.8H), 7.71 (d, 7.0 Hz, 1.2H), 7.61-7.46 (m, 3.4H), 7.44-7.30 (m, 4.6H), 6.99 (d, 8.0 Hz, 0.6H), 6.86-6.74 (m, 1.4H), 6.49 (s, 0.4H), 6.11 (s, 0.6H), 3.34 (s, 2H). **LC-MS** (ESI) calculated for $C_{20}H_{15}BrCl_2N_2O$ ($[M+H]^+$): 450.98; found: 448.9.

2-acetamido-N-(2,4-dichlorophenyl)benzamide (13)

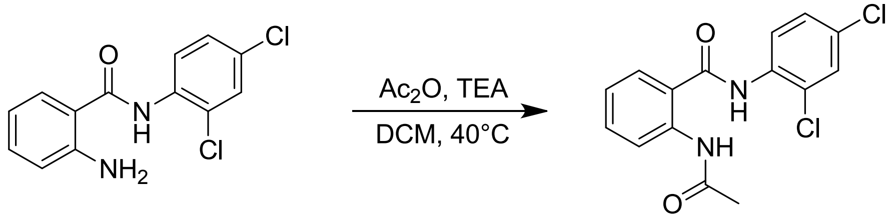

A solution of 2-amino-*N*-(2,4-dichlorophenyl)benzamide (100 mg, 0.36 mmol) in DCM (0.75 mL) was cooled in an ice/water bath. Triethylamine (99 µL, 0.71 mmol) and acetic anhydride (67 µL, 0.71 mmol) were added sequentially. The resulting mixture was heated to 40°C for 2 h. The reaction mixture was diluted with sat. NH_4Cl (aq) and extracted with three portions of DCM. The combined organic layers were washed with sat. $NaHCO_3$ (aq), dried over Na_2SO_4, and concentrated *in vacuo*. The crude material was purified by flash column chromatography on silica gel (12 g) eluting first with hexanes then with a gradient from 0-20% EtOAc in hexanes to give 37 mg white solid. The material was further purified by semi-preparative HPLC (C18) eluting with a gradient from 5-95% MeCN in H_2O buffered with 0.05% formic acid to give 12 mg (10%) white solid. ^{1}**H NMR** (300 MHz, $CDCl_3$) δ = 10.73 (bs, 1H), 8.65 (d, 8.5 Hz, 1H), 8.37 (d, 8.5 Hz, 1H), 8.33 (b, 1H), 7.66 (dd, 8.0, 1.0 Hz, 1H), 7.57 (ddd, 8.5, 8.0, 1.5 Hz, 1H), 7.47 (d, 2.5 Hz, 1H), 7.34 (dd, 9.0, 2.5 Hz, 1H), 7.18 (td, 7.5, 1.5 Hz, 1H), 2.22 (s, 3H). **LC-MS** (ESI) calculated for $C_{15}H_{12}Cl_2N_2O_2$ ($[M+2H]^{2+}$):162.02; found: 162.0.

2-(4-bromobenzamido)-N-(2,4-dichlorophenyl)benzamide (14)

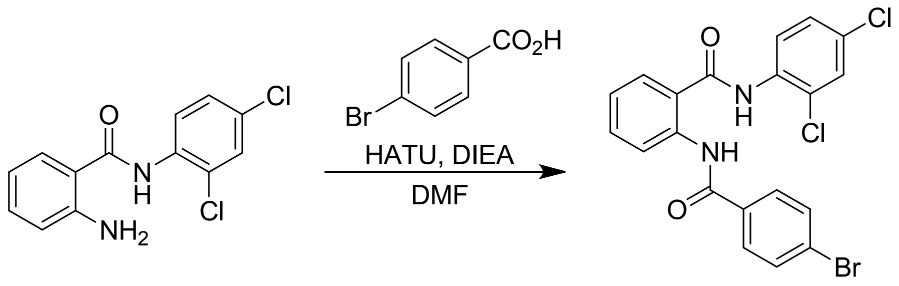

N,*N*-diisopropylethylamine (121 µL, 0.89 mmol) was added to a solution of 2-amino-*N*-(2,4-dichlorophenyl)benzamide (100 mg, 0.36 mmol), 4-bromobenzoic acid (86 mg, 0.43 mmol), and HATU (162 mg, 0.43 mmol) in DMF (1.78 mL). The resulting solution was stirred for 2 h. LC-MS showed the reaction was not proceeding past activation of the acid. 4-dimethylaminopyridine (4.5 mg, 0.036 mmol) was added, and the mixture was heated to 50°C overnight. LC-MS showed incomplete conversion. A solution of additional 4-bromobenzoic acid (86 mg, 0.43 mmol) and HATU (162 mg, 0.43 mmol) in DMF (1 mL) was added, and heating to 50°C continued for 3 h. The reaction mixture was diluted with EtOAc, washed with water, 1 M HCl (aq), water, and sat. $NaHCO_3$ (aq), dried over Na_2SO_4, and concentrated *in vacuo*. The crude material was purified by flash column chromatography on silica gel (12 g) eluting first with hexanes then a gradient from 0-20% EtOAc in hexanes to give 63.3 mg (38%) white solid. **^{1}H NMR** (300 MHz, $CDCl_3$) δ = 11.88 (bs, 1H), 8.86 (dd, 8.5, 1.5 Hz, 1H), 8.43 (s, 1H), 8.41 (d, 9.0 Hz, 1H), 7.90 (d, 8.5 Hz, 2H), 7.73 (dd, 8.0, 1.0 Hz, 1H), 7.66 (d, 8.5 Hz, 2H), 7.63 (dd, 7.0, 1.5 Hz, 1H), 7.47 (d, 2.0 Hz, 1H), 7.35 (dd, 9.0, 2.5 Hz, 1H), 7.24 (td, 7.5, 1.0 Hz, 1H). **LC-MS** (ESI) calculated for $C_{20}H_{13}BrCl_2N_2O_2$ ($[M+Na]^+$):486.94; found: 486.8.

N-(2,4-dichlorophenyl)-2-(4-methylphenylsulfonamido)benzamide (15)

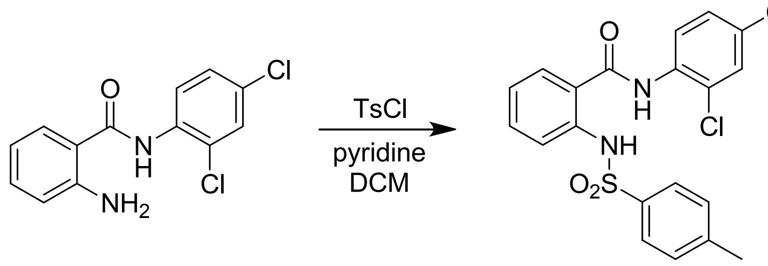

Toluenesulfonyl chloride (75 mg, 0.39 mmol) was added to a solution of 2-amino-*N*-(2,4-dichlorophenyl)benzamide (100 mg, 0.36 mmol) and pyridine (86 µL, 1.07 mmol) in DCM (1.8 mL). The resulting solution was stirred for 3 h. The reaction mixture was diluted with DCM, washed with 1 M HCl (aq) and sat. $NaHCO_3$ (aq), dried over Na_2SO_4, and concentrated in vacuo. The crude material was purified by flash column chromatography on silica gel (4 g) eluting first with hexanes then with a gradient from 0-20% EtOAc in hexanes to give 51.1 mg (33%) white powder. **^{1}H NMR** (300 MHz, $CDCl_3$) δ = 10.07 (bs, 1H), 8.37 (d, 9 Hz, 1H), 8.00 (b, 1H), 7.75 (dd, 8.5, 1 Hz, 1H), 7.63 (d, 8.5 Hz, 2H), 7.51 (m, 2H), 7.44 (d, 2.5 Hz, 1H), 7.34 (dd, 9, 2 Hz, 1H), 7.20 (td, 7.5, 1 Hz, 1H), 7.10 (d, 8 Hz, 2H), 2.26 (s, 3H). **LC-MS** (ESI) calculated for $C_{20}H_{16}Cl_2N_2O_3S$ ($[M+H]^+$): 435.03; found: 435.0.

2-(4-bromophenylsulfonamido)-N-butylbenzamide (16)

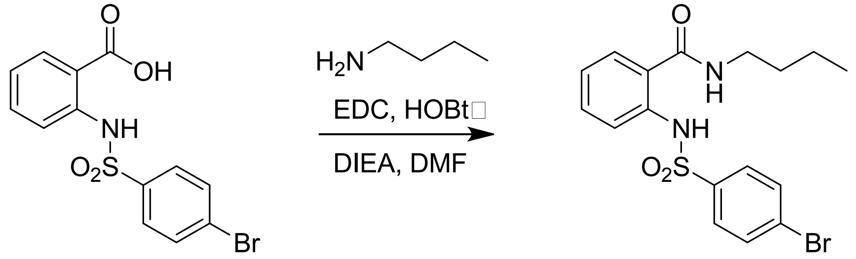

n-Butylamine (55 µL, 0.56 mmol) and *N*,*N*-diisopropylethylamine (146 µL, 0.84 mmol) were added to a suspension of 2-(4-bromophenylsulfonamido)benzoic acid (100 mg, 0.28 mmol), EDC (80 mg, 0.42 mmol), and 1-hydroxybenzotriazole hydrate (67 mg, 0.42 mmol) in DMF (2.8 mL). The resulting solution was stirred overnight. The reaction mixture was diluted with EtOAc, washed with three portions of half-saturated brine, dried over Na_2SO_4, and concentrated *in vacuo*. The crude material was purified by flash column chromatography on silica gel (4 g silica) eluting with a gradient from 0-25% EtOAc in hexanes to give 49.0 mg (43%) white solid. **^{1}H NMR** (300 MHz, $CDCl_3$) δ = 10.89 (s, 1H), 7.68 (dd, 8.5, 1.0 Hz, 1H), 7.63 (d, 9.0 Hz, 2H), 7.52 (d, 8.5 Hz 2H), 7.41 (td, 7.5, 1.5 Hz, 1H), 7.35 (dd, 8.0, 1.5 Hz, 1H), 7.08 (td, 8.0, 1.0 Hz, 1H), 6.08 (bs, 1H), 3.31 (q, 7.0 Hz, 2H), 1.53 (quint, 8.0 Hz, 2H), 1.38 (quint, 7.5 Hz, 2H), 0.96 (t, 7.5 Hz, 3H). **LC-MS** (ESI) calculated for $C_{17}H_{19}BrN_2O_3S$ ($[M+H]^+$): 411.04; found: 413.0.

2-(4-bromophenylsulfonamido)-N-cyclopropylbenzamide (17)

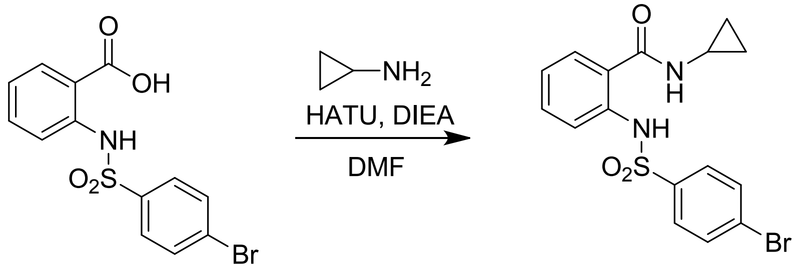

Cyclopropylamine (29.1 µL, 0.42 mmol) and *N*,*N*-diisopropylethylamine (146 µL, 0.84 mmol) were added to a suspension of 2-(4-bromophenylsulfonamido)benzoic acid (100 mg, 0.28 mmol) and HATU (128 mg, 0.34 mmol) in DMF (2.8 mL). The resulting solution was stirred overnight. The reaction mixture was diluted with DCM, washed with 1 M HCl (aq), water, sat. $NaHCO_3$ (aq), and brine, dried over Na_2SO_4, and concentrated *in vacuo*. The crude material was purified by flash column chromatography on silica gel (12 g) eluting first with hexanes then a gradient from 0-40% EtOAc in hexanes to give 87.9 mg (51%) orange oil. The material was further purified by semi-preparative HPLC (C18) eluting with a gradient from 5-95% MeCN in H_2O buffered with 0.05% formic acid to give 44.3 mg (40%) less pure white solid and 9.1 mg (8%) more pure white solid. **^{1}H NMR** (300 MHz, $CDCl_3$) δ = 10.86 (bs, 1H), 7.69 (d, 8.5 Hz, 1H), 7.65 (d, 8.5 Hz, 2H), 7.54 (d, 8.5 Hz, 2H), 7.41 (t, 7.5 Hz, 1H), 7.29 (d, 7.5 Hz, 1H), 7.06 (t, 7.5 Hz, 1H), 6.18 (s, 1H), 2.76 (m, 1H), 0.88 (dd, 13, 6.5 Hz, 2H), 0.55 (dd, 9.5, 7.5 Hz, 2H). **LC-MS** (ESI) calculated for $C_{16}H_{15}BrN_2O_3S$ ($[M+H]^+$): 397.00; found: 397.1.

2-(4-bromophenylsulfonamido)-N-(pro-2-yn-1-yl)benzamide (18)

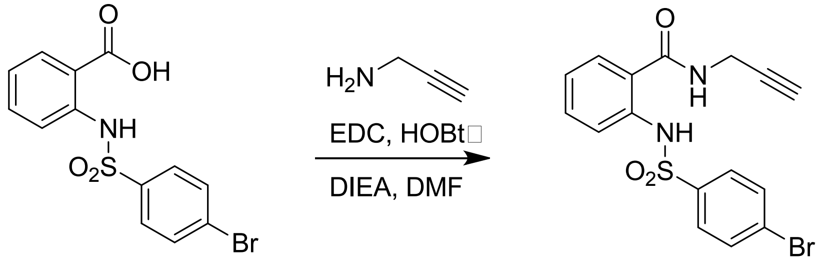

Propargylamine (36 µL, 0.56 mmol) and *N*,*N*-diisopropylethylamine (146 µL, 0.84 mmol) were added to a suspension of 2-(4-bromophenylsulfonamido)benzoic acid (100 mg, 0.28 mmol), EDC (80 mg, 0.42 mmol), and 1-hydroxybenzotriazole hydrate (67 mg, 0.42 mmol) in DMF (2.8 mL). The resulting solution was stirred overnight. The reaction mixture was diluted with DCM, washed with three portions of half-saturated brine, dried over Na_2SO_4, and concentrated *in vacuo*. The crude material was purified by flash column chromatography on silica gel (4 g) eluting with a gradient from 0-25% EtOAc in hexanes to give 54.4 mg (49%) white solid. **^{1}H NMR** (300 MHz, $CDCl_3$) δ = 10.66 (s, 1H), 7.70 (d, 8.5, 1.0 Hz, 1H), 7.65 (d, 9.0 Hz, 2H), 7.54 (d, 9.0 Hz, 2H), 7.45 (td, 7.5, 1.5 Hz, 1H), 7.38 (dd, 8.0, 1.5 Hz, 1H), 7.11 (td, 7.5, 1.0 Hz, 1H), 6.16 (b, 1H), 4.14 (d, 2.5 Hz, 1H), 4.12 (d, 2.5 Hz, 1H), 2.33 (t, 2.5 Hz, 1H). **LC-MS** (ESI) calculated for $C_{16}H_{13}BrN_2O_3S$ ($[M+H]^+$): 394.99; found: 394.9.

2-(4-bromophenylsulfonamido)-N-cycloheptylbenzamide (19)

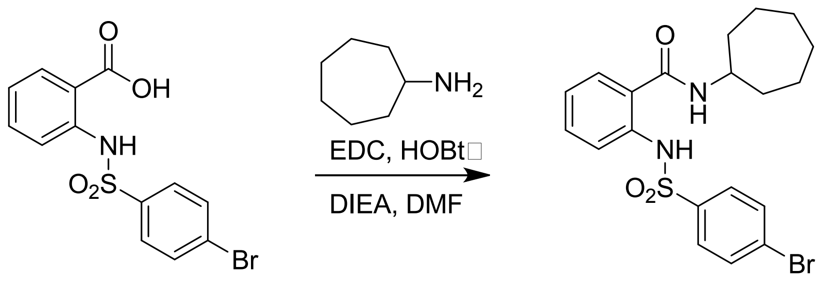

Cycloheptylamine (71 µL, 0.56 mmol) and *N*,*N*-diisopropylethylamine (146 µL, 0.84 mmol) were added to a suspension of 2-(4-bromophenylsulfonamido)benzoic acid (100 mg, 0.28 mmol), EDC (80 mg, 0.42 mmol), and 1-hydroxybenzotriazole hydrate (67 mg, 0.42 mmol) in DMF (2.8 mL). The resulting solution was stirred overnight. The reaction mixture was diluted with DCM, washed with three portions of half-saturated brine, dried over Na_2SO_4, and concentrated *in vacuo*. The crude material was purified by flash column chromatography on silica gel (4 g) eluting with a gradient from 0-25% EtOAc in hexanes to give 64.3 mg (51%) white solid. **^{1}H NMR** (300 MHz, $CDCl_3$) δ = 10.89 (s, 1H), 7.69 (dd, 8.0, 1.0 Hz, 1H), 7.63 (d, 9.0 Hz, 2H), 7.51 (d, 9.0 Hz, 2H), 7.41 (ddd, 8.0, 7.5, 1.5 Hz, 1H), 7.33 (dd, 8.0, 1.5 Hz, 1H), 7.09 (td, 7.5, 1.5 Hz, 1H), 5.95 (d, 7.5 Hz, 1H), 3.97 (tt, 13.0, 4.0 Hz, 1H), 1.91 (m, 2H), 1.54 (m, 10H). **LC-MS** (ESI) calculated for $C_{20}H_{23}BrN_2O_3S$ ($[M+H]^+$): 451.07; found: 453.0.

N-(adamantan-2-yl)-2-(4-bromophenylsulfonamido)benzamide (20)

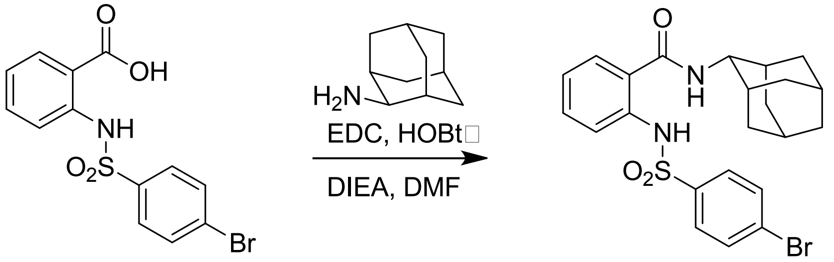

2-adamantylamine hydrochloride (105 mg, 0.56 mmol) and *N*,*N*-diisopropylethylamine (146 µL, 0.84 mmol) were added to a suspension of 2-(4-bromophenylsulfonamido)benzoic acid (100 mg, 0.28 mmol), EDC (80 mg, 0.42 mmol), and 1-hydroxybenzotriazole hydrate (67 mg, 0.42 mmol) in DMF (2.8 mL). The resulting solution was stirred overnight. The reaction mixture was diluted with DCM, washed with three portions of half-saturated brine, dried over Na_2SO_4, and concentrated *in vacuo*. The crude material was purified by flash column chromatography on silica gel (4 g) eluting with a gradient from 0-25% EtOAc in hexanes to give 70.4 mg (51%) white solid. **^{1}H NMR** (300 MHz, $CDCl_3$) δ = 10.84 (s, 1H), 7.72 (dd, 8.0, 1.0 Hz, 1H), 7.63 (d, 9.0 Hz, 2H), 7.51 (d, 9.0 Hz, 2H), 7.44 (t, 7.5 Hz, 1H), 7.37 (d, 8.5, 1.5 Hz, 1H), 7.11 (td, 7.5, 1.0 Hz, 1H), 6.25 (b, 1H), 4.07 (bd, 8.0 Hz), 1.95-1.87 (m, 7H), 1.80-1.76 (m, 2H), 1.73-1.69 (m, 3H). **LC-MS** (ESI) calculated for $C_{23}H_{25}BrN_2O_3S$ ($[M+H]^+$): 491.08; found: 491.0.

4-bromo-N-(2-(piperidine-1-carbonyl)phenyl)benzenesulfonamide (21)

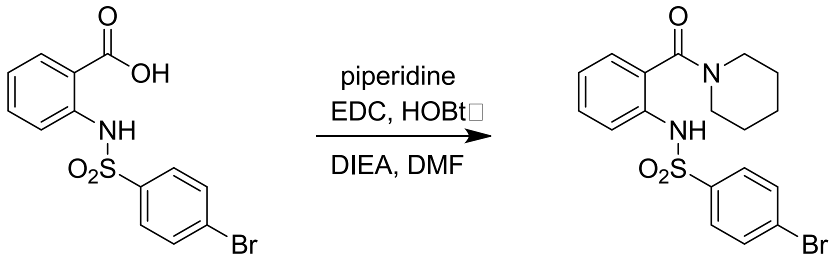

Piperidine (55 µL, 0.56 mmol) and *N*,*N*-diisopropylethylamine (146 µL, 0.84 mmol) were added to a suspension of 2-(4-bromophenylsulfonamido)benzoic acid (100 mg, 0.28 mmol), EDC (80 mg, 0.42 mmol), and 1-hydroxybenzotriazole hydrate (67 mg, 0.42 mmol) in DMF (2.8 mL). The resulting solution was stirred overnight. The reaction mixture was diluted with DCM, washed with three portions of half-saturated brine, dried over Na_2SO_4, and concentrated *in vacuo*. The crude material was purified by flash column chromatography on silica gel (12 g) eluting first with hexanes then with a gradient from 0-20% EtOAc in hexanes to give 54.5 mg (46%) colorless film. **^{1}H NMR** (300 MHz, $CDCl_3$) δ = 8.69 (bs, 1H), 7.68-7.62 (m, 3H), 7.56 (d, 9.0 Hz, 2H), 7.36 (ddd, 9.0, 6.0, 3.0 Hz, 1H), 7.15-7.07 (m, 2H), 3.63-2.93 (b, 4H), 1.63 (b, 2H), 1.48 (b, 2H). **LC-MS** (ESI) calculated for $C_{18}H_{19}BrN_2O_3S$ ($[M+H]^+$): 425.04; found: 425.0.

4-bromo-N-(2-(morpholine-4-carbonyl)phenyl)benzenesulfonamide (22)

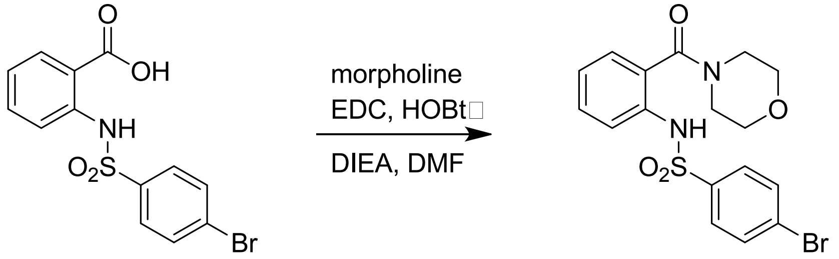

Morpholine (49 µL, 0.56 mmol) and *N*,*N*-diisopropylethylamine (146 µL, 0.84 mmol) were added to a suspension of 2-(4-bromophenylsulfonamido)benzoic acid (100 mg, 0.28 mmol), EDC (80 mg, 0.42 mmol), and 1-hydroxybenzotriazole hydrate (67 mg, 0.42 mmol) in DMF (2.8 mL). The resulting solution was stirred overnight. The reaction mixture was diluted with DCM, washed with three portions of half-saturated brine, dried over Na_2SO_4, and concentrated *in vacuo*. The crude material was purified by flash column chromatography on silica gel (12 g) eluting first with hexanes then with a gradient from 0-20% EtOAc in hexanes to give 60.7 mg (51%) colorless film. **^{1}H NMR** (300 MHz, $CDCl_3$) δ = 7.69 (d, 9.0 Hz, 2H), 7.64-7.57 (m, 3H), 7.38 (ddd, 9.0, 5.5, 3.5 Hz, 1H), 7.15-7.11 (m, 2H), 3.73-3.27 (b, 8H). **LC-MS** (ESI) calculated for $C_{17}H_{17}BrN_2O_4S$ ($[M+H]^+$): 427.02; found: 427.0.

4-bromo-N-(2-(4-methylpiperizine-1-carbonyl)phenyl)benzenesulfonamide (23)

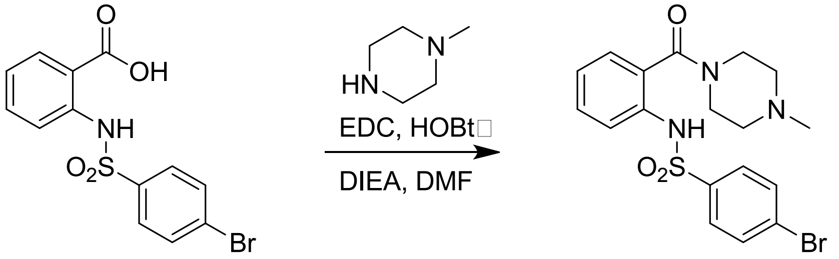

1-methylpiperazine (62 µL, 0.56 mmol) and *N*,*N*-diisopropylethylamine (146 µL, 0.84 mmol) were added to a suspension of 2-(4-bromophenylsulfonamido)benzoic acid (100 mg, 0.28 mmol), EDC (80 mg, 0.42 mmol), and 1-hydroxybenzotriazole hydrate (67 mg, 0.42 mmol) in DMF (2.8 mL). The resulting solution was stirred overnight. The reaction mixture was diluted with DCM, washed with three portions of half-saturated brine, dried over Na_2SO_4, and concentrated *in vacuo*. The crude material was purified by flash column chromatography on silica gel (4 g) eluting first with a gradient from 0-100% EtOAc in hexanes then with a gradient from 0-5% MeOH in EtOAc. The material was further purified by flash column chromatography on silica gel (4 g) eluting with a gradient from 0-2% MeOH in DCM to give 13.8 mg colorless film. **^{1}H NMR** (300 MHz, $CDCl_3$) δ = 7.67 (d, 8.5 Hz, 2H), 7.66 (m, 1H), 7.58 (d, 8.5 Hz, 2H), 7.38 (ddd, 8.5, 6.5, 2.0 Hz, 1H), 7.13 (m, 2H), 3.66 (b, 2H), 3.30 (b, 2H), 2.32 (s, 3H). **LC-MS** (ESI) calculated for $C_{18}H_{20}BrN_3O_3S$ ($[M+H]^+$): 438.05; found: 440.0.

2-nitro-N-(pyridin-2-yl)benzamide (S7)

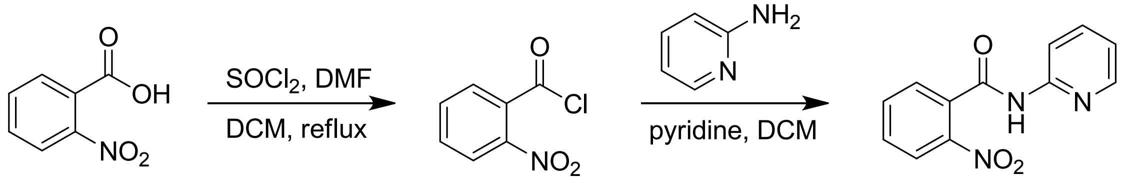

Thionyl chloride (0.47 mL, 6.5 mmol) was added slowly to a solution of 2-nitrobenzoic acid (0.84 g, 5 mmol) and DMF (0.5 mL, 6.5 mmol) in DCM (25 mL). The resulting solution was refluxed for 4 h. The reaction mixture was cooled to room temperature and concentrated *in vacuo* to provide the crude acid chloride which was used without further purification.

A solution of the acid chloride prepared above in DCM (25 mL) was added to a solution of 2-aminopyridine (0.61 g, 6.5 mmol) in DCM (25 mL) and pyridine (5 mL). The resulting solution was stirred overnight.The reaction mixture was diluted with 1 M HCl (aq) and the layers separated. The aqueous layer was back-extracted with one portion of EtOAc. The combined organic layers were washed with brine, dried over Na_2SO_4, and concentrated *in vacuo*. The crude material was purified by flash column chromatography on silica gel (12 g) eluting with a gradient from 0-50% EtOAc in hexanes to give 0.82 g (67%) white solid.

2-amino-N-(2-pyridyl)benzamide (S8)

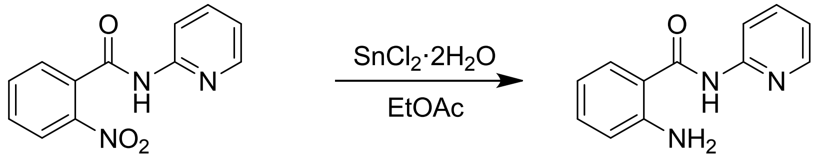

Stannous chloride dihydrate (3.8 g, 16.9 mmol) was added to a solution of 2-nitro-*N*-(pyridin-2-yl)benzamide (0.82 g, 3.4 mmol) in EtOAc (16 mL). The resulting solution was stirred overnight. The reaction mixture was diluted with 1 M NaOH (aq), water, and brine. The resulting emulsion was filtered through a celite plug, washing with DCM, to remove insoluble material. The layers were separated, and the aqeuous layer back-extracted with one portion of DCM. The combined organic layers were dried over Na_2SO_4 and concentrated *in vacuo*. The crude material was purified by flash column chromatography on silica gel (12 g) eluting first with hexanes then a gradient from 0-50% EtOAc in hexanes to give 0.512 g (71%) yellow solid.

2-(4-bromophenylsulfonamido)-N-(pyridin-2-yl)benzamide (24)

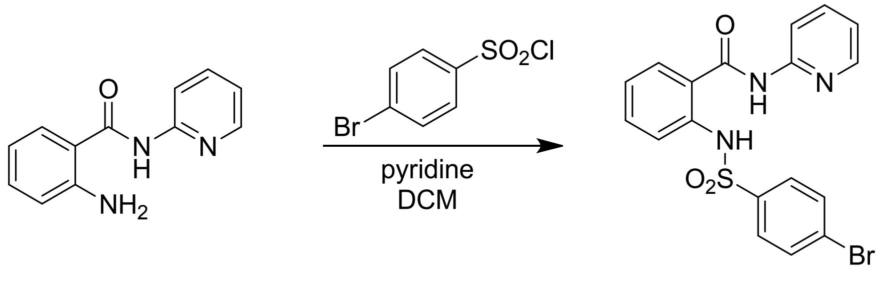

4-bromophenylsulfonyl chloride (132 mg, 0.52 mmol) was added to a solution of 2-amino-*N*-(2-pyridyl)benzamide (100 mg, 0.47 mmol) and pyridine (0.114 mL, 1.4 mmol) in DCM (2.35 mL). The resulting solution was stirred overnight. The reaction mixture was diluted with DCM, washed with 1 M HCl (aq) and sat. $NaHCO_3$ (aq), dried over Na_2SO_4, and concentrated *in vacuo*. The crude material was purified by flash column chromatography on silica gel (4 g) eluting first with hexanes then a gradient from 0-25% EtOAc in hexanesto give 154 mg (76%) white solid. **^{1}H NMR** (300 MHz, $CDCl_3$) δ = 10.30 (bs, 1H), 8.31 (b, 1H), 8.21 (d, 9.0 Hz, 1H), 7.80 (ddd, 9.0, 7.5, 2.0 Hz, 1H), 7.60 (d, 8.5 Hz, 2H), 7.56 (d, 7.0 Hz, 1H), 7.51 (ddd, 8.5, 8.0, 1.5 Hz, 1H), 7.41 (d, 8.5 Hz, 2H), 7.19 (td, 8.0, 1.0 Hz, 1H), 7.14 (dd, 7.5, 5.0 Hz, 1H). **LC-MS** (ESI) calculated for $C_{18}H_{14}BrN_3O_3S$ ($[M+H]^+$): 434.00; found: 433.9.

N-(2,4-dichlorophenyl)-N-methyl-2-nitrobenzamide (S9)

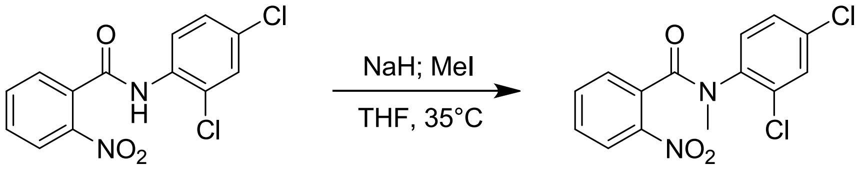

Sodium hydride (60 wt% in mineral oil) (96 mg, 2.4 mmol) was added to a solution of 2-amino-*N*-(2,4-dichlorophenyl)benzamide (0.5 g, 1.6 mmol) in THF (12.3 mL) cooled in an ice/water bath. The bath was removed and the mixture stirred 15 min. Methyl iodide (0.2 mL, 3.2 mmol) was added, and the mixture was heated to 35°C overnight. The reaction mixture was cooled to room temperature and the volatiles removed *in vacuo*. The residue was diluted with 1 M HCl (aq) and extracted with three portions of DCM. The combined organic layers were dried over Na_2SO_4 and concentrated *in vacuo*. The crude material was used without further purification.

2-amino-N-(2,4-dichlorophenyl)-N-methylbenzamide (S10)

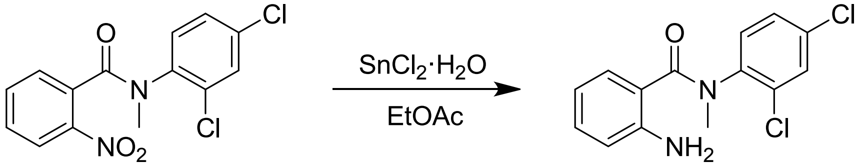

Stannous chloride dihydrate (1.8 g, 8 mmol) was added to a solution of *N*-(2,4-dichlorophenyl)-*N*-methyl-2-nitrobenzamide (0.52 g, 1.6 mmol) in EtOAc (8 mL). The resulting mixture was stirred overnight. The reaction mixture was diluted with 1 M NaOH (aq) and extracted with three portions of EtOAc. The combined organic layers were dried over Na_2SO_4 and concentrated *in vacuo*. The crude material was purified by flash column chromatography on silica gel (12 g) eluting with a gradient from 0-50% EtOAc in DCM to give 0.44 g (94%) colorless film.

2-(4-bromophenylsulfonamido)-N-(2,4-dichlorophenyl)-N-methylbenzamide (25)

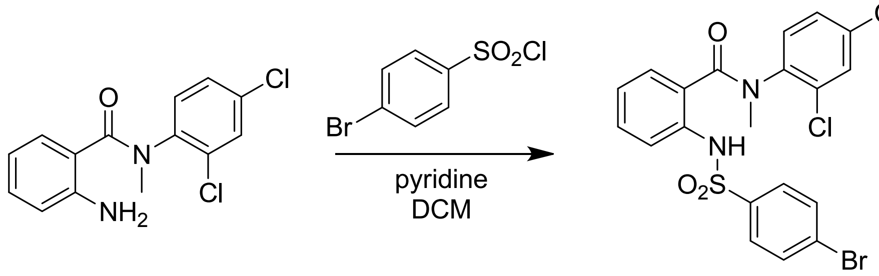

4-bromophenylsulfonyl chloride (0.42 g, 1.7 mmol) was added to a solution of 2-amino-*N*-(2,4-dichlorophenyl)-*N*-methylbenzamide (0.44 g, 1.5 mmol) and pyridine (0.36 mL, 4.5 mmol) in DCM (7.5 mL). The resulting solution was stirred for 1.5 h. The reaction mixture was diluted with DCM, washed with three portion of 1 M HCl (aq) and one portion of sat. $NaHCO_3$ (aq), dried over Na_2SO_4, and concentrated *in vacuo*. The crude material was purified by flash column chromatography on silica gel (12 g) eluting first with hexanes then a gradient from 0-20% EtOAc in hexanes to give 0.62 g (80%) white solid. **^{1}H NMR** (300 MHz, $CDCl_3$) δ = 9.29 (bs, 1H), 7.81 (d, 8.0 Hz, 2H), 7.64 (d, 8.5 Hz, 2H), 7.56 (d, 8.0 Hz, 1H), 7.38 (s, 1H), 7.20 (t, 7.5 Hz, 1H), 6.98 (d, 7.0 Hz, 1H), 6.74 (m, 2H), 6.23 (d, 9.0 Hz, 1H), 3.32 (s, 3H). **LC-MS** (ESI) calculated for $C_{20}H_{15}BrCl_2N_2O_3S$ ($[M+H]^+$): 514.94; found: 514.8.

3-(4-bromophenylsulfonamido)benzoic acid (S11)

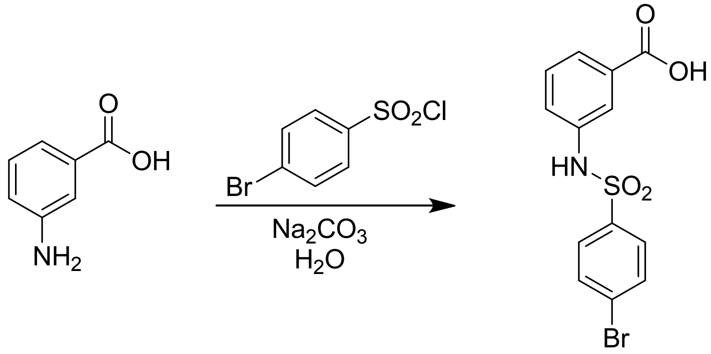

A solution of 3-aminobenzoic acid (0.274 g, 2 mmol) and Na_2CO_3 (0.51 g, 4.8 mmol) in H_2O (3 mL) was heated to 60°C. 4-bromophenylsulfonyl chloride (0.61 g, 2.4 mmol) was added, and the resulting suspension was stirred overnight. The reaction mixture was cooled to room temperature, 6 M HCl (aq) (1 mL) added slowly, and the resulting suspension stirred overnight. The precipitate was collected by vaccum filtration, washed with water, and dried *in vacuo* at 40°C to give 0.388 g (54%) tan solid.

3-(4-bromophenylsulfonamido)-N-(2,4-dichlorophenyl)benzamide (26)

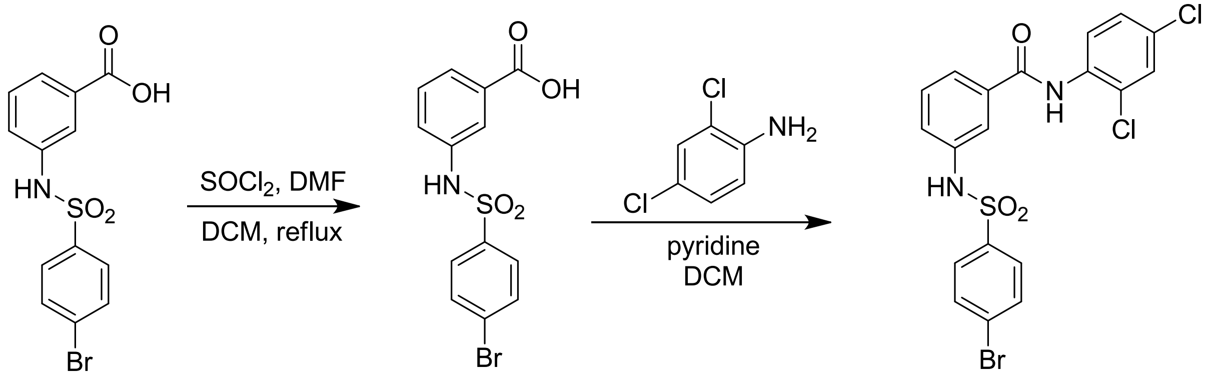

Thionyl chloride (26.4 µL, 0.36 mmol) was added slowly to a solution of 3-(4-bromophenylsulfonamido)benzoic acid (100 mg, 0.28 mmol) and DMF (28 µL, 34 µmol) in DCM (1.4 mL). The resulting solution was refluxed for 4 h. The reaction mixture was cooled to room temperature and concentrated *in vacuo* to provide the crude acid chloride which was used without further purification.

2,4-dichloroaniline (59 mg, 0.36 mmol) and pyridine (0.28 mL) were added to a solution of the acid chloride prepared above in DCM (2.8 mL). The resulting solution was stirred overnight.The reaction mixture was diluted with DCM washed with 1 M HCl (aq), water, and sat. $NaHCO_3$ (aq), dried over Na_2SO_4, and concentrated *in vacuo*. The crude material was purified by flash column chromatography on silica gel (4 g) eluting with a gradient from 0-20% EtOAc in hexanes to give 13 mg (9%) off-white solid. **^{1}H NMR** (300 MHz, DMSO-d_6) δ = 10.64 (bs, 1H), 10.11 (bs, 1H), 7.80 (d, 9.0 Hz, 2H), 7.73 (d, 2.5 Hz, 1H), 7.70 (d, 9.0 Hz, 2H), 7.67 (m, 1H), 7.60 (d, 8.5 Hz, 1H), 7.47 (dd, 8.5, 2.5 Hz, 1H), 7.43 (t, 7.5 Hz, 1H), 7.32 (8.0, 2.0, 1.0 Hz, 1H). **LC-MS** (ESI) calculated for $C_{19}H_{14}BrCl_2N_2O_3S$ ($[M+H]^+$): 500.93; found: 485.8.

N-(2,4-dichlorophenyl)-2-iodobenzamide (S12)

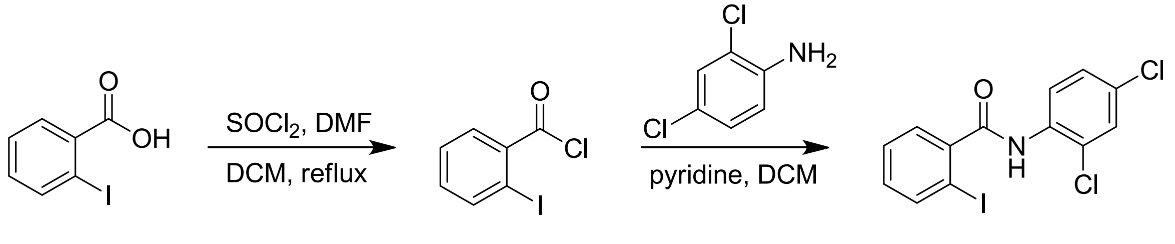

Thionyl chloride (0.47 mL, 6.5 mmol) was added slowly to a solution of 2-iodobenzoic acid (1.24 g, 5 mmol) and DMF (0.5 mL, 6.5 mmol) in DCM (25 mL). The resulting solution was refluxed for 4 h. The reaction mixture was cooled to room temperature and concentrated *in vacuo* to provide the crude acid chloride which was used without further purification.

A solution of the acid chloride prepared above in DCM (25 mL) was added to a solution of 2,4-dichloroaniline (1.05 g, 6.5 mmol) in DCM (25 mL) and pyridine (5 mL). The resulting solution was stirred overnight.The reaction mixture was diluted with 1 M HCl (aq) and the layers separated. The aqueous layer was back-extracted with one portion of EtOAc. The combined organic layers were dried over Na_2SO_4 and concentrated *in vacuo*. The crude material was purified by flash column chromatography on

silica gel (12 g) eluting with a gradient from 0-20% EtOAc in hexanes to give a mixture of product and 2,4-dichloroaniline which was used without further purification.

2-((4-bromophenyl)sulfonyl)-N-(2,4-dichlorophenyl)benzamide (27)

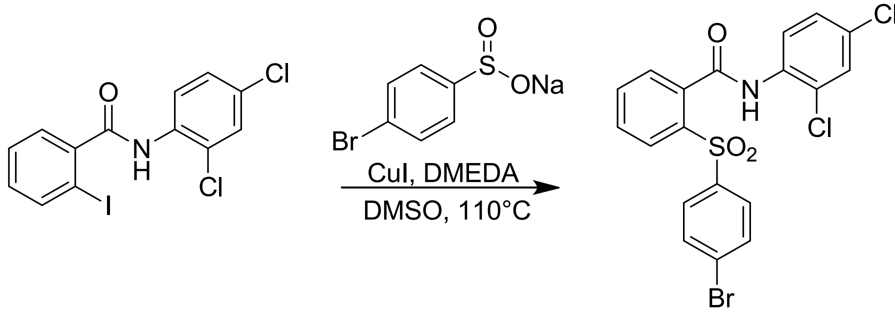

Cupric iodide (4.8 mg, 0.025 mmol) was added to a mixture of *N*-(2,4-dichlorophenyl)-2-iodobenzamide (~30%) (300 mg, 0.25 mmol), sodium 4-bromobenzene-1-sulfinate (124 mg, 0.51 mmol) (prepared per Du et al, Org Lett, 18 (16), 4144-4147, 2016), and 1,2-dimethylethylenediamine (2.7 µL, 0.025 mmol) in DMSO (2.5 mL). The resulting mixture was heated to 110°C overnight. LC-MS showed incomplete conversion. Additional sodium 4-bromobenzene-1-sulfinate (31 mg, 0.13 mmol) and cupric iodide (4.8 mg, 0.025 mmol) were added, and the mixture was heated back to 110°C overnight. The reaction mixture was cooled to room temperature, diluted with water, and extracted with two portions of EtOAc. The combined organic layers were washed with 1 M HCl (aq), sat. $NaHCO_3$ (aq), and two portions of brine, dried over Na_2SO_4, and concentrated *in vacuo*. The crude material was purified by flash column concentration on silica gel (12 g) eluting first with hexanes then a gradient from 0-25% EtOAc in hexanes to give 160 mg purple-white solid which was pure by LC-MS.

A portion of the material was taken up in a 6:1:1 mixture of THF, 8 M Na_2SO_3 (aq), and 8 M $NaHCO_3$ (aq). The resulting mixture was heated to 80°C overnight, during which time the volatibles evaporated. The residue was partitioned between H_2O and EtOAc, the layers separated, and the aqueous layer extracted with one portion of EtOAc. The combined organic layers were dried over Na_2SO_4 and concentrated *in vacuo* to give a white solid. **^{1}H NMR** (300 MHz, $CDCl_3$) δ = 8.39 (d, 9.0 Hz, 1H), 8.18 (dd, 7.0, 2.0 Hz, 1H), 7.94 (b, 1H), 7.86 (d, 8.5 Hz, 2H), 7.67 (td, 7.0, 1.5 Hz, 1H), 7.63 (d, 8.5 Hz, 2H), 7.60 (dd, 6.0, 2.0 Hz, 1H), 7.45 (d, 2.5 Hz, 1H), 7.34 (dd, 9.0, 2.5 Hz, 1H) . **LC-MS** (ESI) calculated for $C_{19}H_{12}BrCl_2NO_3S$ ($[M+H]^+$): 485.92; found: 485.8.

N-(2-aminobenzyl)-2,4-dichloroaniline (S13)

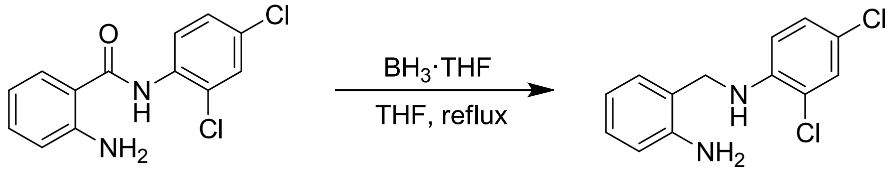

Borane-tetrahydrofuran (2 M in THF) (3.6 mL, 7.2 mmol) was added dropwise to a solution of 2-amino-N-(2,4-dichlorophenyl)benzamide (0.25 g, 0.89 mmol) in THF (1.8 mL) cooled in an ice/water bath. The resulting mixture was stirred for 15 min then heated to reflux overnight. LC-MS showed incomplete

conversion. Additional borane-tetrahydrofuran (2 M in THF) (0.88 mL, 1.76 mmol) was added and reflux was continued for 2 h. The reaction mixture was cooled in a water bath, slowly quenched with 1 M NaOH (aq) (5 mL), and stirred for 15 min. The reaction mixture was partitioned between half-saturated brine and DCM, the layers separated, and the aqueous layer extracted with one portion of DCM. The combined organic layers were dried over Na_2SO_4 and concentrated *in vacuo*. The crude material was purified by flash column chromatography on silica gel (4 g) eluting first with hexanes then a gradient from 0-20% EtOAc in hexanes to give 0.204 g (86%) white solid.

4-bromo-N-(2-(((2,4-dichlorophenyl)amino)methyl)phenyl)benzenesulfonamide (28)

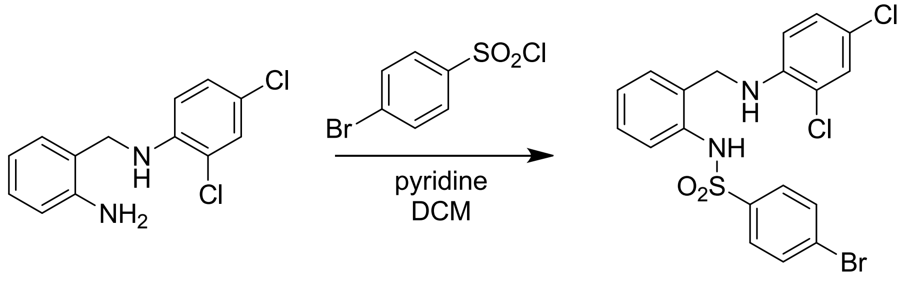

4-bromophenylsulfonyl chloride (0.21 g, 0.84 mmol) was added to a solution of *N*-(2-aminobenzyl)-2,4-dichloroaniline (0.20g, 0.76 mmol) and pyridine (0.18 mL, 2.3 mmol) in DCM (3.8 mL). The resulting solution was stirred for 4.5 h. The reaction mixture was diluted with DCM, washed with three portion of 1 M HCl (aq) and one portion of sat. $NaHCO_3$ (aq), dried over Na_2SO_4, and concentrated *in vacuo*. The crude material was purified by flash column chromatography on silica gel (12 g) eluting first with hexanes then a gradient from 0-25% EtOAc in hexanes to give 0.27 g (74%) white powder. **^{1}H NMR** (300 MHz, DMSO-d_6) δ = 9.96, (s, 1H), 7.81 (d, 8.5 Hz, 2H), 7.62 (d, 8.5 Hz, 2H), 7.35 (d, 2.5 Hz, 1H), 7.15 (m, 3H), 6.99 (d, 2.5 Hz, 1H), 6.96 (d, 2.5 Hz, 1H), 6.28 (t, 5.0, 1H), 5.89 (d, 9.0 Hz, 1H), 4.26 (d, 5.5 Hz, 2H). **LC-MS** (ESI) calculated for $C_{19}H_{15}BrCl_2N_2O_2S$ ($[M+H]^+$): 486.95; found: 486.9.

2-((2,4-dichlorophenoxy)methyl)aniline (S14)

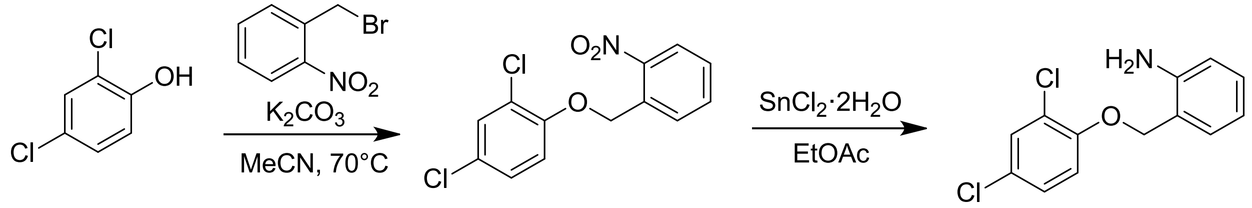

Potassium carbonate (0.42 g, 3 mmol) was added to a solution of 2,4-dichlorophenol (0.25 g, 1.5 mmol) and 2-nitrobenzyl bromide (0.33 g, 1.5 mmol) in MeCN (2.3 mL). The resulting mixture was heated to 70°C for 2 h then cooled to room temperature. The reaction mixture was diluted with water and extracted with three portions of EtOAc. The combined organic layers were washed with water and brine, dried over Na_2SO_4, and concentrated *in vacuo* to give the crude ether which was used without further purification.

Stannous chloride dihydrate (1.73 g, 7.6 mmol) was added to a solution of the crude ether prepared above in EtOAc (7.5 mL). The resulting mixture was stirred overnight. The reaction mixture was diluted

with 1 M NaOH (aq) and extracted with three portions of EtOAc. The combined organic layers were washed with water, dried over Na_2SO_4, and concentrated *in vacuo*. The crude material was purified by flash column chromatography on silica gel (12 g) eluting with a gradient from 0-20% EtOAc in hexanes to give 88.7 mg (22%) material.

4-bromo-N-(2-((2,4-dichlorophenoxy)methyl)phenyl)benzenesulfonamide (29)

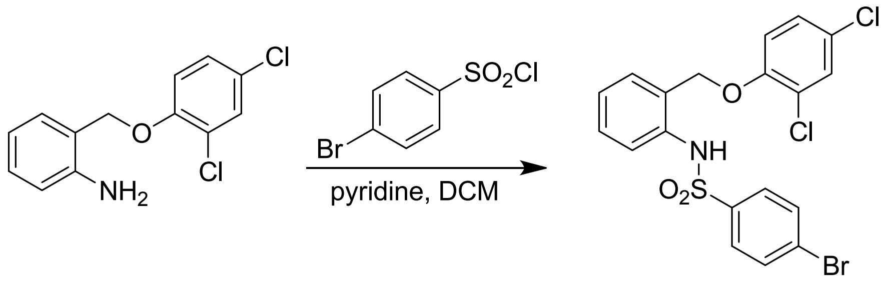

4-bromophenylsulfonyl chloride (93 mg, 0.36 mmol) was added to a solution of 2-((3,4-dichlorophenoxy)methyl)aniline (88.7 mg, 0.33 mmol) and pyridine (80 µL, 0.99 mmol) in DCM (1.65 mL). The resulting solution was stirred for 1.5 h. The reaction mixture was cooled to room temperature, diluted with water, washed with three portions of 1 M HCl (aq) and one portion of sat. $NaHCO_3$ (aq), dried over Na_2SO_4,and concentrated in vacuo. The crude material was purified by flash column chromatography on silica gel (4 g) eluting first with hexanes then a gradient from 0-20% EtOAc in hexanes to give 64.8 mg (40%) white powder. **^{1}H NMR** (300 MHz, $CDCl_3$) δ = 7.60 (d, 9.0 Hz, 2H), 7.61-7.59 (m, 1H), 7.53 (d, 8.5 Hz, 2H), 7.42 (d, 2.5 Hz, 1H), 7.41(dt, 8.0, 1.5 Hz, 1H), 7.33 (td, 7.0, 2.0 Hz, 1H), 7.26 (dd, 7.0, 1.5 Hz, 1H), 7.19 (dd, 9.0, 2.5 Hz, 1H), 7.18 (td, 7.5, 1.5 Hz, 1H), 6.84 (d, 8.5 Hz, 1H), 4.81 (s, 2H). **LC-MS** (ESI) calculated for $C_{19}H_{14}BrCl_2NO_3S$ ($[M+Na]^+$): 509.91; found: 509.8.

cis-2-(4-bromophenylsulfonamido)-cyclohexanecarboxylic acid (S15)

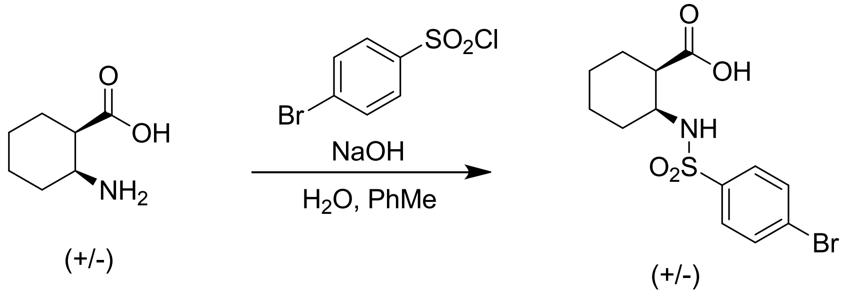

NaOH (60 mg, 1.5 mmol) in H_2O (1 mL) and 4-bromophenylsulfonyl chloride (197 mg, 0.77 mmol) in toluene (1 mL) were added sequentially to a solution of cis-2-aminocyclohexanecarboxylic acid (100 mg, 0.7 mmol) in H_2O (1 mL). The resulting mixture was stirred overnight. The reaction mixture was washed with two portions of toluene, acidified to approximately pH 4 with 1 M HCl (aq), and extracted with three portions of EtOAc. The combined EtOAc extracts were dried over Na_2SO_4 and concentrated *in vacuo* to give 132.6 mg (52%) white solid.

cis-2-(4-bromophenylsulfonamido)-N-(4-chlorophenyl)cyclohexanecarboxamide (30)

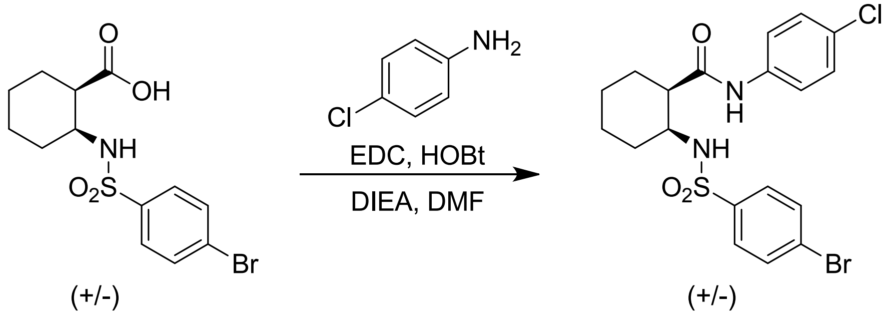

N,*N*-diisopropylethylamine (128 µL, 0.73 mmol) was added to a suspension of cis-2-(4-bromophenylsulfonamido)-cyclohexanecarboxylic acid (132.6 mg, 0.37 mmol), 4-chloroaniline (93 mg, 0.73 mmol), EDC (105 mg, 0.55 mmol), and 1-hydroxybenzotriazole hydrate (87 mg, 0.55 mmol) in DMF (3.7 mL). The resulting solution was heated to 45°C overnight. The reaction mixture was cooled to room temperature, diluted with DCM, washed with 1 M HCl (aq), water, sat. $NaHCO_3$ (aq), and brine, dried over Na_2SO_4, and concentrated *in vacuo*. The crude material was purified by flash column chromatography on silica gel (12 g) eluting first with hexanes then a gradient from 0-20% EtOAc in hexanes to give 51.6 mg tan solid. The material was further purified by preparative HPLC (C18) eluting with a gradient from 10-90% MeCN in H_2O buffered with 1% formic acid to give 25.6 mg (15%) white powder. **^{1}H NMR** (300 MHz, DMSO-d_6) δ = 9.79 (bs, 1H), 7.78 (b, 1H), 7.64 (d, 9.0 Hz, 2H), 7.55 (d, 9.0 Hz, 2H), 7.47 (d, 9.0 Hz, 2H), 7.30 (d, 9.0 Hz, 2H), 3.70 (b, 1H), 2.57 (b, 1H), 1.76 (b, 2H), 1.54 (b, 3H), 1.26 (b, 3H). **LC-MS** (ESI) calculated for $C_{19}H_{20}BrClN_2O_3S$ ($[M+H]^+$): 473.01; found: 473.0.

trans-2-(4-bromophenylsulfonamido)-cyclohexanecarboxylic acid (S16)

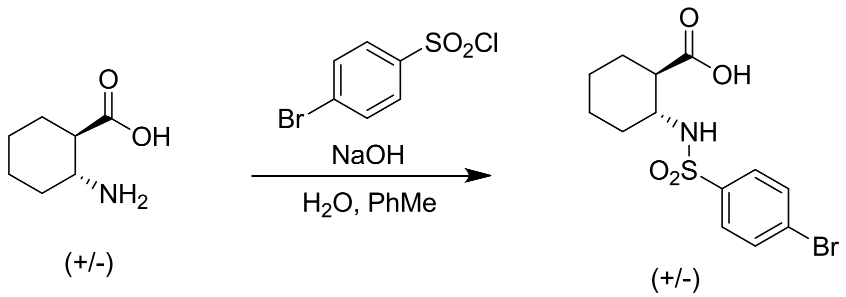

NaOH (30 mg, 0.75 mmol) in H_2O (0.5 mL) and 4-bromophenylsulfonyl chloride (98 mg, 0.39 mmol) in toluene (0.5 mL) were added sequentially to a solution of trans-2-aminocyclohexanecarboxylic acid (50 mg, 0.35 mmol) in H_2O (0.5 mL). The resulting mixture was stirred overnight. The reaction mixture was washed with two portions of toluene, acidified to approximately pH 4 with 1 M HCl (aq), and extracted with three portions of EtOAc. The combined EtOAc extracts were dried over Na_2SO_4 and concentrated *in vacuo*.The residue was taken up in toluene and concentrated *in vacuo* to give 46.2 mg (36%) white solid.

trans-2-(4-bromophenylsulfonamido)-N-(4-chlorophenyl)cyclohexanecarboxamide (31)

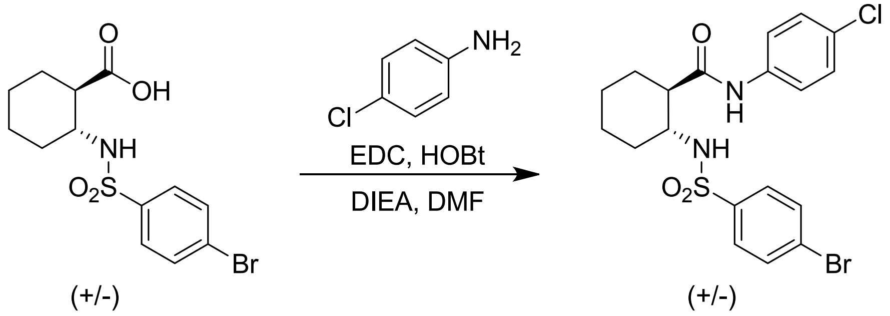

N,*N*-diisopropylethylamine (44.6 µL, 0.26 mmol) was added to a suspension of trans-2-(4-bromophenylsulfonamido)-cyclohexanecarboxylic acid (46.2 mg, 0.13 mmol), 4-chloroaniline (32.7 mg, 0.26 mmol), EDC (36.8 mg, 0.19 mmol), and 1-hydroxybenzotriazole hydrate (30.5 mg, 0.19 mmol) in DMF (1.3 mL). The resulting solution was heated to 45°C overnight. The reaction mixture was cooled to room temperature, diluted with DCM, washed with 1 M HCl (aq), water, sat. $NaHCO_3$ (aq), and brine, dried over Na_2SO_4, and concentrated *in vacuo*. The crude material was purified by flash column chromatography on silica gel (4 g) eluting first with hexanes then a gradient from 0-25% EtOAc in hexanes to give 3.5 mg (6%) tan solid. **^{1}H NMR** (300 MHz, DMSO-d_6) δ = 9.82 (s, 1H), 7.92 (bs, 1H), 7.58 (d, 8.5 Hz, 2H), 7.52 (d, 8.5 Hz, 2H), 7.41 (d, 8.5 Hz, 2H), 7.30 (d, 8.5 Hz, 2H), 3.39 (b, 1H), 2.26 (td, 11.0, 3.5 Hz, 1H), 1.77 (m, 2H), 1.62 (m, 2H), 1.38 (q, 12.0 Hz, 1H), 1.21 (t, 9.5 Hz, 1H), 1.15 (m, 2H). **LC-MS** (ESI) calculated for $C_{19}H_{20}BrClN_2O_3S$ ($[M+H]^+$): 473.01; found: 472.9.

3-amino-N-(4-chlorophenyl)picolinamide (S17)

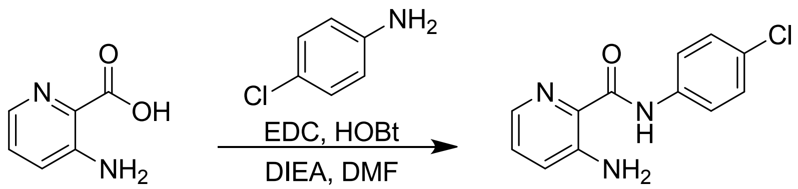

N,*N*-diisopropylethylamine (0.25 mL, 1.4 mmol) was added to a suspension of 3-amino-2-pyridinecarboxylic acid (100 mg, 0.72 mmol), 4-chloroaniline (184 mg, 1.4 mmol), EDC (207 mg, 1.1 mmol), and 1-hydroxybenzotriazole hydrate (172 mg, 1.1 mmol) in DMF (6 mL). The resulting solution was heated to 45°C overnight. The reaction mixture was cooled to room temperature, diluted with DCM, washed with half-saturated brine, sat. $NaHCO_3$ (aq), and half-saturated brine, dried over Na_2SO_4, and concentrated *in vacuo*. The crude material was purified by flash column chromatography on silica gel (12 g) eluting first with hexanes then a gradient from 0-50% EtOAc in hexanes to give a mixture of product and excess 4-chloroaniline which was used without further purification.

3-(4-bromophenylsulfonamido)-N-(4-chlorophenyl)picolinamide (32)

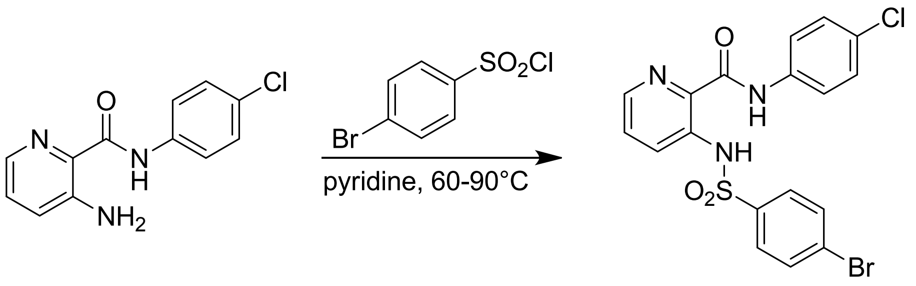

4-bromophenylsulfonyl chloride (184 mg, 0.72 mmol) was added to a solution of crude 3-amino-*N*-(4-chlorophenyl)picolinamide (178 mg, 0.72 mmol) in pyridine (7.2 mL). The resulting solution was heated to 60°C overnight. Additional 4-bromophenylsulfonyl chloride (184 mg, 0.72 mmol) was added, and the solution was heated to 60°C overnight. LC-MS showed the reaction not progressing. The solution was heated to 90°C overnight, during which time the volatiles boiled off. The residue was taken up in DCM, washed with water and brine, dried over Na_2SO_4, and concentrated *in vacuo*. The crude material was purified by flash column chromatography on silica gel (12 g) eluting with a gradient from 0-25% EtOAc in hexanes to give 21.6 mg (6%) white solid. **^{1}H NMR** (300 MHz, $CDCl_3$) δ = 11.86 (bs, 1H), 10.18 (bs, 1H), 8.26 (dd, 4.5, 1.5 Hz, 1H), 8.15 (dd, 8.5, 1.5 Hz, 1H), 7.76 (d, 8.5 Hz, 2H), 7.64 (d, 8.5 Hz, 2H), 7.59 (d, 8.5 Hz, 2H), 7.43 (dd, 8.5, 4.5 Hz, 1H), 7.37 (d, 9.0 Hz, 2H). **LC-MS** (ESI) calculated for $C_{18}H_{13}BrClN_3O_3S$ ($[M+H]^+$): 467.96; found: 467.8.

2-amino-N-(4-chlorophenyl)nicotinamide (S18)

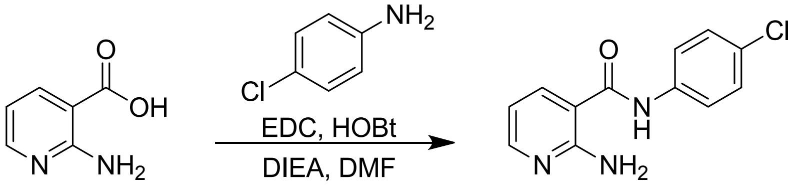

N,*N*-diisopropylethylamine (0.25 mL, 1.4 mmol) was added to a suspension of 2-aminonicotinic acid (100 mg, 0.72 mmol), 4-chloroaniline (184 mg, 1.4 mmol), EDC (207 mg, 1.1 mmol), and 1-hydroxybenzotriazole hydrate (172 mg, 1.1 mmol) in DMF (6 mL). The resulting solution was heated to 45°C overnight. The reaction mixture was cooled to room temperature, diluted with DCM, washed with half-saturated brine, sat. $NaHCO_3$ (aq), and half-saturated brine, dried over Na_2SO_4, and concentrated *in vacuo*. The crude material was purified by flash column chromatography on silica gel (12 g) eluting with a gradient from 0-50% EtOAc in hexanes to give 130 mg (73%) yellow solid.

2-(4-bromophenylsulfonamido)-N-(4-chlorophenyl)nicotinamide (33)

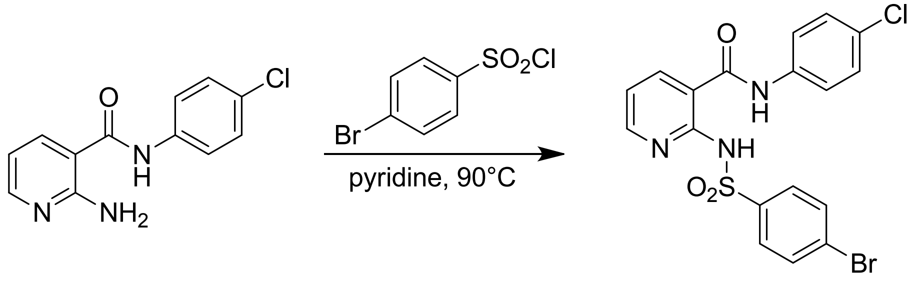

4-bromophenylsulfonyl chloride (0.4 g, 1.6 mmol) was added to a solution of 2-amino-*N*-(4-chlorophenyl)nicotinamide (130 mg, 0.53 mmol) in pyridine (5.25 mL). The resulting solution was heated to 90°C overnight. The reaction mixture was cooled to room temperature, diluted with water, and extracted with three portions of DCM. The combined organic layers were dried over Na_2SO_4 and concentrated in vacuo. The crude material was purified by flash column chromatography on silica gel (12 g) eluting with a gradient from 0-100% EtOAc in hexanes to give 46.3 mg (19%) white solid. **^{1}H NMR** (300 MHz, $CDCl_3$) δ = 11.85 (bs, 1H), 8.92 (d, 7.0 Hz, 1H), 7.84 (d, 8.5 Hz, 2H), 7.74 (d, 5.5 Hz, 1H), 7.66 (d, 9.0 Hz, 2H), 7.52 (d, 9.0 Hz, 2H), 7.30 (d, 8.5 Hz, 1H), 6.94 (t, 7.0 Hz, 1H). **LC-MS** (ESI) calculated for $C_{18}H_{13}BrClN_3O_3S$ ($[M+H]^+$): 467.96; found: 468.0.

5. NMR Spectra of Final Compounds

Compound **3** (^{1}H NMR, 300 MHz, $CDCl_3$)

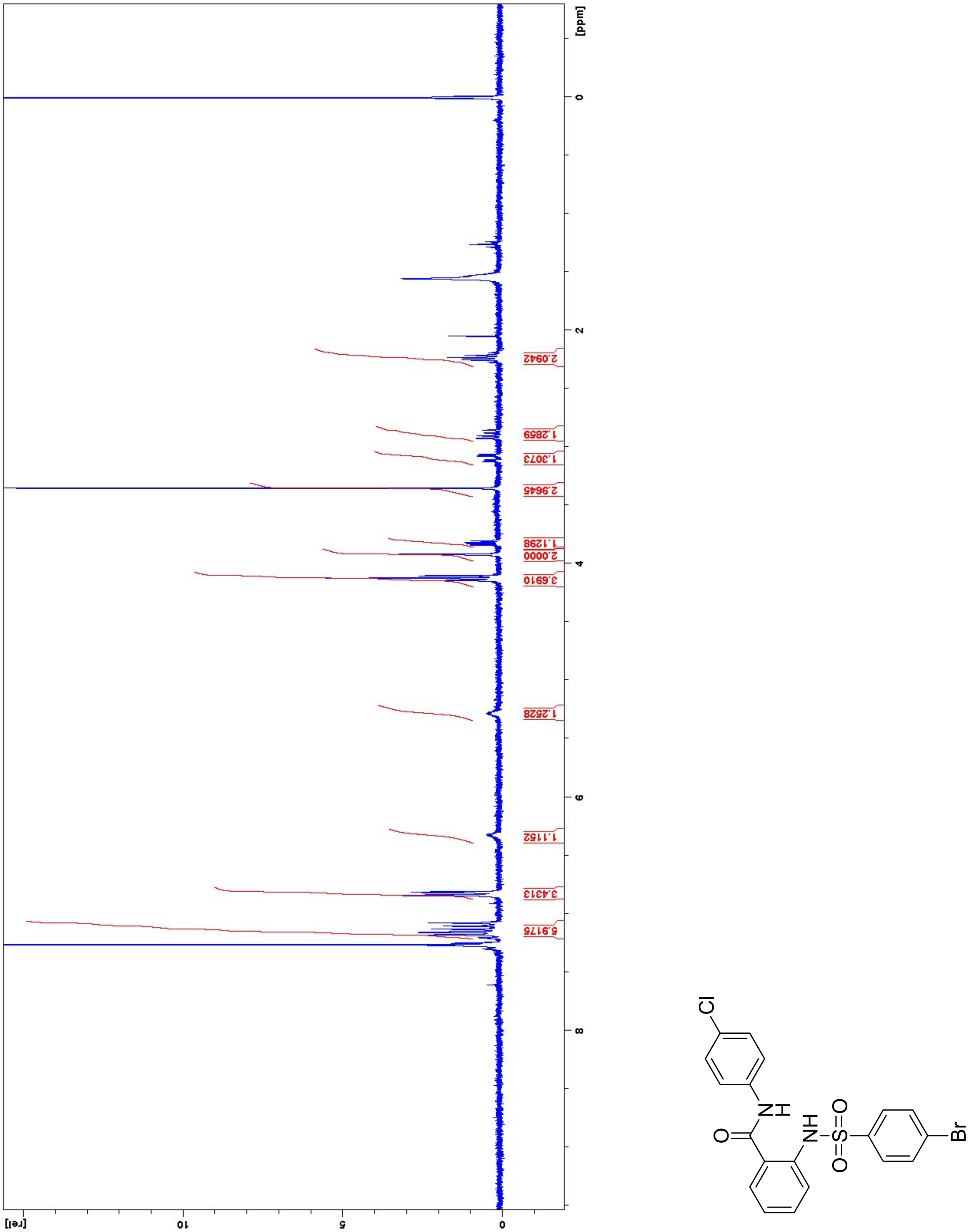

Compound **4** (^{1}H NMR, 300 MH

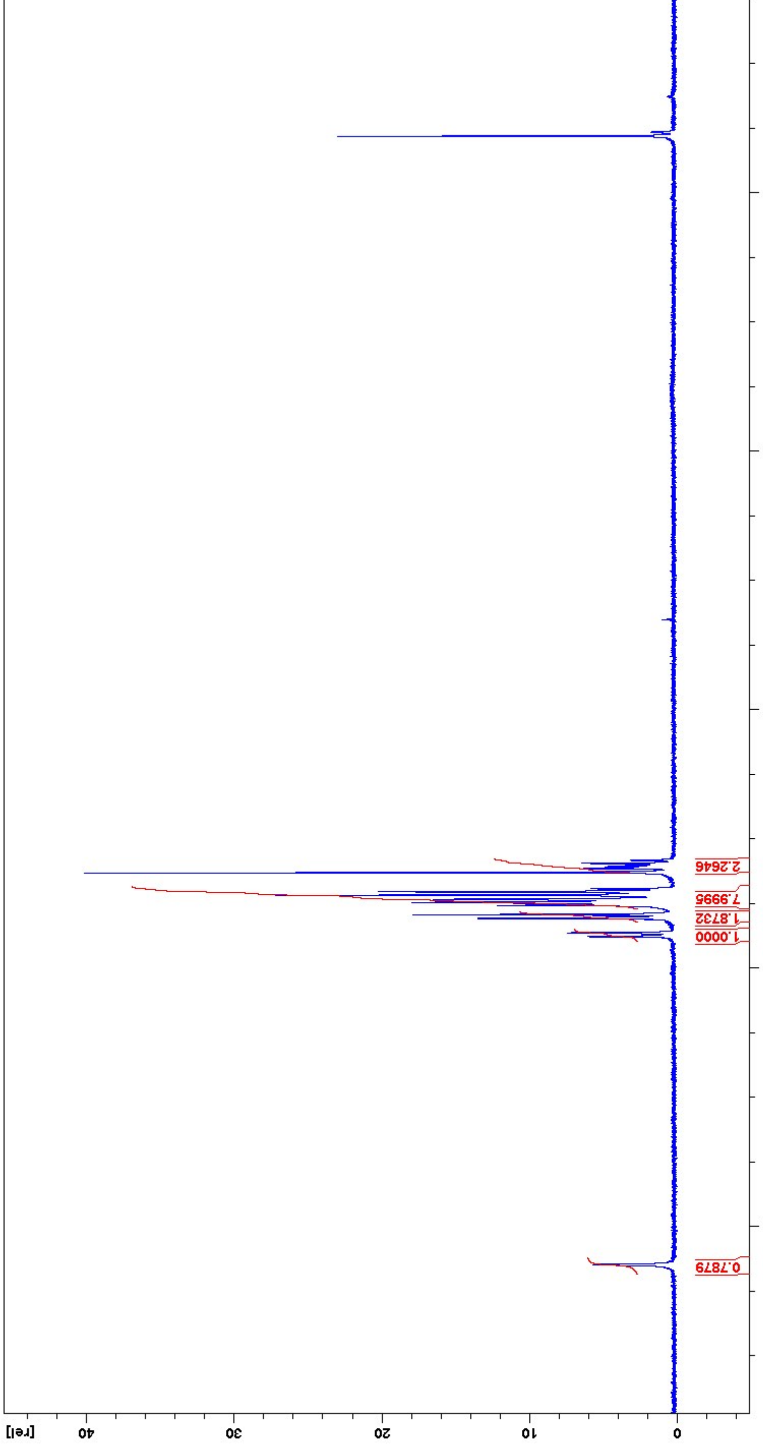

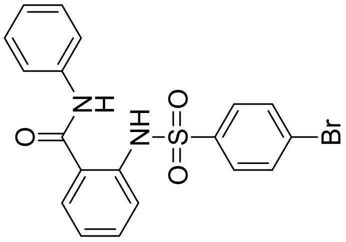

Compound **5** (^{1}H NMR, 300 MHz, $CDCl_3$)

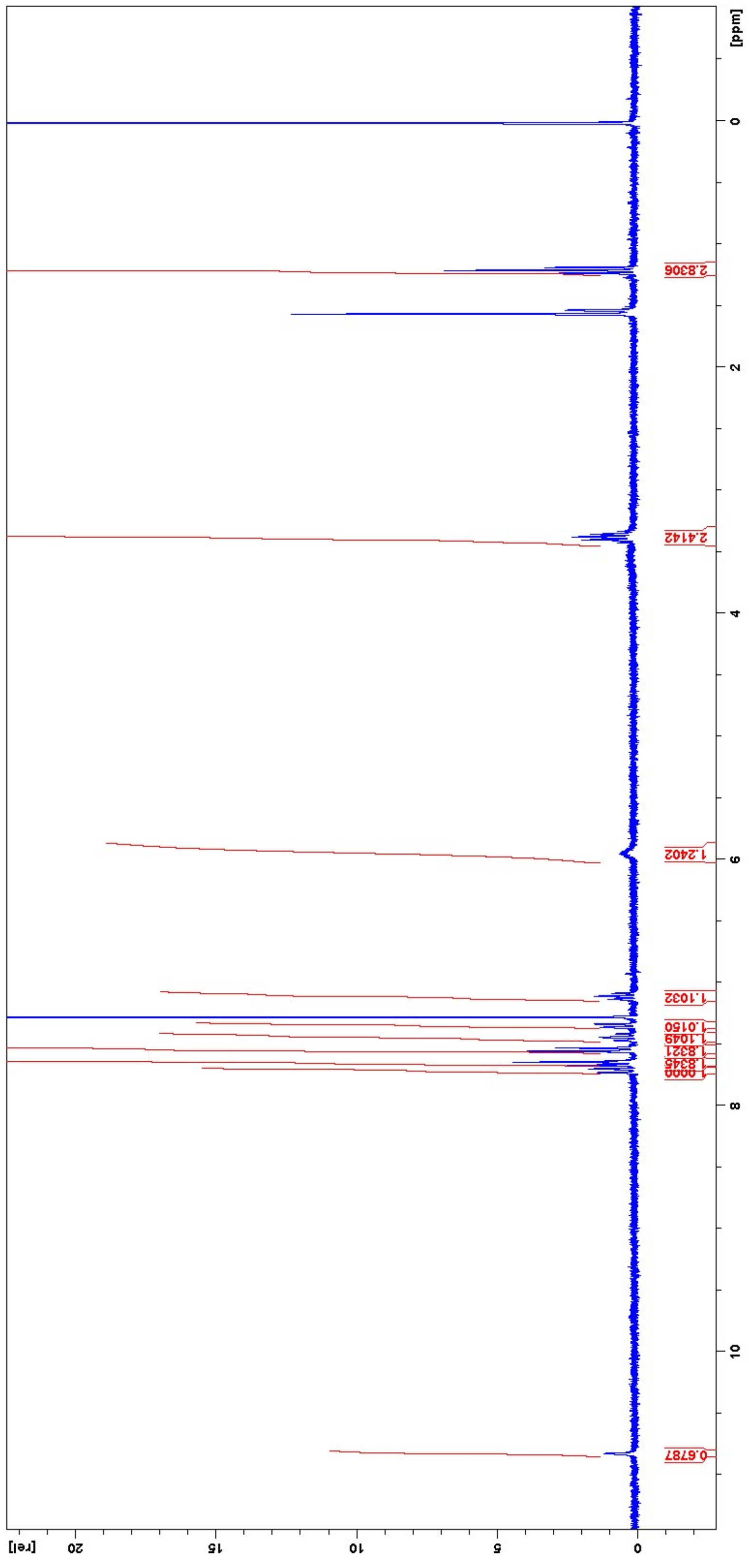

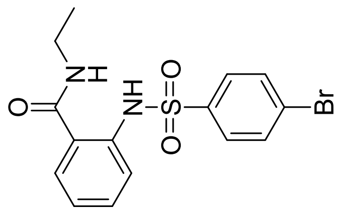

Compound **6** (^{1}H NMR, 300 MHz, $CDCl_3$)

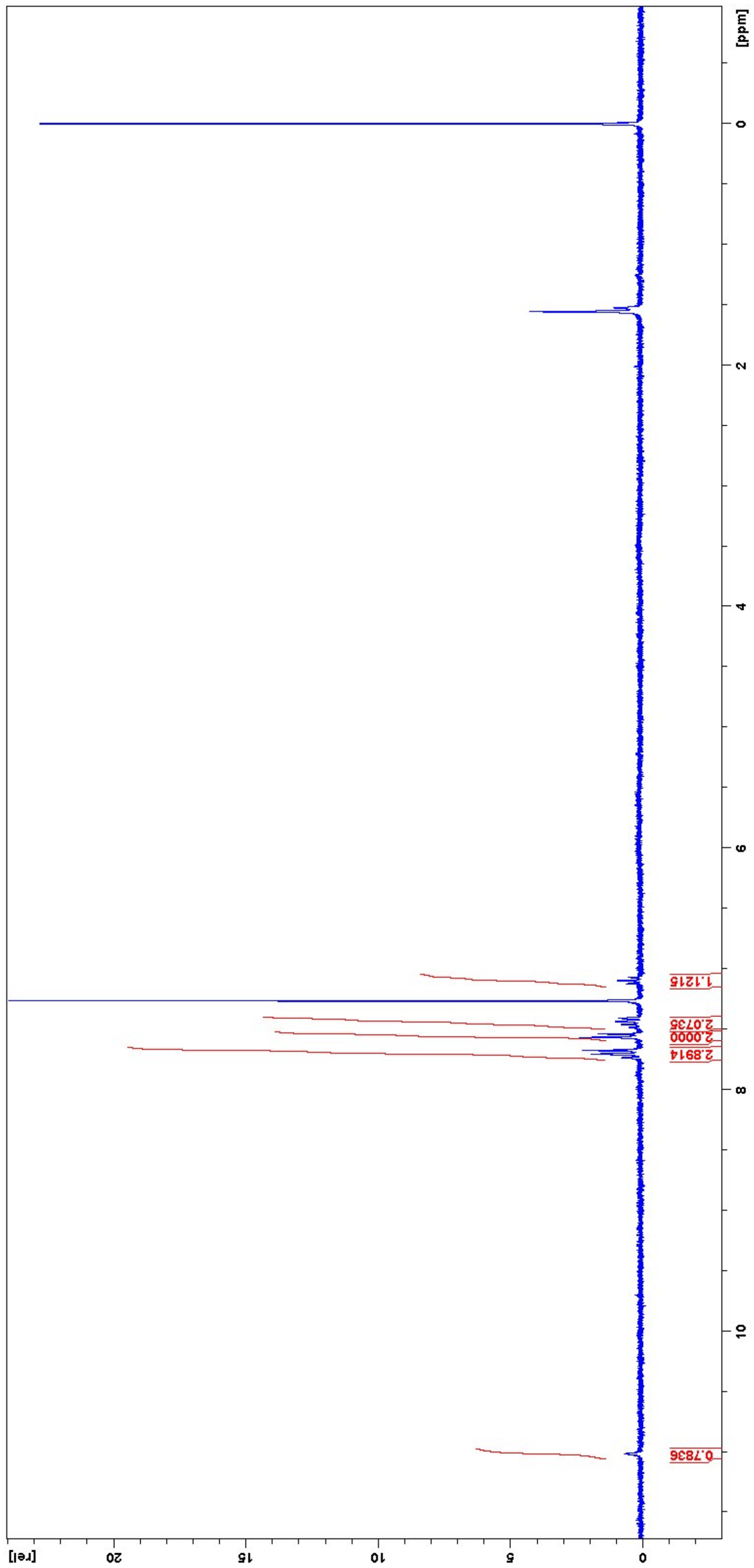

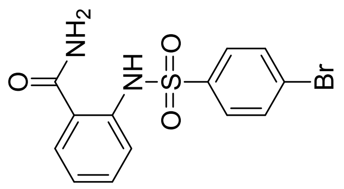

Compound **7** (^{1}H NMR, 300 MH

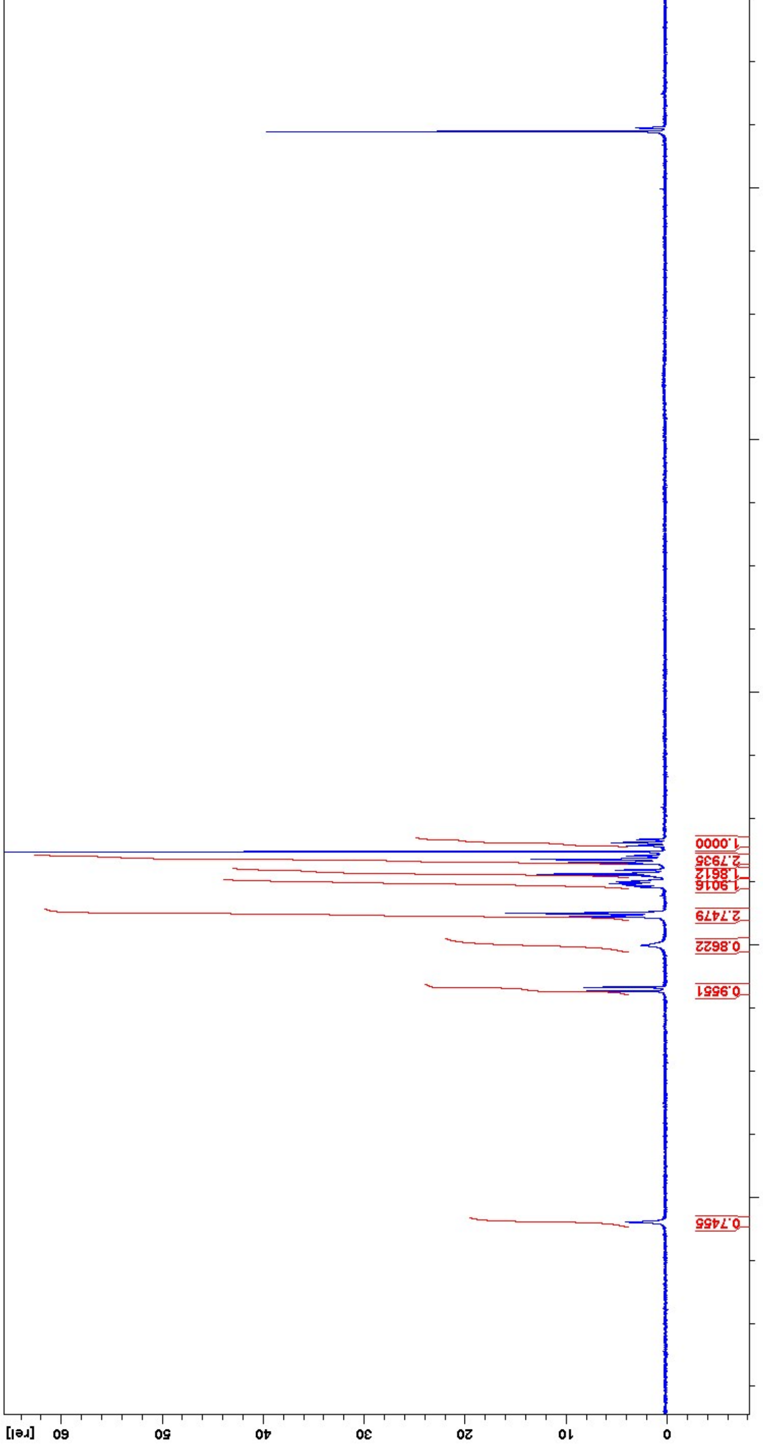

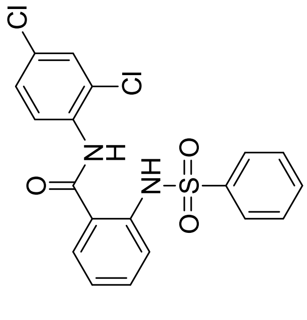

Compound **8** (^{1}H NMR, 300 MHz, $CDCl_3$)

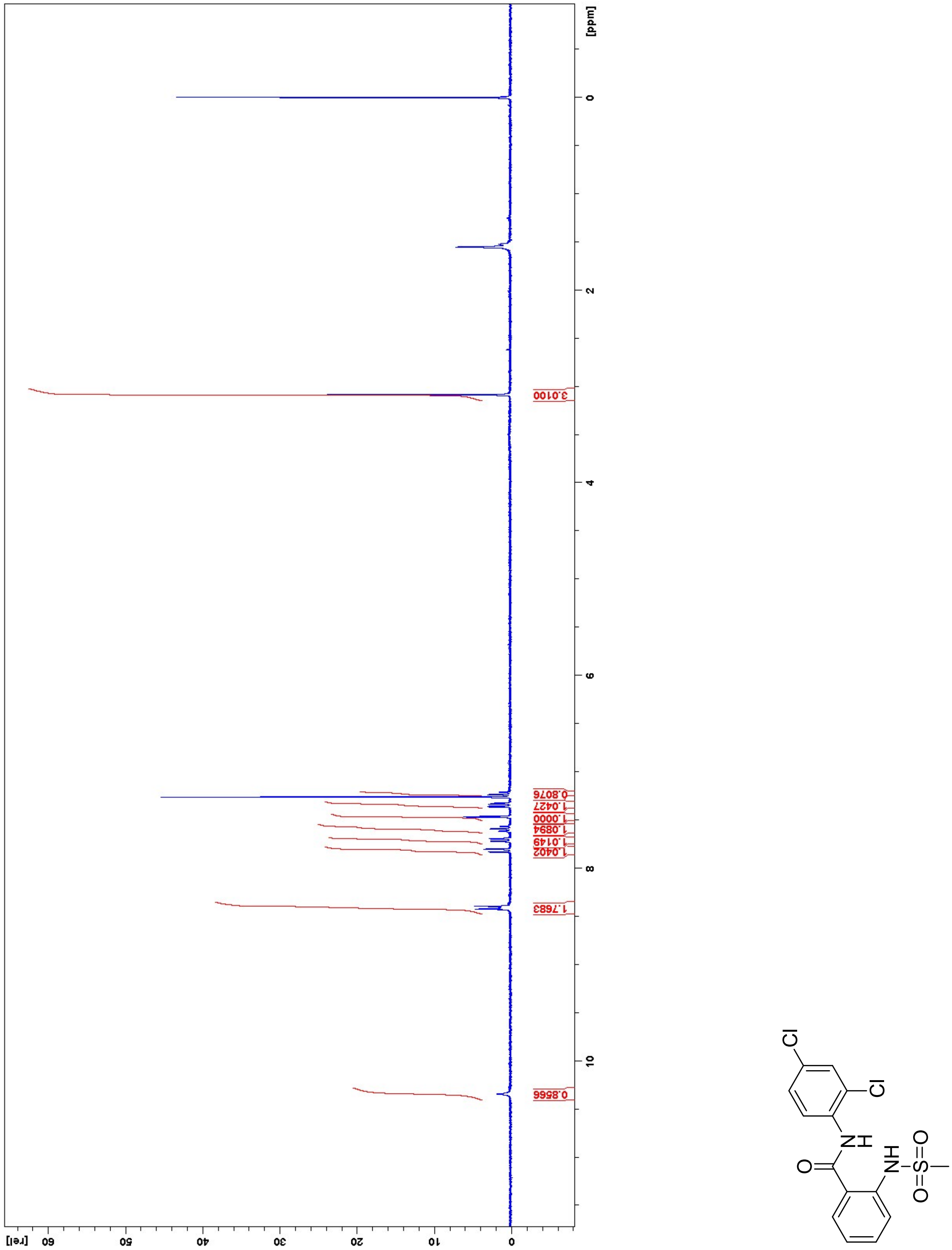

Compound **9** (^{1}H NMR, 300 MHz, DMSO-d_6)

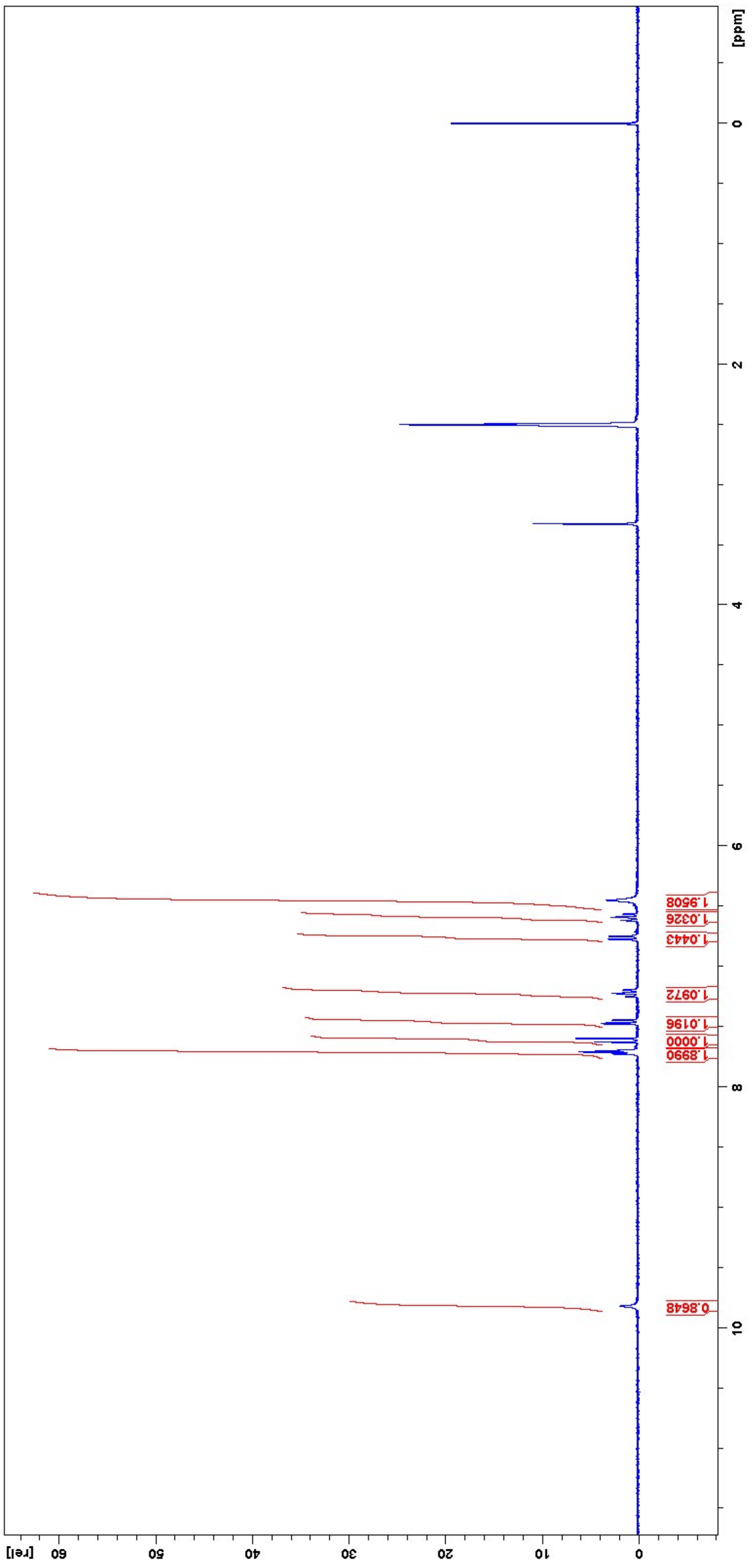

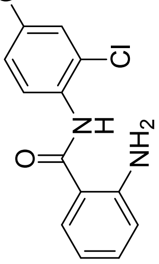

Compound **10** (^{1}H NMR, 300 MHz, $CDCl_3$)

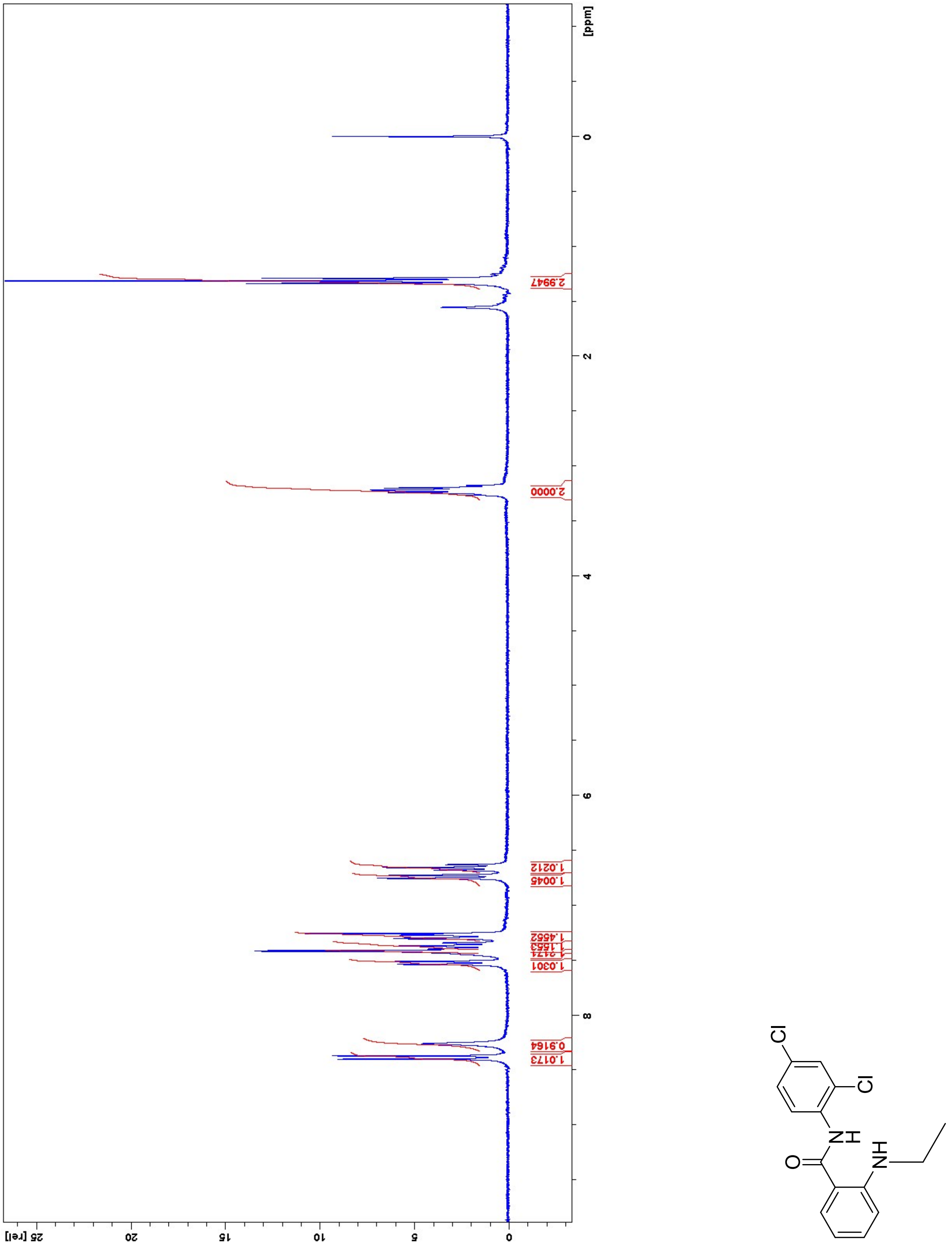

Compound **11** (^1H NMR, 300 MHz, $CDCl_3$)

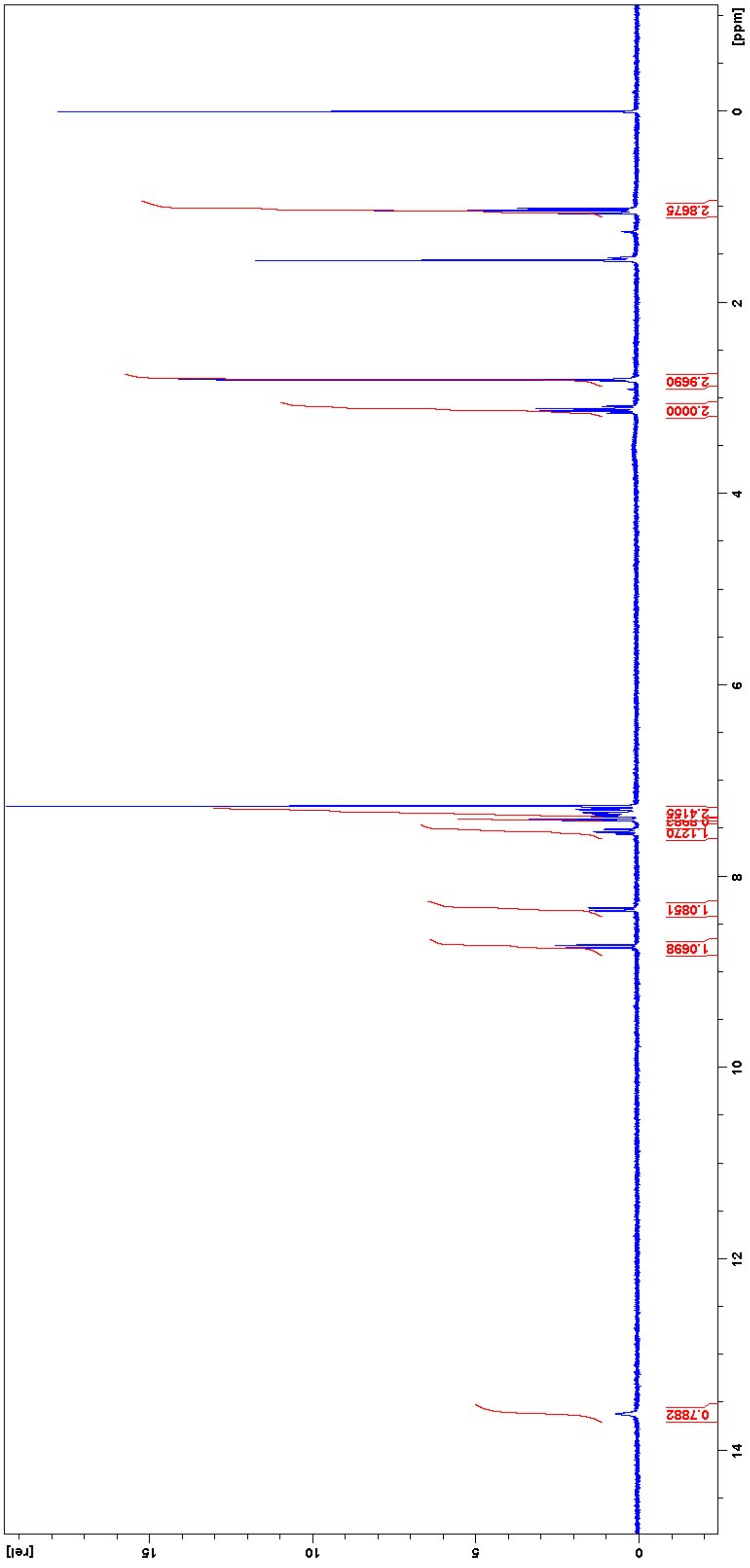

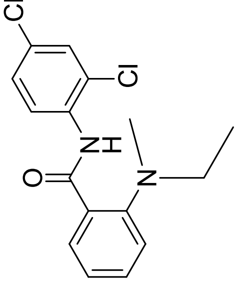

Compound **12** (^{1}H NMR, 300 M

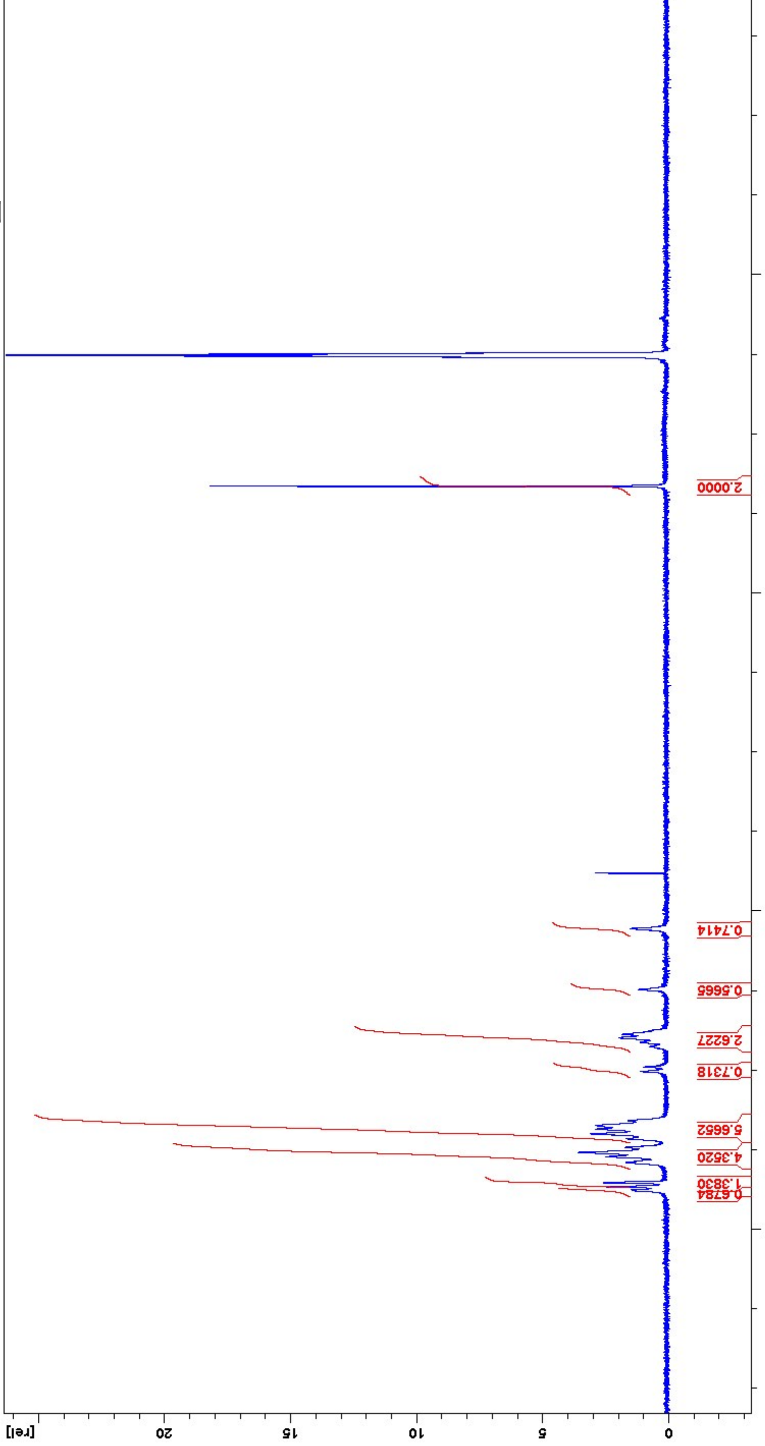

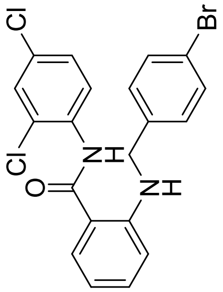

Compound **13** (^{1}H NMR, 300 MHz, $CDCl_3$)

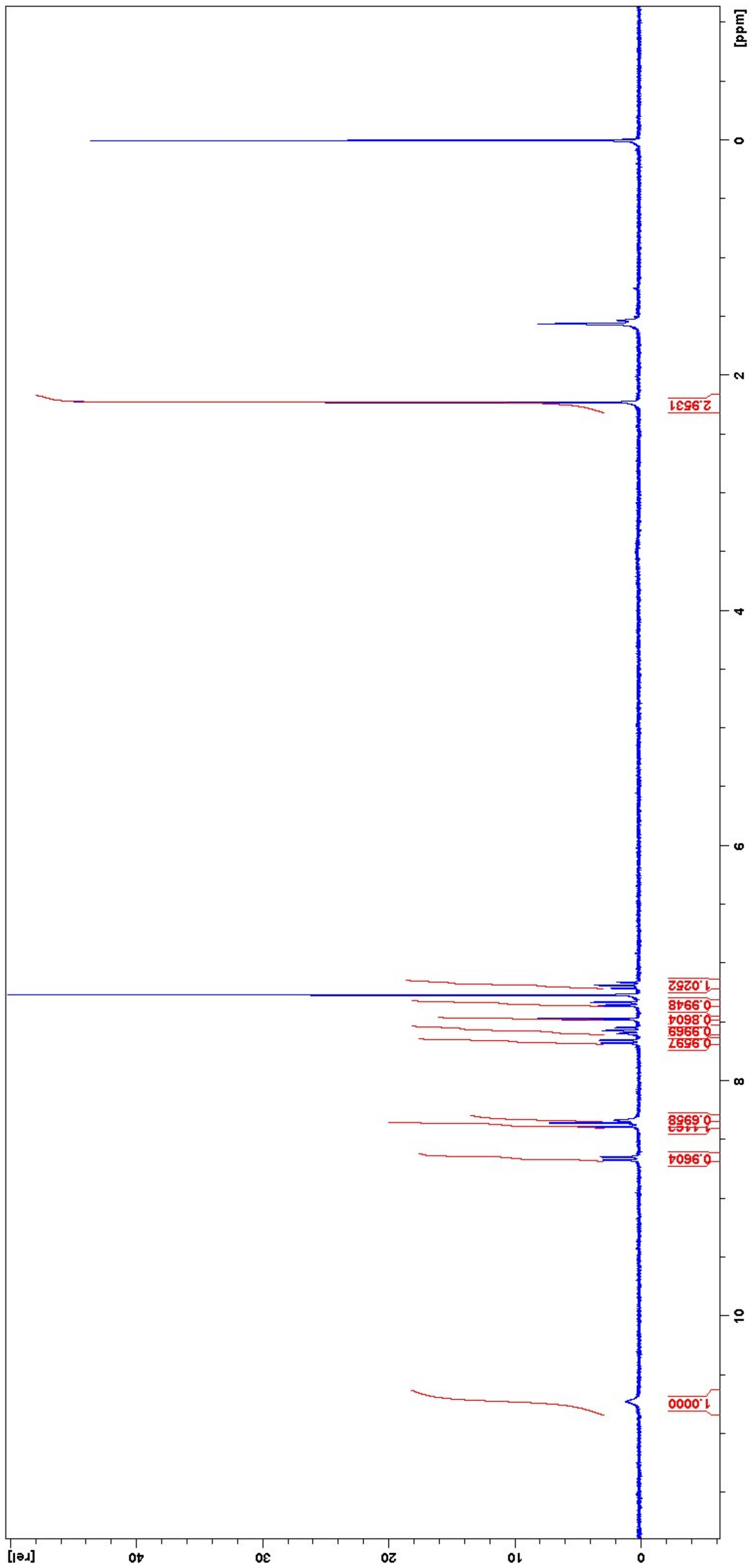

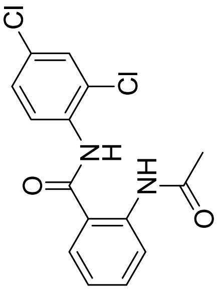

Compound **14** (^{1}H NMR, 300 MHz, $CDCl_3$)

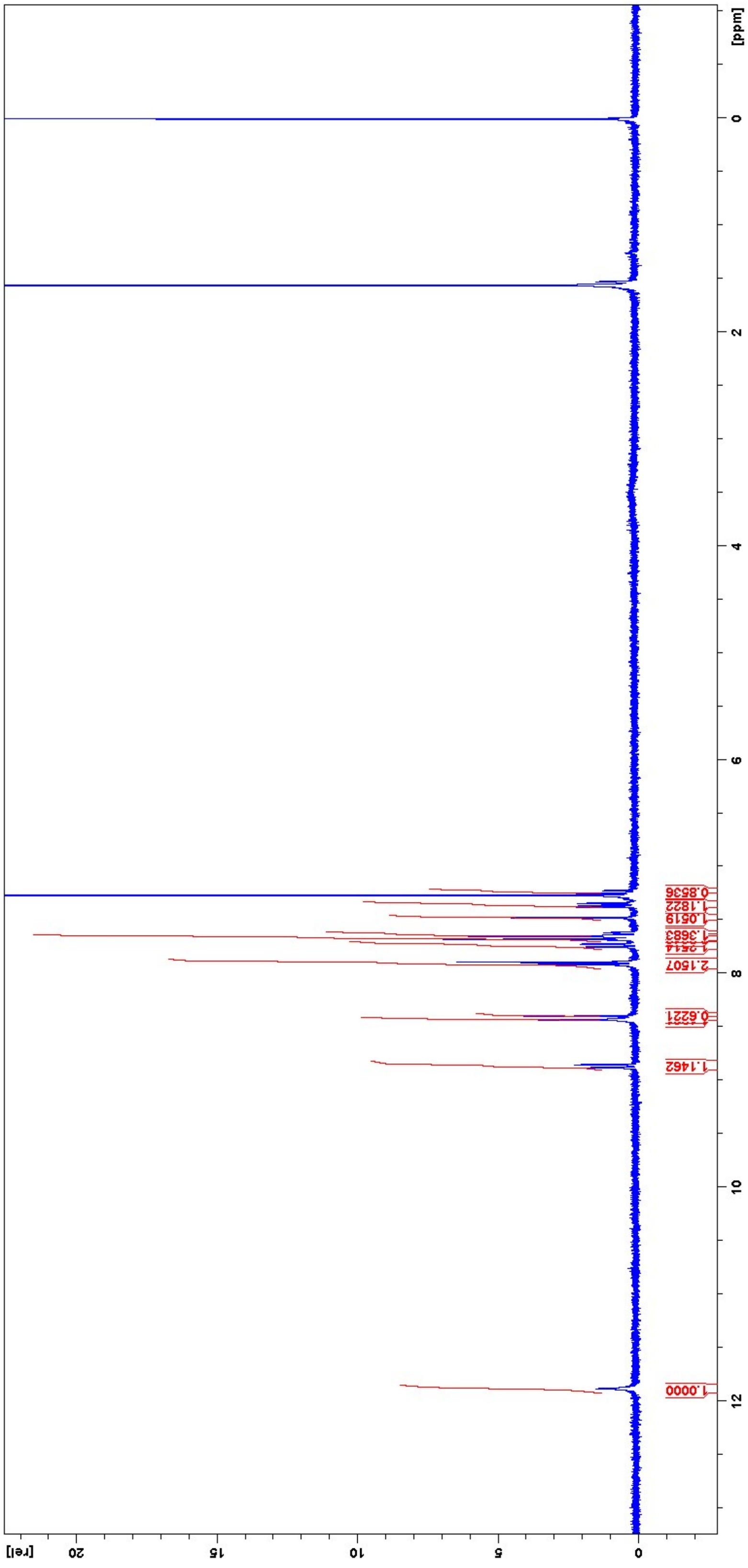

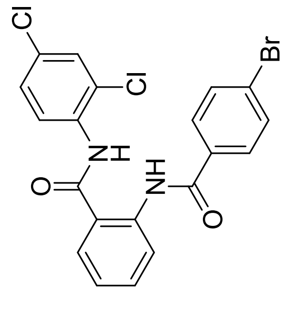

Compound **15** (^{1}H NMR, 300 MHz, $CDCl_3$)

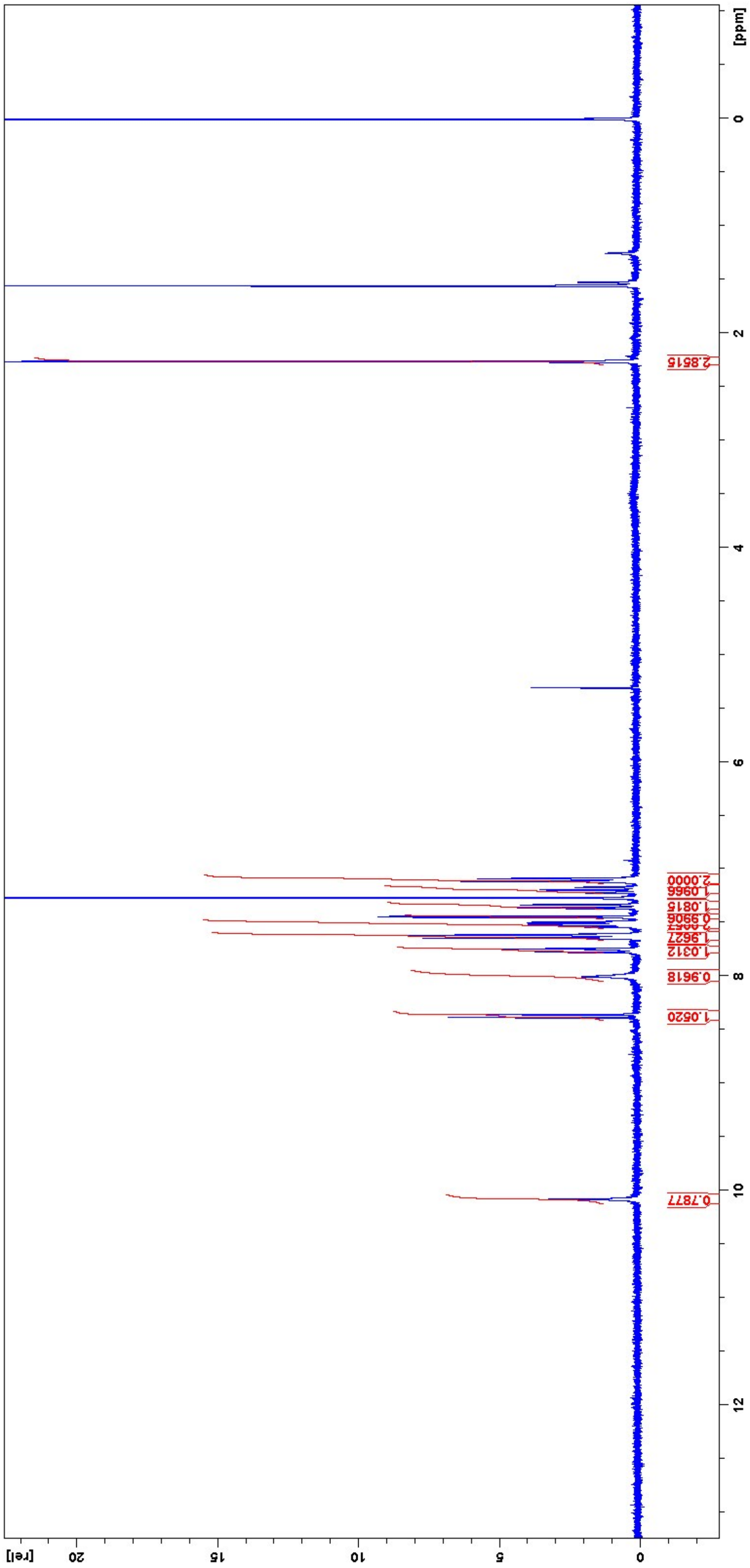

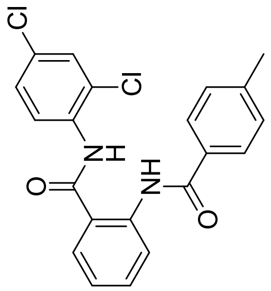

Compound **16** (^{1}H NMR, 300 MHz, $CDCl_3$)

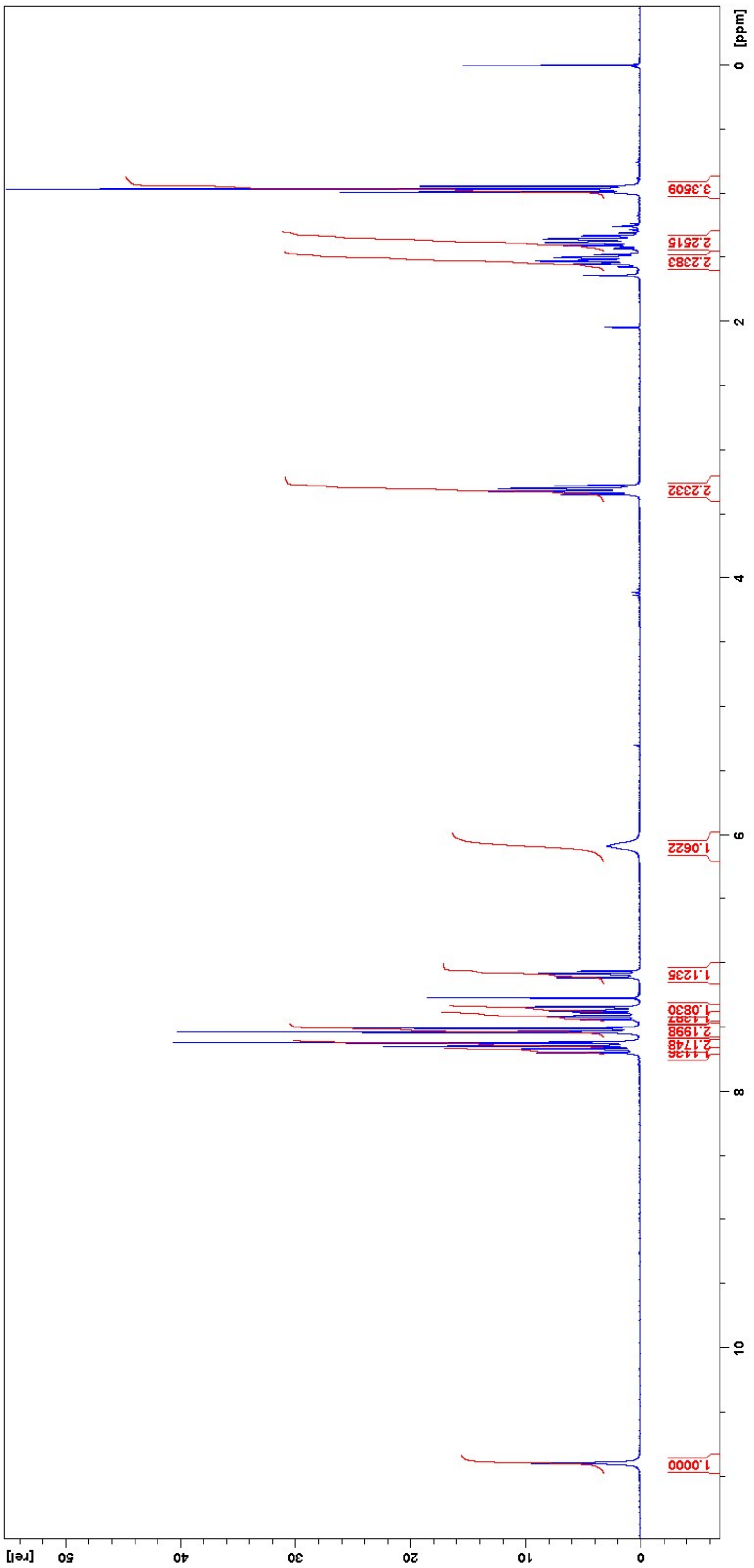

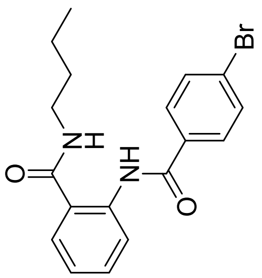

Compound **17** (^1H NMR, 300 MHz, $CDCl_3$)

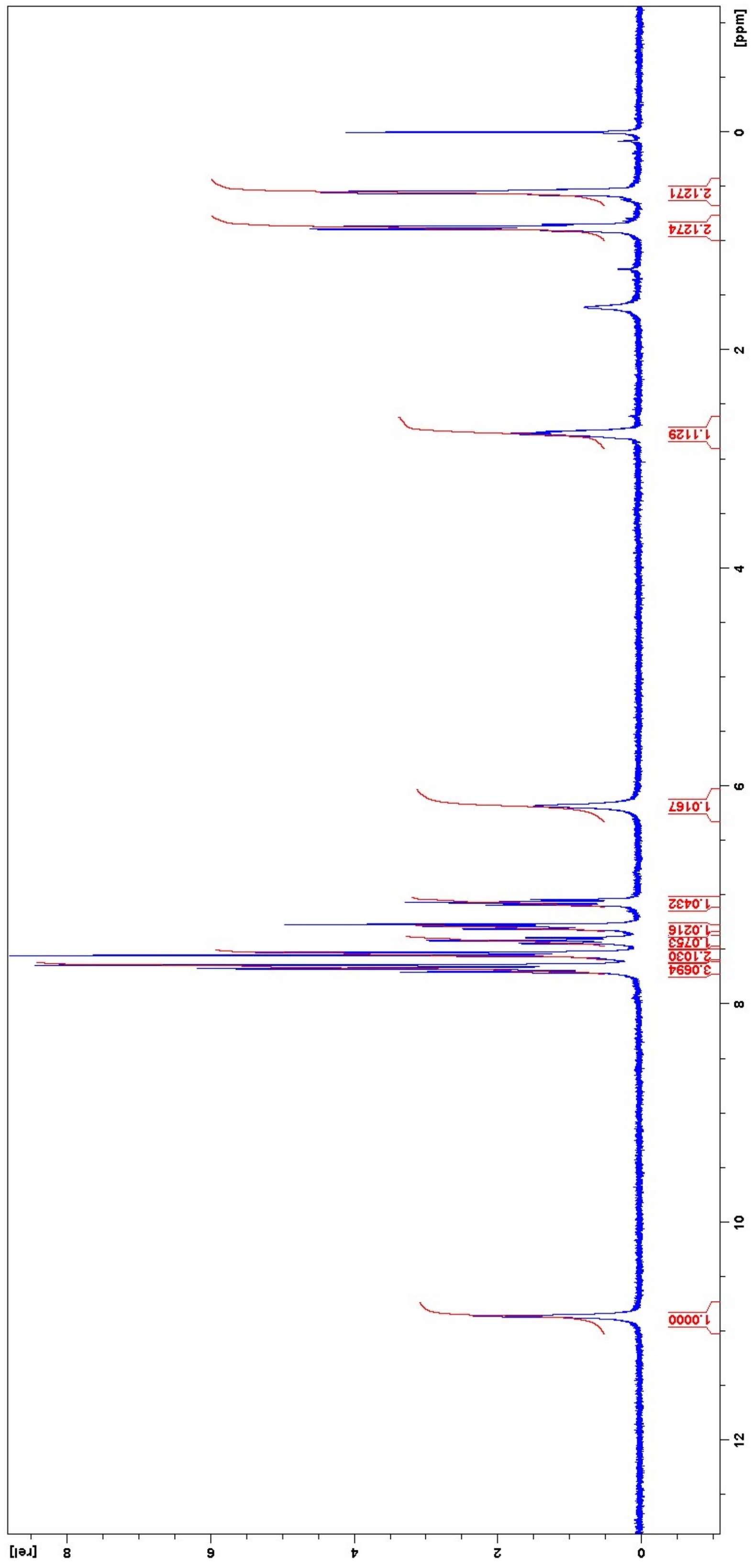

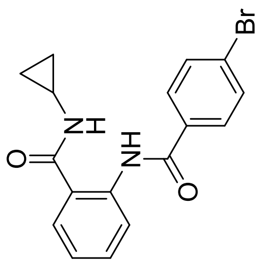

Compound **18** (^{1}H NMR, 300 MHz, $CDCl_3$)

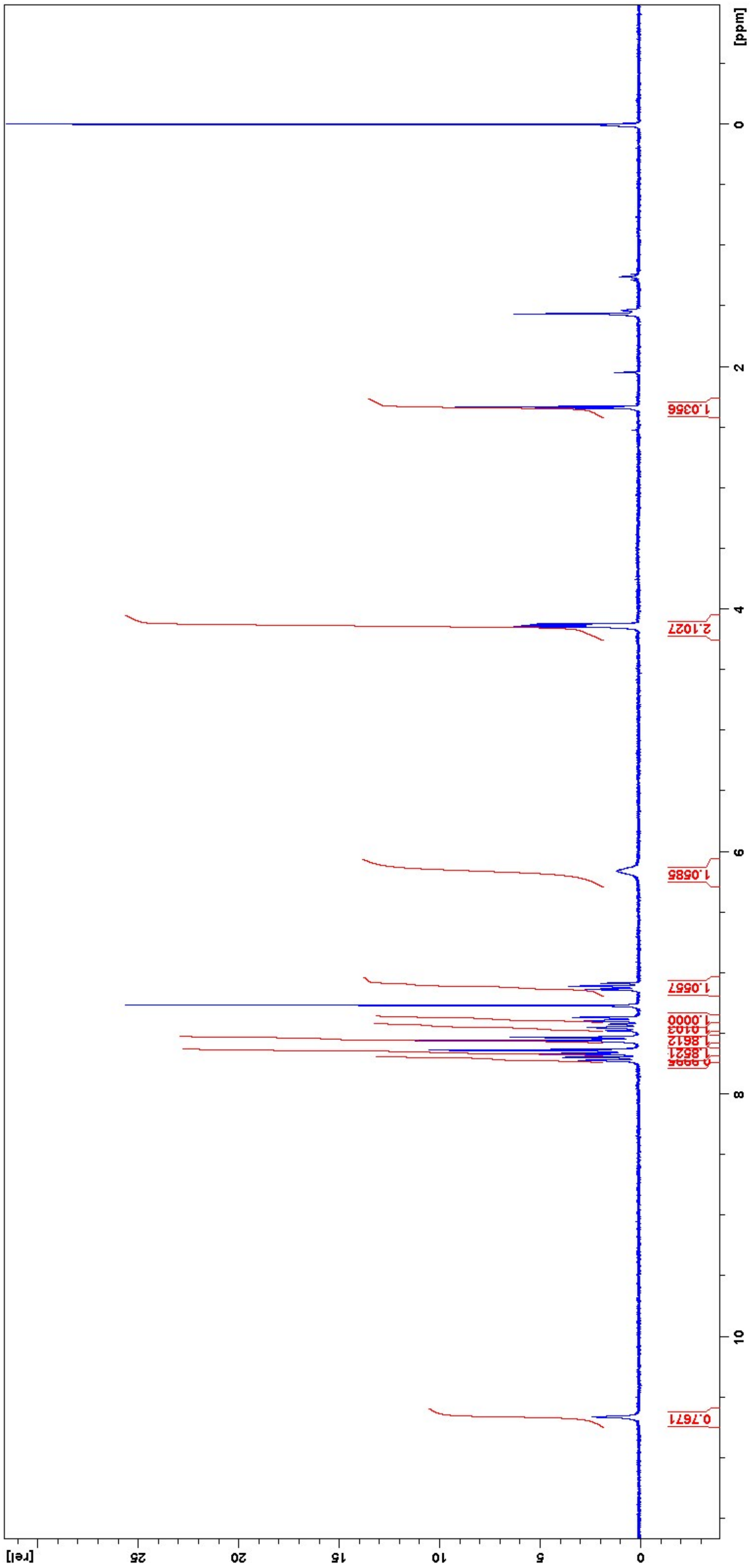

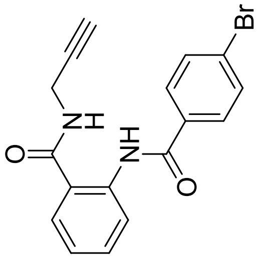

Compound **19** (^{1}H NMR, 300 M

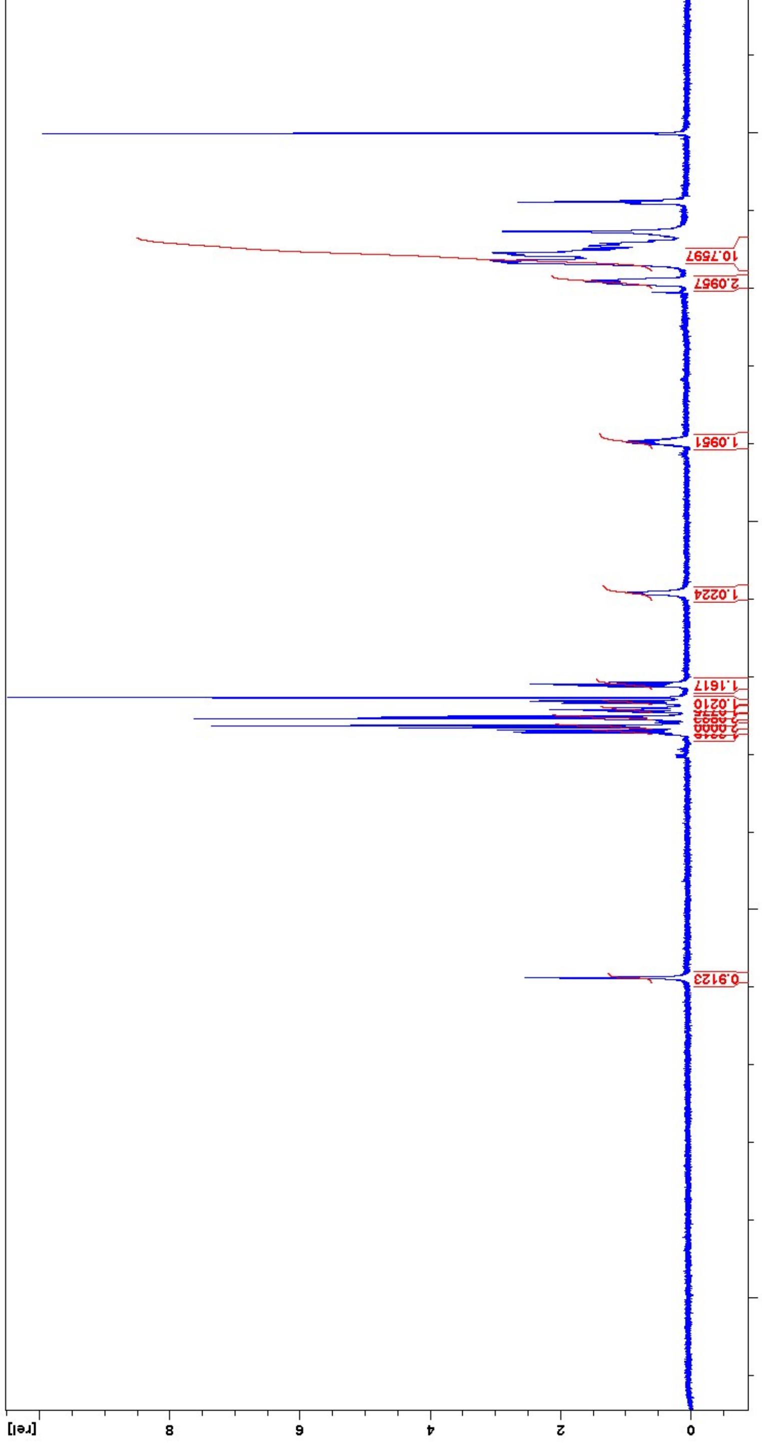

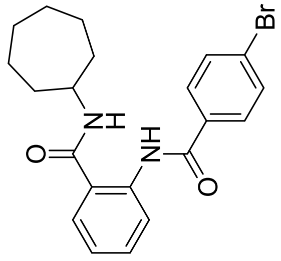

Compound **20** (^{1}H NMR, 300 MHz, $CDCl_3$)

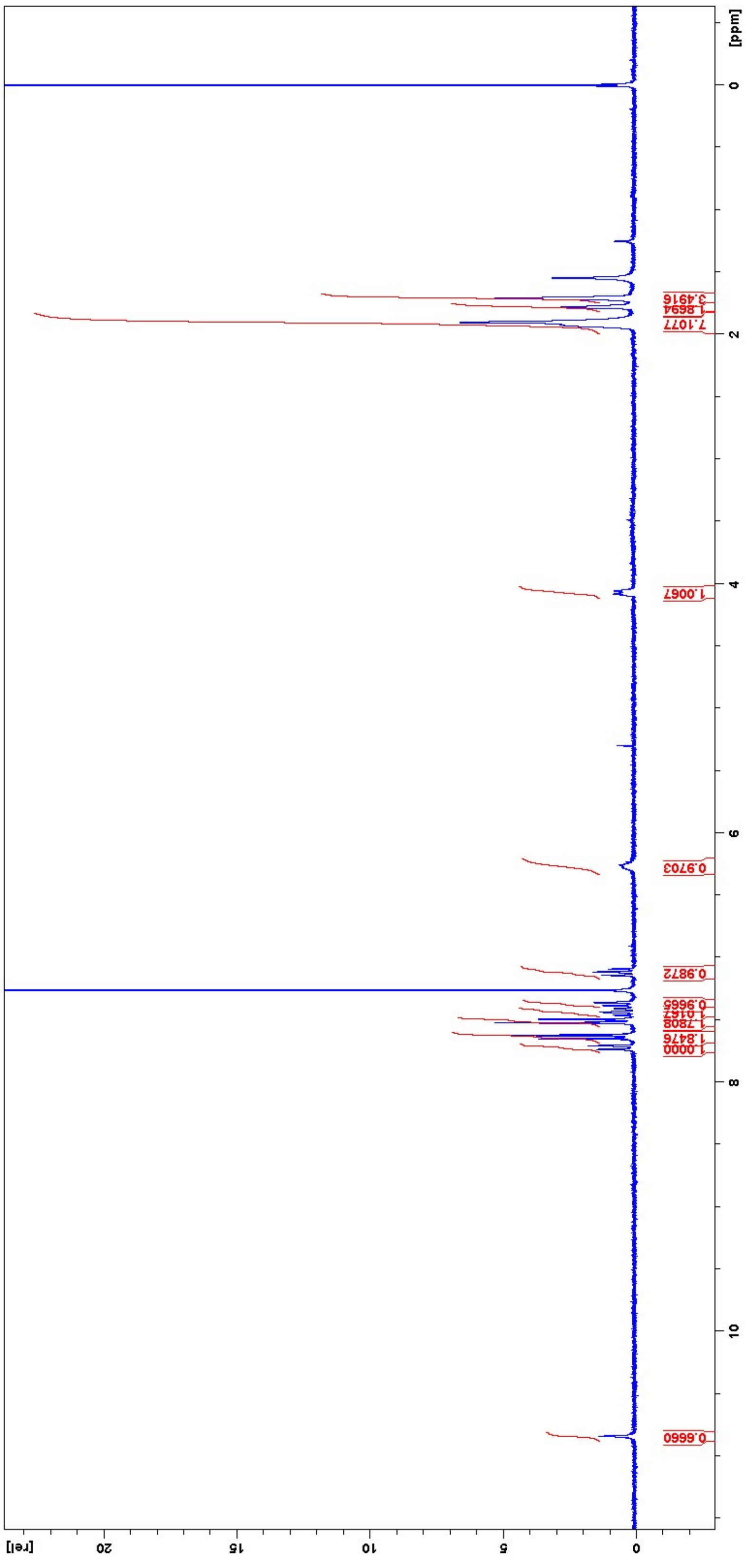

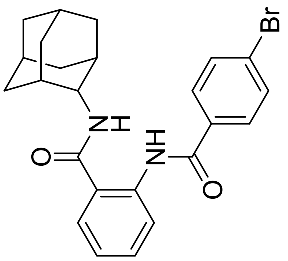

Compound **21** (^{1}H NMR, 300 MHz, $CDCl_3$)

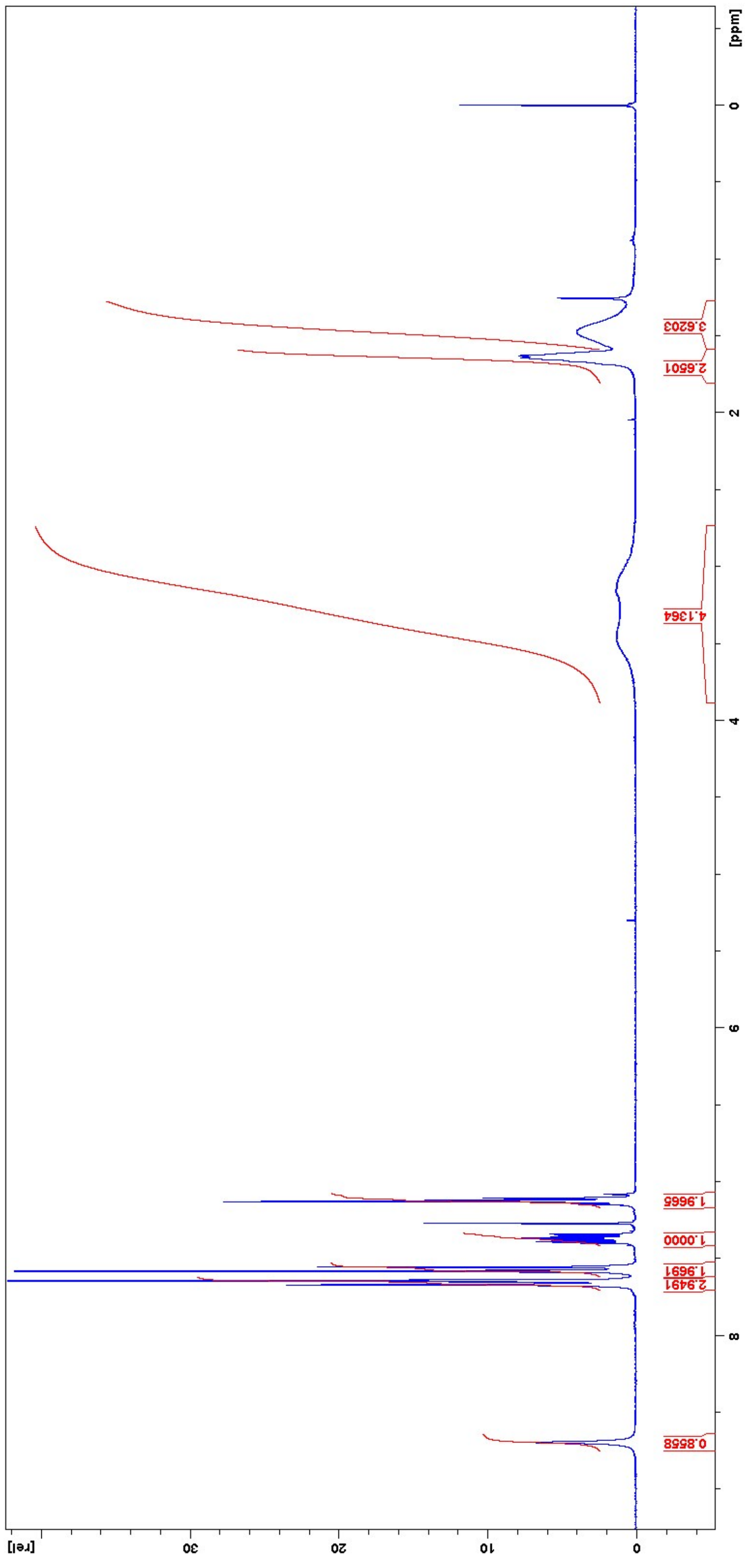

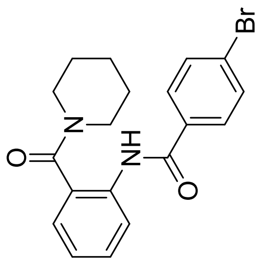

Compound **22** (^{1}H NMR, 300 MHz, $CDCl_3$)

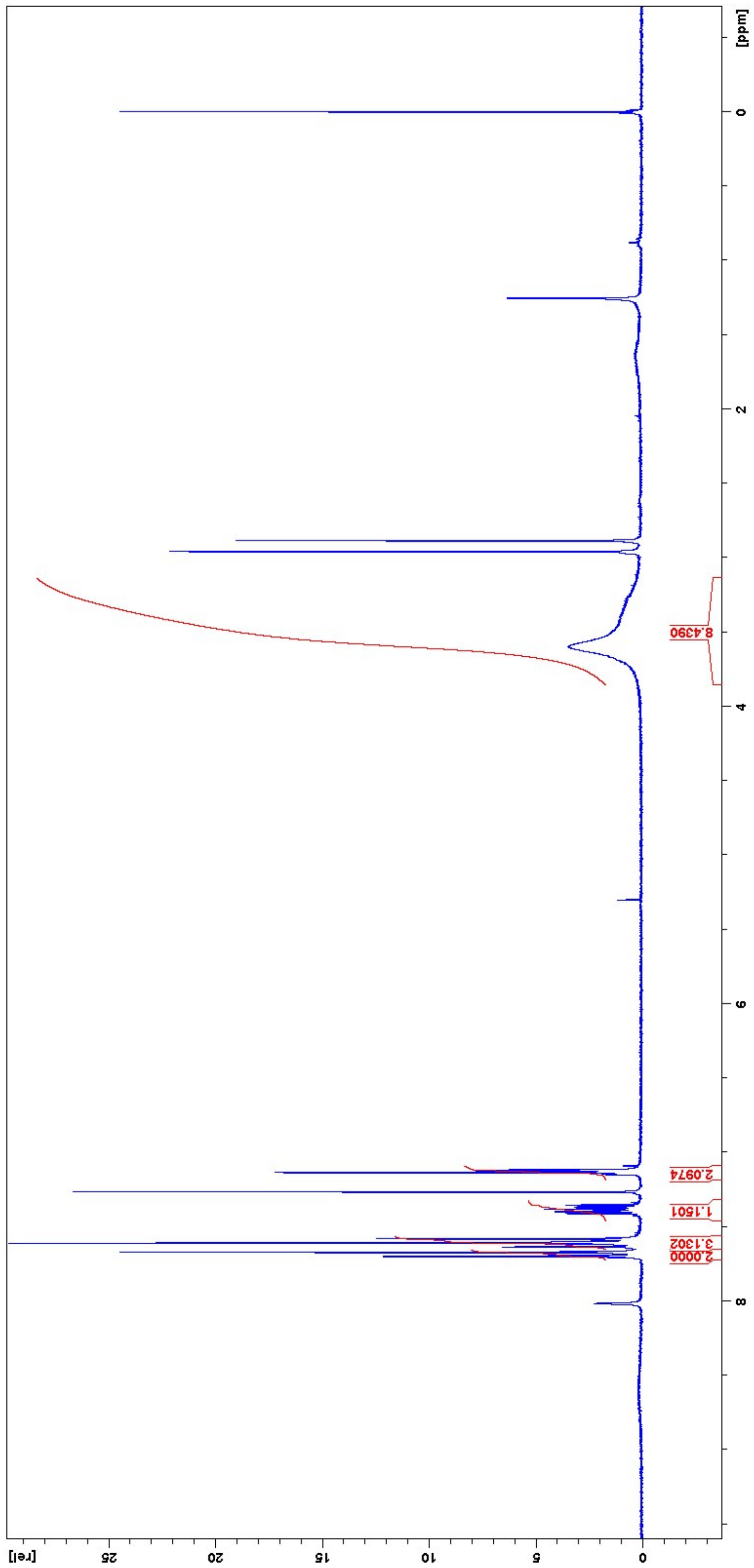

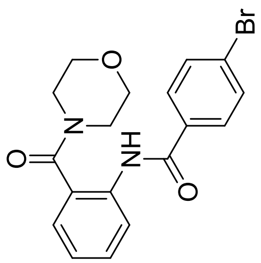

Compound **23** (^{1}H NMR, 300 MHz, $CDCl_3$)

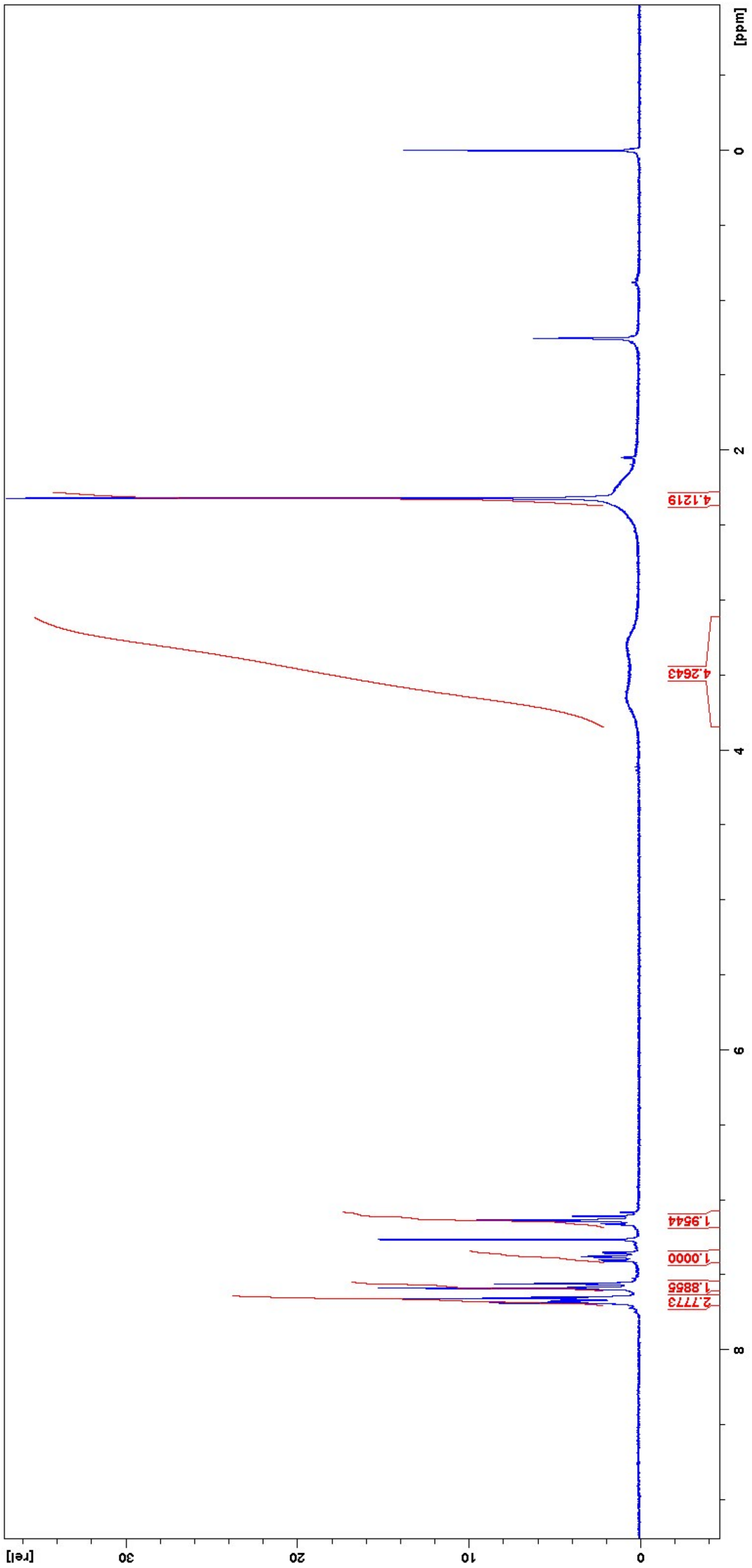

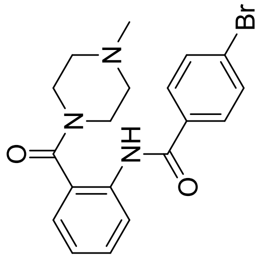

Compound **24** (^{1}H NMR, 300 MHz, $CDCl_3$)

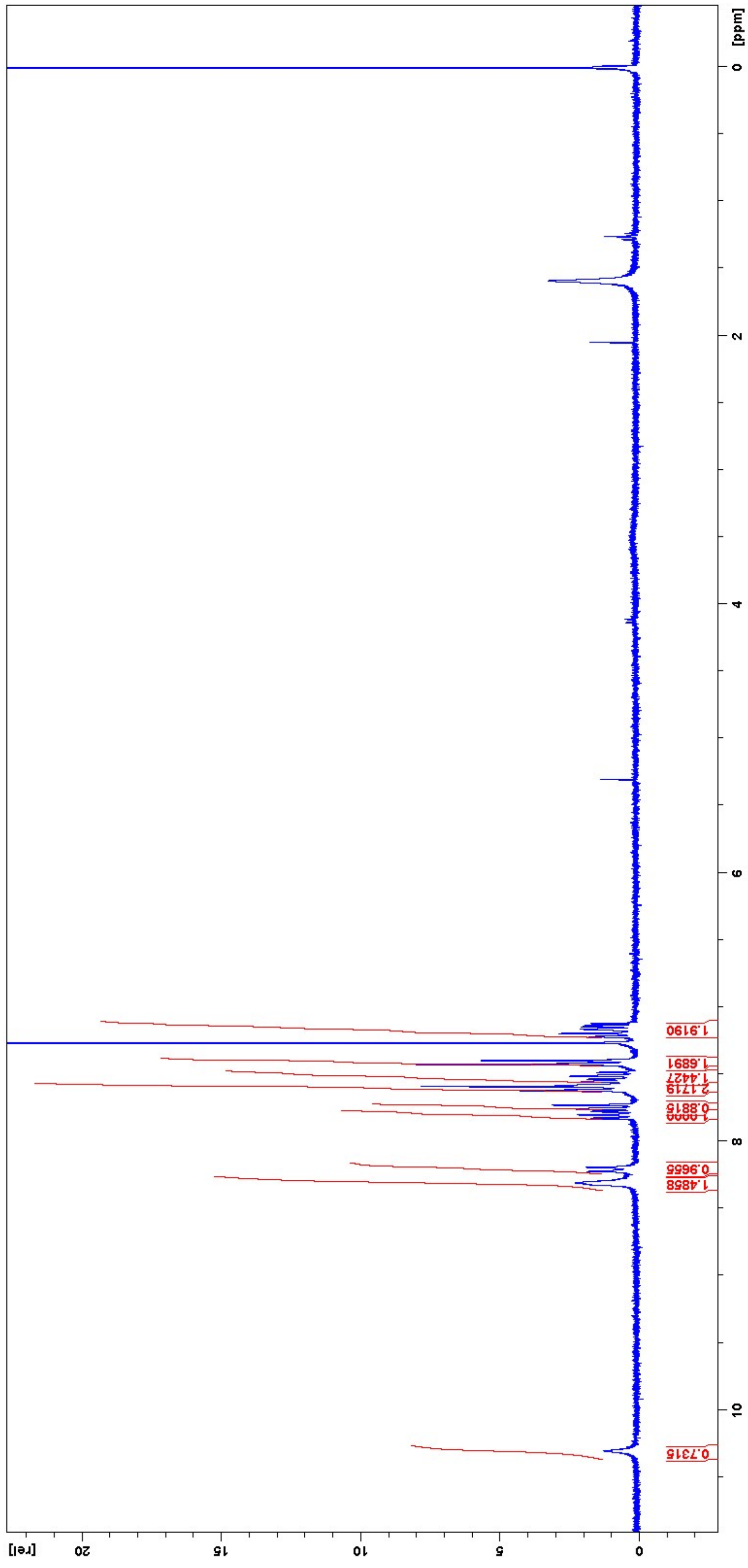

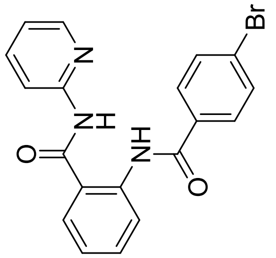

Compound **25** (^1H NMR, 300 MHz, $CDCl_3$)

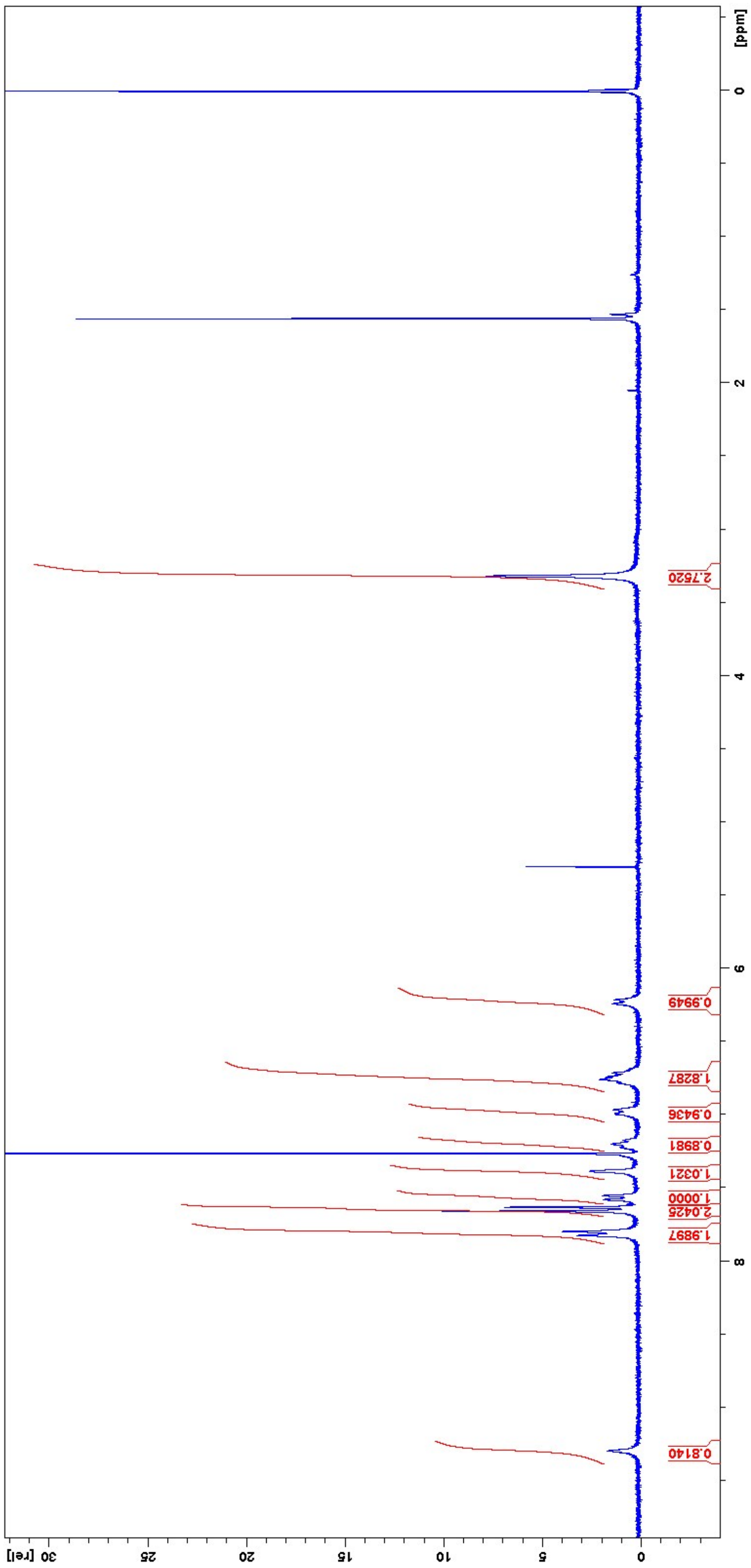

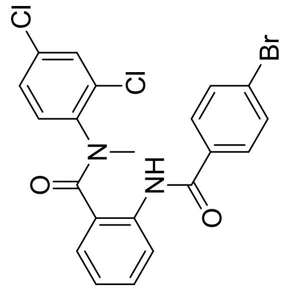

Compound **26** (^{1}H NMR, 300 MHz, DMSO-d_6)

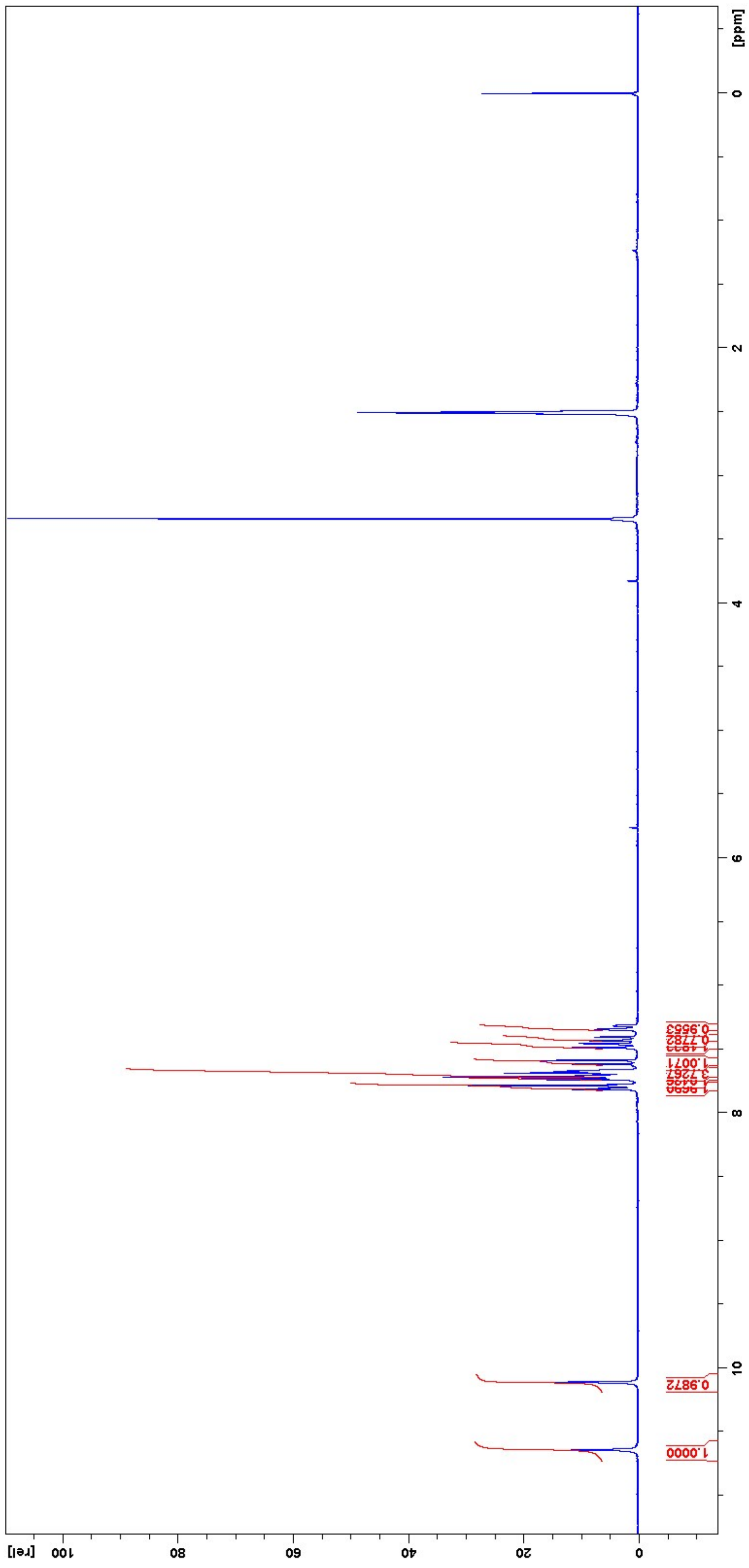

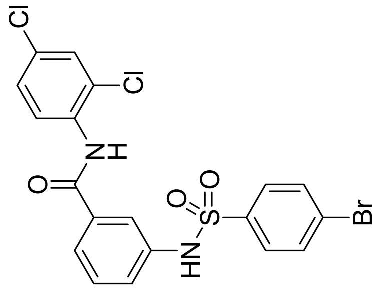

Compound **27** (^{1}H NMR, 300 MHz, $CDCl_3$)

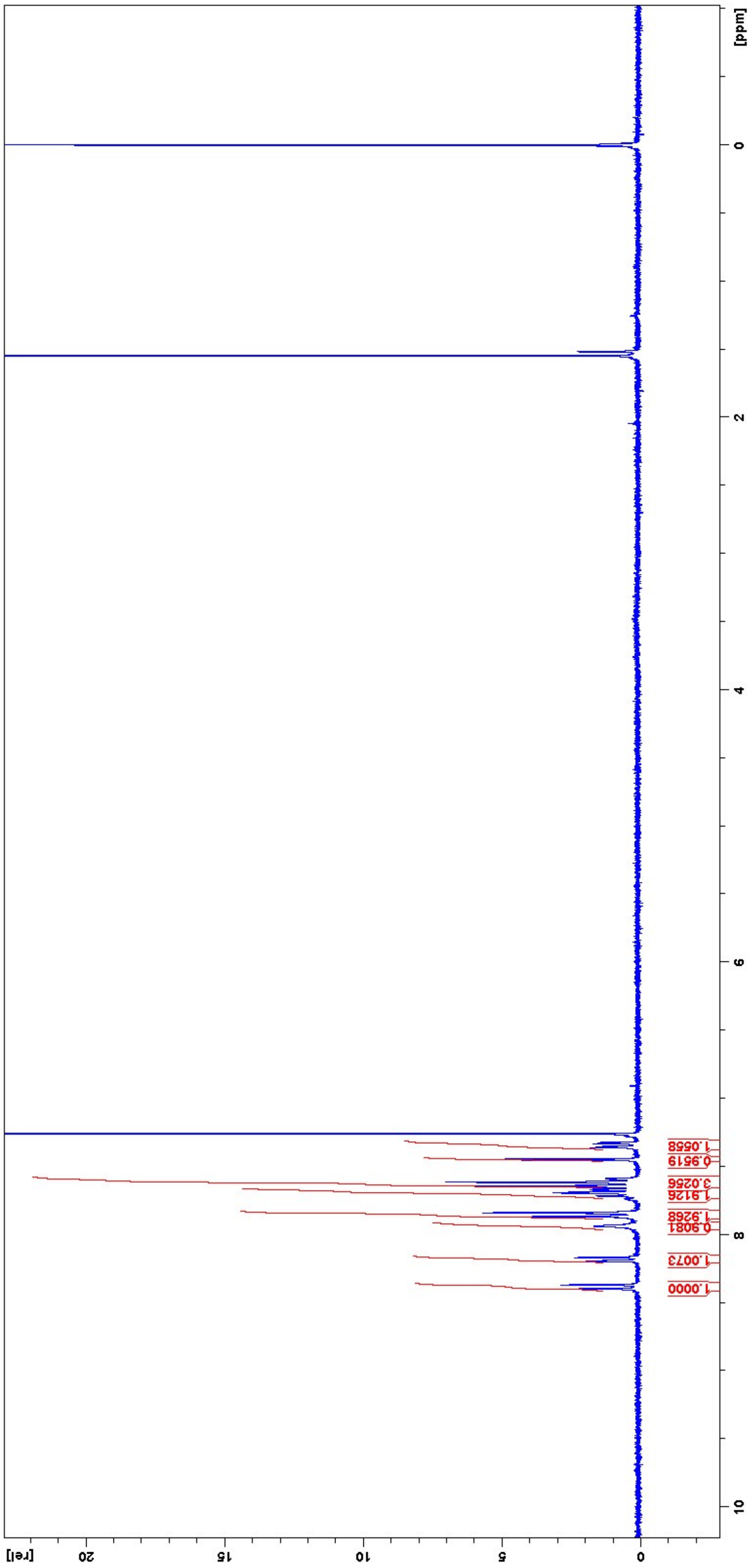

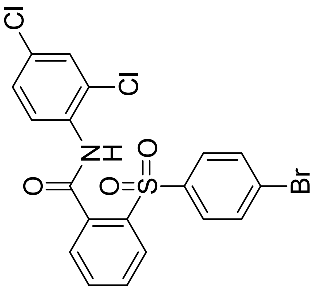

Compound **28** (^{1}H NMR, 300 MHz, $CDCl_3$)

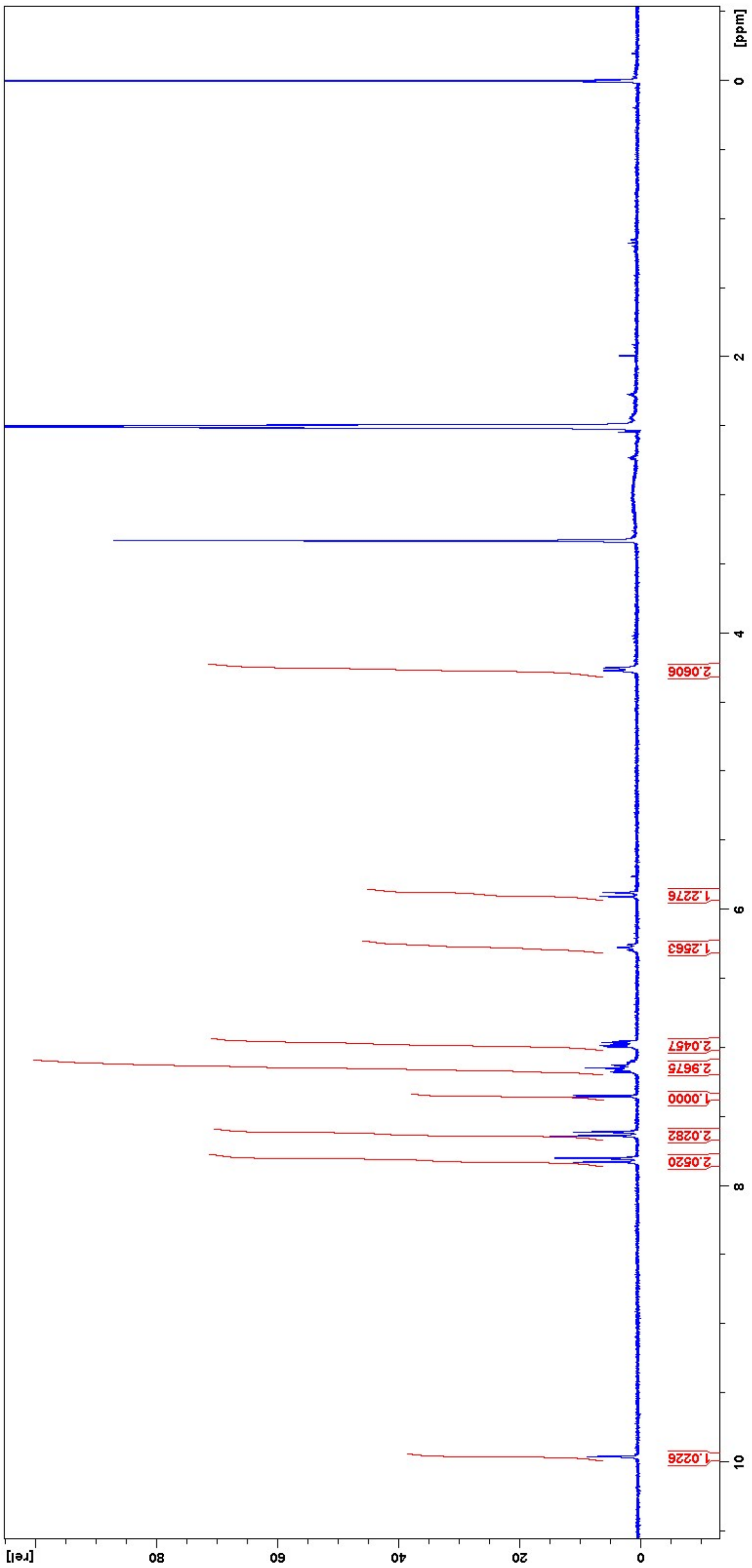

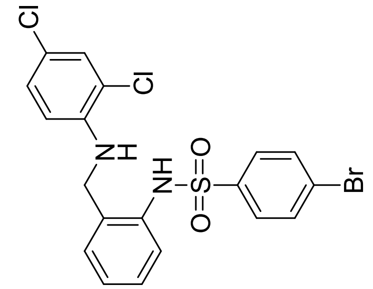

Compound **29** (^{1}H NMR, 300 MHz, $CDCl_3$)

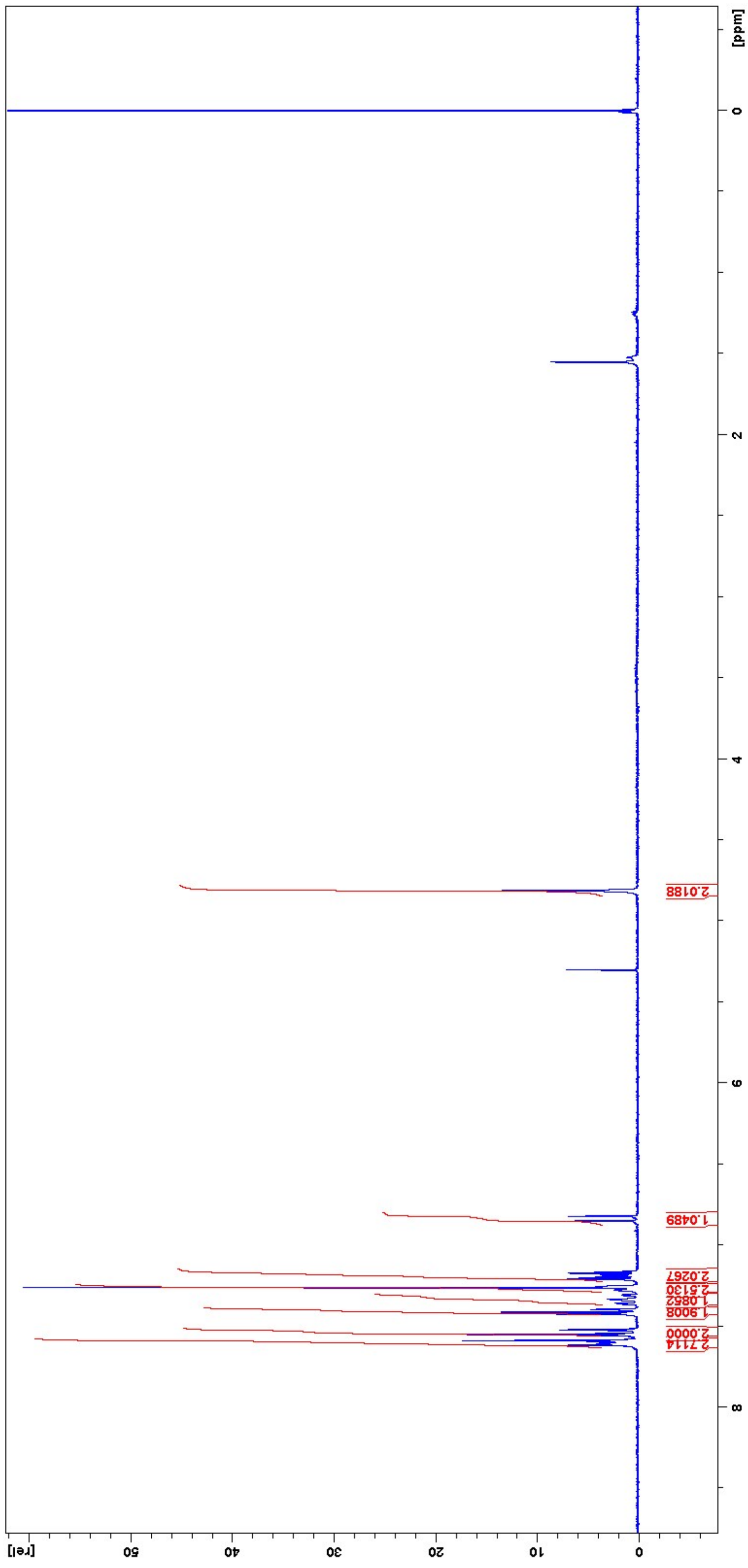

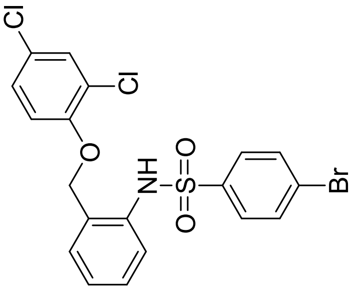

Compound **30** (^{1}H NMR, 300 MHz, DMSO-d_6)

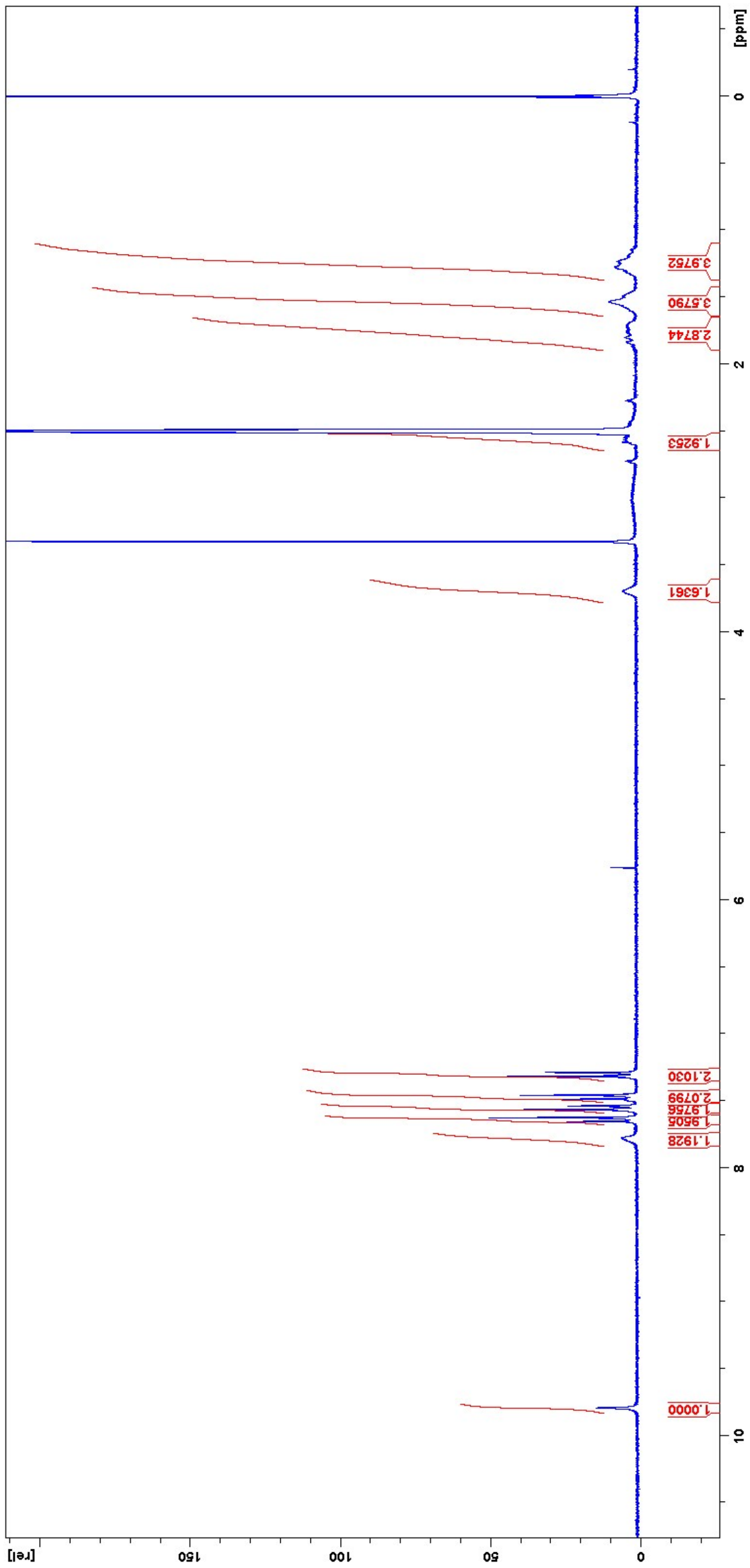

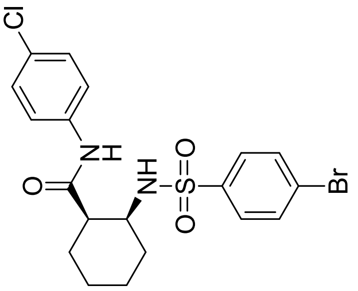

Compound **31** (^{1}H NMR, 300 MHz, DMSO-d_6)

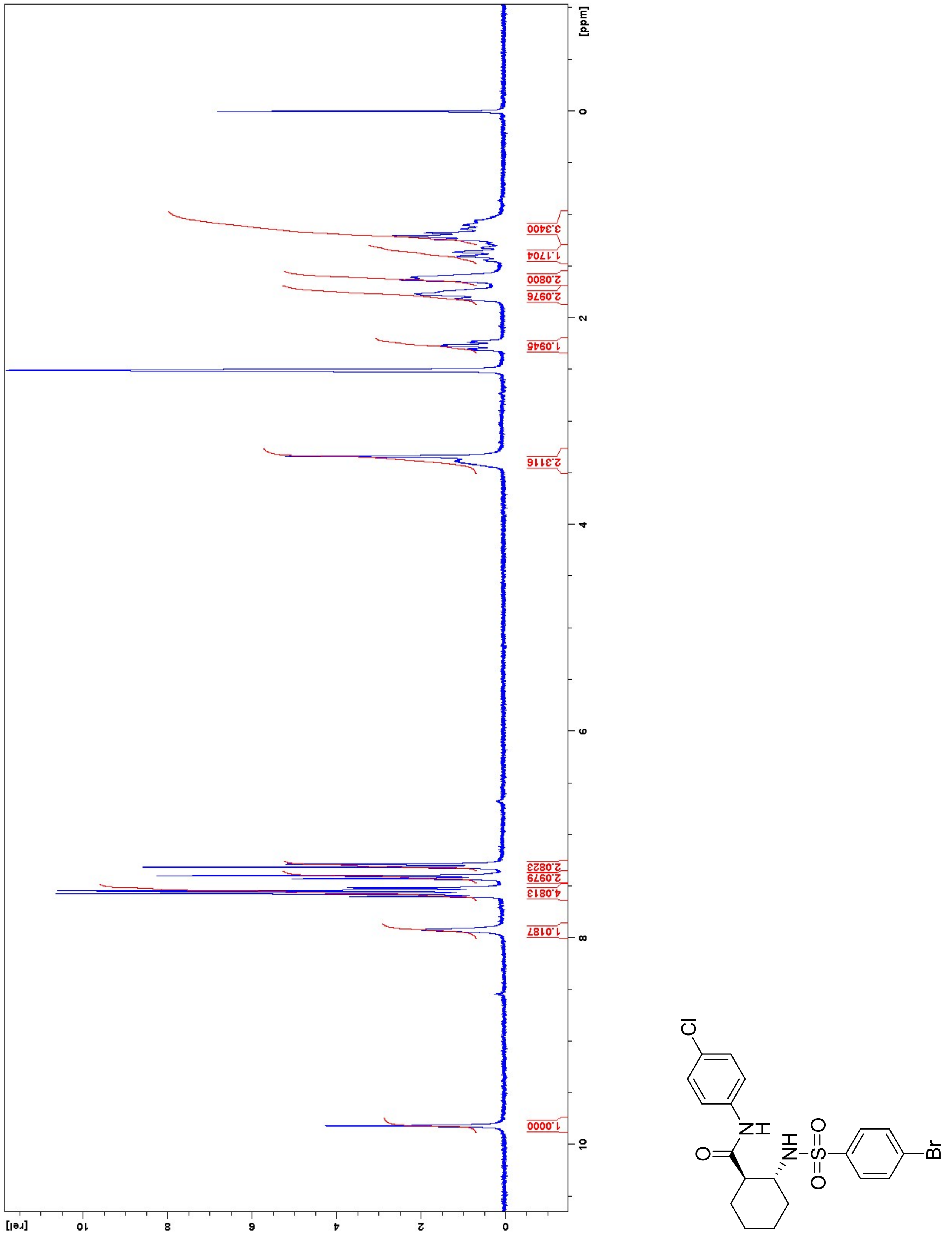

Compound **32** (^{1}H NMR, 300 MHz, $CDCl_3$)

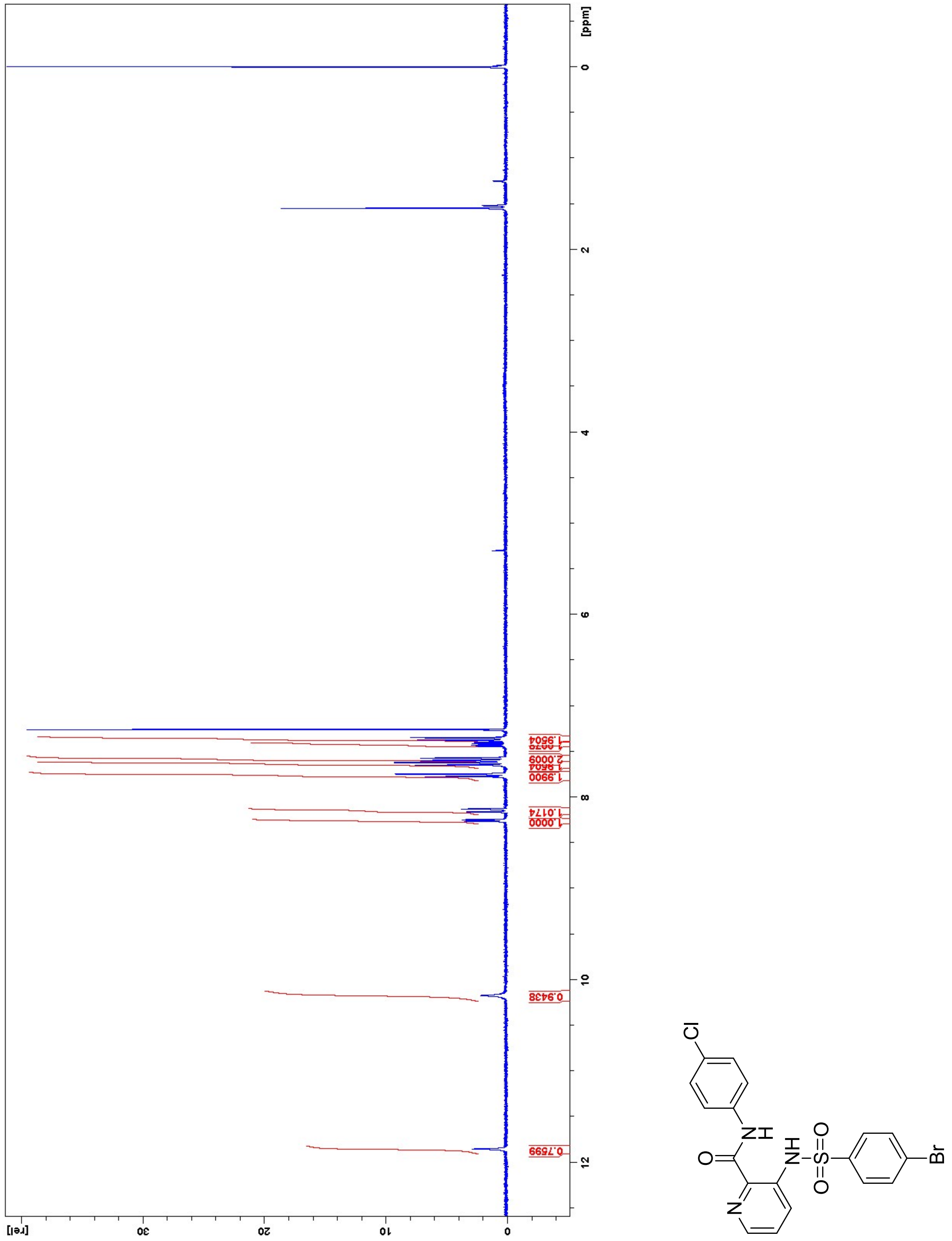

Compound **33** (^{1}H NMR, 300 MHz, $CDCl_3$)

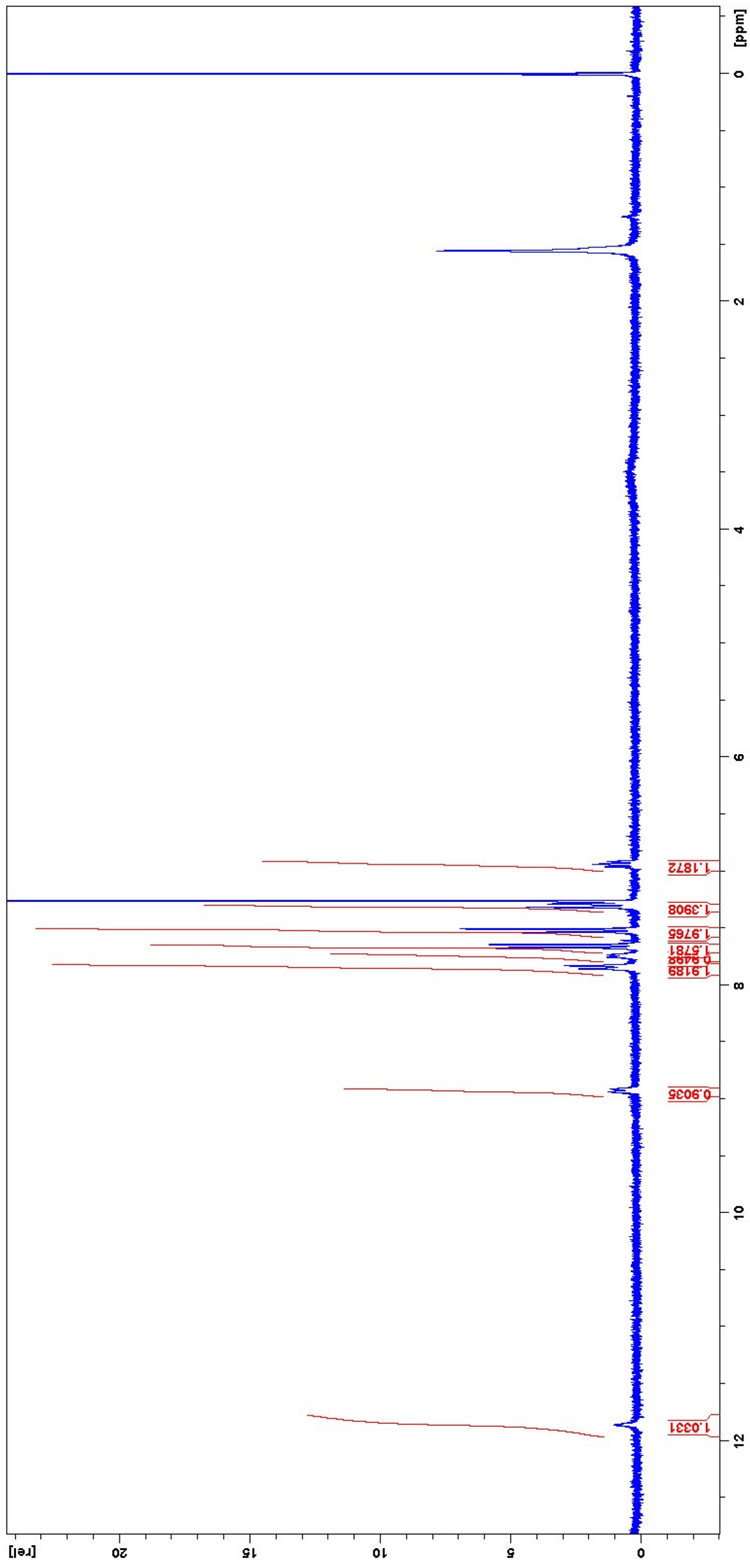

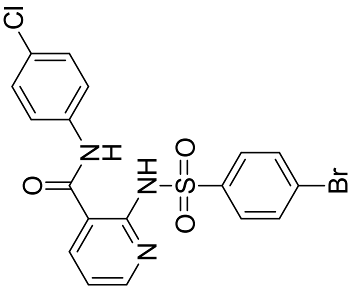

Supplement: Supplementary file 1 [file MD-010-C9MD00088G-s001.pdf]
